# Evolution of *Stenotrophomonas maltophilia* in Cystic Fibrosis Lung over Chronic Infection: A Genomic and Phenotypic Population Study

**DOI:** 10.3389/fmicb.2017.01590

**Published:** 2017-08-28

**Authors:** Alfonso Esposito, Arianna Pompilio, Clotilde Bettua, Valentina Crocetta, Elisabetta Giacobazzi, Ersilia Fiscarelli, Olivier Jousson, Giovanni Di Bonaventura

**Affiliations:** ^1^Centre for Integrative Biology, University of Trento Trento, Italy; ^2^Department of Medical, Oral, and Biotechnological Sciences, Center of Excellence on Aging and Translational Medicine (CeSI-MeT), Università degli Studi “G. d'Annunzio” Chieti-Pescara Chieti, Italy; ^3^Laboratory of Cystic Fibrosis Microbiology, “Bambino Gesù” Hospital Rome, Italy

**Keywords:** *Stenotrophomonas maltophilia*, cystic fibrosis, evolution, genomics, longitudinal study

## Abstract

*Stenotrophomonas maltophilia* has been recognized as an emerging multi-drug resistant opportunistic pathogen in cystic fibrosis (CF) patients. We report a comparative genomic and phenotypic analysis of 91 *S. maltophilia* strains from 10 CF patients over a 12-year period. Draft genome analyses included *in silico* Multi-Locus Sequence Typing (MLST), Single-Nucleotide Polymorphisms (SNPs), and pangenome characterization. Growth rate, biofilm formation, motility, mutation frequency, *in vivo* virulence, and *in vitro* antibiotic susceptibility were determined and compared with population structure over time. The population consisted of 20 different sequence types (STs), 11 of which are new ones. Pangenome and SNPs data showed that this population is composed of three major phylogenetic lineages. All patients were colonized by multiple STs, although most of them were found in a single patient and showed persistence over years. Only few phenotypes showed some correlation with population phylogenetic structure. Our results show that *S. maltophilia* adaptation to CF lung is associated with consistent genotypic and phenotypic heterogeneity. *Stenotrophomonas maltophilia* infecting multiple hosts likely experiences different selection pressures depending on the host environment. The poor genotype-phenotype correlation suggests the existence of complex regulatory mechanisms that need to be explored in order to better design therapeutic strategies.

## Introduction

Cystic fibrosis (CF) is a life-limiting autosomal recessive disorder caused by a mutation targeting the Cystic Fibrosis Transmembrane conductance Regulator (CFTR) gene. This leads to altered regulation of ion transport homeostasis in epithelial cells, particularly in the airways where impaired mucociliary clearance and antimicrobial defense create a perfect niche for microbial colonization.

*Pseudomonas aeruginosa* and *Staphylococcus aureus* are the most common bacterial pathogens isolated from the CF respiratory tract where they cause persistent infections associated with a more rapid decline in lung function and survival (Emerson et al., 2002). However, the selective pressure exerted on bacterial populations mainly by the antipseudomonal antibiotic therapy in recent years led to an increasing number of reports on potentially emerging and challenging pathogens (Parkins and Floto, 2015). In this context, both the overall prevalence and incidence of *Stenotrophomonas maltophilia* isolations from CF respiratory tract secretions have increased steadily in recent years. Despite this, the clinical relevance of *S. maltophilia* remains undetermined because of conflicting results arising from clinical studies focused on the correlation between infection and lung damage (Colin and Rabin, 2011).

However, recent studies showed that in CF individuals, chronic pulmonary infection with *S. maltophilia* is associated with an increased risk of pulmonary exacerbations, lung transplantation, and death (Waters et al., 2013). In the past years, we gained *in vitro* and *in vivo* evidence for the pathogenetic potential of *S. maltophilia* in these patients (Pompilio et al., 2008, 2010, 2015; Di Bonaventura et al., 2010). In particular, the persistence of microorganism in CF lung is probably due to its ability to colonize bronchial CF-derived cells in the form of biofilms, sessile communities inherently resistant to both antibiotics and host immunity (Pompilio et al., 2010), probably as a consequence of a selective adaptation to CF airways. *S. maltophilia* biofilms are intrinsecally resistant to antibiotics, being not eradicated also following exposure to bactericidal concentrations (Di Bonaventura et al., 2004).

During a chronic colonization, pathogens adapt over time to cope with changing selection pressures, co-infecting species, and antibiotic therapies (Cullen and McClean, 2015). In the lung, these adaptations are induced by environmental pressures, such as, inflammatory responses, hypoxia, nutrient deficiency, osmolarity, low pH, and antibiotic therapies. The evolution of *P. aeruginosa* during chronic lung infection has been widely studied. The main phenotypic adaptations occurring during transition from acute to chronic *P. aeruginosa* infection include acquisition of antibiotic resistance, exopolysaccharide production (mucoidy), loss in motility, formation of small colony variants, increased mutation rate, quorum sensing, and altered production of virulence factors (Clark et al., 2015; Cullen and McClean, 2015).

More recently, the adaptation *S. maltophilia* undergoes during chronic infection has also been investigated. For the first time, genotypic diversity, mutation frequency, and antibiotic resistance in 90 *S. maltophilia* isolates chronically colonizing 19 CF patients have been studied (Vidigal et al., 2014). They found that *S. maltophilia* adaptation during chronic CF lung infection leads to high genetic diversity, although a decreased mutation rate was observed in the late isolates. Similarily, previous epidemiological studies of *S. maltophilia* populations showed that there is high genetic variability among clones, and that environmental strains may carry an extended antibiotic-resistance gene set (Valdezate et al., 2004; Youenou et al., 2015). The forces shaping the genetic diversity of this species are mainly driven by homologous recombination, followed by gene gain and loss events (Yu et al., 2016). Recently, we comparatively evaluated 13 *S. maltophilia* strains isolated from the same CF chronically infected patient over a 10-year period for growth rate, biofilm formation, motility, mutation frequencies, antibiotic resistance, and pathogenicity (Pompilio et al., 2016). *S. maltophilia* adaptation leads to increased antibiotic resistance, but decreased *in vivo* pathogenicity as well as biofilm formation. However, having considered only one chronically infected patient did not allow us to evaluate if the adaptation process may relate to the complexity of the individual host niche.

For these reasons, in order to gain new and improved knowledge on the genetic and phenotypic basis underlying *S. maltophilia* evolution in CF airways, 91 *S. maltophilia* strains collected over a 12 year-period from 10 CF patients at a single hospital were considered in the present work. Each strain was evaluated for several phenotypic traits, namely growth rate, biofilm formation, motility (swimming, swarming, twitching), mutation frequency, *in vivo* virulence, and *in vitro* antibiotic susceptibility. A genome-wide analysis of mutations among strains was also performed, aimed at elucidating genetic diversity and phylogenetic relationships within this population. Temporal trends in phenotypic traits in relation to the genetic background of the strains were then pointed out.

## Materials and methods

### Ethic statement

This study was carried out in accordance with the recommendations of the Ethical Committee of the Children's Hospital “Bambino Gesù,” Rome, Italy, project 1413_OPBG_2017 “Evaluation of mechanisms underlying *S. maltophilia* adaptation to cystic fibrosis lung during chronic infection.” All patients gave written informed consent in accordance with the Declaration of Helsinki. For patients aged <18 years at the date of first sampling the consent was released by their legal representative.

### Bacterial strains

Ninety-one *S. maltophilia* strains were isolated over a 12 year-period (2003–2014) in sputum samples collected from 10 CF patients (ethically coded as ZC, CV, MS, TG, FMa, AV, GC, MC, BB, and SanG) at the CF Unit of “Bambino Gesù” Children's Hospital of Rome, Italy (Table 1). Only patients chronically infected with *S. maltophilia*, that are those with at least 50% of samples positive in the past 12 months (Pressler et al., 2011), were selected. One strain was collected yearly during a pulmonary exacerbation episode, except when different morphotypes were observed. Strains were identified by Vitek automated system (bioMérieux Italia SpA; Florence, Italy) and stored at −80°C for further characterization. The following control strains were used: *S*. *maltophilia* ATCC13637 (mutation frequency assay), *S. maltophilia* Sm111 knock-out for *fliI-*gene (motility assays) (Pompilio et al., 2010), *Escherichia coli* ATCC 25922, *P. aeruginosa* ATCC 27853 (antibiotic susceptibility tests), and *S. maltophilia* K279a (identification of polymorphisms).

**Table 1:** *Stenotrophomonas maltophilia* strains tested in the present study.

**ID**	**Birth date**	**Number (ST) of strains isolated in the year:**												**#isolates**	**#ST**	**Age^a^**
		**2003**	**2004**	**2005**	**2006**	**2007**	**2008**	**2009**	**2010**	**2011**	**2012**	**2013**	**2014**			
ZC	26/12/1988	–	1(178)	1(5)	4 sequential isolates^b^ (179)				2 sequential isolates (5)		2(5, 179)	2(5, 179)	1(179)	13	3	16
CV	09/09/1988	2(5, 180)	–	5 sequential isolates (5)				–	1(5)	–	–	1(29)		9	3	15
MS	11/05/1992	2(181)	–	–	1(5)	–	1(182)	1(183)	3 sequential isolates (4)			1(92)	1(5)	10	6	11
TG	03/12/1980	–	1(91)	9 sequential isolates (184)					–	–	5 sequential isolates (184)			15	2	24
FMa	20/12/2001	–	–	2(185)	–	1 (2)	6 sequential isolates (185)							9	2	4
AV	28/06/1995	–	–	1(2)	1(2)	–	–	1(183)	1(2)	4 sequential isolates (5)				8	3	10
GC	07/07/2005				–	–	2(91)	1(162)	8 sequential isolates (91)					11	2	3
MC	27/02/2009							1(5)	2(183, 184)	2(186, 187)	3 sequential isolates (186)			8	5	0
BB	13/01/2010								1(139)	2 sequential isolates (188)		1(139)		4	2	0
SanG	09/02/2010								2 sequential isolates (188)		1(5)	1(84)		4	3	0
# isolates		4	2	6	7	5	8	8	11	9	13	11	7			
# ST (first detected)		5, 180, 181	178, 91	184, 185, 2	179		182	183, 162	4, 139, 188	186, 187		84, 29, 92				
# ST (cumulative)		3	5	8	9	9	10	12	15	17	17	20	20			

*Ten chronically infected CF patients were enrolled. S. maltophilia infection was defined as “chronic” if at least 50% of samples taken during 1 year were positive for the microorganism (Pressler et al., 2011). One isolate was collected yearly during a pulmonary exacerbation episode. Multiple isolates were considered only when different morphotypes were observed*.

a *At first sampling*.

b *Sequential isolates are monophyletic, belonging to the same ST (indicated in brackets)*.

### DNA extraction and sequencing library preparation

*S. maltophilia* isolates were grown on Columbia agar plates with 5% sheep blood (bioMérieux Italia S.p.A, Florence, Italy) for 24 h at 37°C, and genomic DNA was extracted using DNeasy Blood & Tissue Kit (Qiagen, Hilden, Germany). DNA was quantified using Qubit 2.0 fluorometer (Invitrogen by Thermo Fisher Scientific, Life Technologies Italia, Monza, Italy). Paired-end libraries were prepared from 1 ng of total bacterial DNA using Nextera XT DNA Sample Preparation kit and Nextera XT Index kit (Illumina Inc., San Diego, California, U.S.A.). Library concentration and average fragment size were calculated by Qubit 2.0 fluorometer and Caliper LabChip GXI System (PerkinElmer, Waltham, USA), respectively. Libraries' concentration was normalized to 2 nM, pooled for multiplexing in equal volumes, and sequenced at 14 pM on the Illumina MiSeq platform (Illumina Inc., San Diego, California, U.S.A.) with 300 nt paired-end reads to achieve a coverage of about 30x per base, using MiSeq V3 flow cell.

### Bioinformatic analyses

Raw reads underwent a series of steps for quality filtering which included a general quality check with the software FastQC (Andrews, 2010) and the trimming of low-quality ends. After the preliminary quality check, library 44 was removed due to its low quality. Library number 21 was removed *a posteriori* due to an anomalous placement in the phylogenetic tree, a blast search on the whole NCBI-nt database showed high similarity of the contigs obtained by this library with *Serratia marcescens*. The de novo assembly was performed using SPAdes (Bankevich et al., 2012). The parameters were the following: the values of “k” set as 23, 43, 63, 83, 103, 113, the cutoff of coverage as 3, and the “-careful” option. Contigs below 200 bp were removed as contigs below this length are not accepted by NCBI whole genome submission system. To improve further the quality of the assembly, raw reads were mapped on the contigs using bowtie2 (Langmead and Salzberg, 2012), and the contigs with no reads mapping were removed. The degree of similarity among genomes was estimated calculating the pairwise average nucleotide identity (ANI), that consists of a comparison between each genome and all the others using BLAST and the averaging of the identity percentages among all hits in each comparison. ANI was calculated using the python package pyani (Pritchard et al., 2016). *In silico* Multi-Locus Sequence Typing (MLST) analysis was done using the specific tool available from the web server of the Centre for Genomic Epidemiology (Larsen et al., 2012). To estimate the relatedness of Sequence Types (STs), an eBURST analysis was performed with PHYLOViZ 2.0 (Nascimento et al., 2016). Polymorphic sites were identified with Snippy (https://github.com/tseemann/snippy), using *S. maltophilia* K279a (NCBI acc. no. NC_010943) as reference strain. Phylogenetic analyses were performed from core Single-Nucleotide Polymorphisms (SNPs) data with the maximum likelihood method as implemented in PHYLIP (Felsenstein, 1989). Phenotypic traits were mapped on the tree using iTOL (Letunic and Bork, 2016). Contigs were annotated using Prokka (Seemann, 2014) and pangenome analysis was done with Roary (Page et al., 2015), using all default parameters for gene clustering. To estimate whether *S. maltophilia* pangenome was open or closed based on Heaps' law model (Tettelin et al., 2008), the alpha decay parameter was calculated using the R-package micropan (Snipen and Liland, 2015). Binary heatmaps for gene (or SNP) presence absence were done in R environment (R Core Team, 2012), using the heatmap() function of the base package. An heatmap representing the phylogenetic distances among isolates was done using the R-package “pheatmap” on a distance matrix calculated on the aligned core SNP-ome with the dist.alignment() function of the “seqinr” R-package. To identify clinically relevant functions, *S. maltophilia* draft genomes were mined for 19 antibiotic resistance genes and for 24 virulence genes described in the literature using BLAST (Altschul et al., 1990), with both coverage and similarity threshold set at 80%. Two additional analyses were performed to maximize the number of resistance genes identified: first, genomes were mined for additional antibiotic resistance genes not specific for *S. maltophilia* using ResFinder tool from the server of the Centre for Genomic Epidemiology (Zankari et al., 2012), setting an identity threshold at 98% and a length threshold at 100%; second, the Resistance Gene Identifier (RGI) software, available from the CARD database (Jia et al., 2017), was run using the “perfect and strict hits only” default parameters. The degree of recombination among the strains was evaluated using the software ClonalFrameML (Didelot and Wilson, 2015), using as inputs the alignment of the core genes obtained by roary.

### Phenotypic characterization

Each *S. maltophilia* strain was evaluated for growth rate, biofilm formation, motility (swimming, swarming, twitching), mutation frequency, *in vivo* virulence, and *in vitro* antibiotic susceptibility, as previously described (Pompilio et al., 2016). All experiments were performed in triplicate and repeated twice.

#### Growth rate

Overnight cultures in Trypticase Soy broth (TSB; Oxoid SpA; Garbagnate M.se, Milan, Italy) were corrected with fresh TSB to an OD_550_ of 1.00, corresponding to about 1–5 × 10^9^ CFU/ml. This suspension was diluted 1:100 in fresh TSB, then 200 μl were dispensed in each well of a microtiter plate (Kartell SpA; Noviglio, Milan, Italy), and incubated at 37°C, under static conditions, in a microplate reader (Sinergy H1 Multi-Mode Reader; BioTek Instruments, Inc., Winooski, VT, USA). OD_550_ readings were taken every 30 min for 24 h. Considering the exponential growth phase selected on a graph of ln OD_550_ vs. time (*t*), mean generation time (MGT) was calculated as follows: MGT = ln2/μ, where μ (growth rate) = (lnOD_*t*_ − lnOD_*t*0_)/*t*.

#### Biofilm formation

Biofilm formation was measured, as previously described (Pompilio et al., 2008), in a flat bottom 96-well polystyrene tissue culture-treated plate (Falcon BD; Milan, Italy). Following 24 h of static incubation at 37°C, biofilm was stained with crystal violet and biomass measured as OD_492_. To calculate the category of biofilm production, we determined the cut-off optical density (ODc) as three standard deviations above the mean OD of the negative control. Tested strains were then divided into four groups, according to Stepanović et al. (2007): OD ≤ ODc (non-adherent strain, no biofilm producer); ODc < OD ≤ 2 × ODc (weak biofilm producer); 2 × ODc < OD ≤ 4 × ODc (moderate biofilm producer); 4 × ODc < OD (strong biofilm producer).

#### Motility

Motility assays were performed as described by Rashid and Kornberg (2000), with modifications. A single colony was inoculated onto swimming (10 g/l tryptone, 5 g/l NaCl, 3 g/l agar) or into swarming (8 g/l nutrient broth, 5 g/l dextrose, g/l agar) agar. After incubation at 37°C for 24 h, swimming and swarming motilities were measured as the diameter of growth zone. Twitching was measured by inoculating a single colony to the bottom of Petri dish containing 1% TSB solidified with 1% agar. After 72 h of incubation at 37°C, the agarose layer was removed and the diameter of twitch zones were measured, following staining with crystal violet solution.

#### Mutation frequency

Mutation frequency was assessed according to Oliver et al. (2000), with modifications. Twenty milliliters of overnight cultures grew in Mueller-Hinton broth (MHB; Oxoid) were centrifuged (4,500 rpm, 10 min, 4°C) and resuspended in 1 ml of MHB. Ten-fold dilution of each sample was seeded onto Mueller-Hinton agar (MHA; Oxoid) plates (controls) and onto MHA + rifampin 250 μg/ml (Sigma-Aldrich) then incubated at 37°C. Colony counts were performed after 24 h- (control plates) or 48 h-incubation (rifampin added plates), and mutation frequency (*f*) was calculated as the ratio of mutants/total cells in the population. Strains were classified as hypo—(*f* ≤ 8 × 10^−9^), normo—(8 × 10^−9^ < *f* < 4 × 10^−8^), weak—(4 × 10^−8^ ≤ *f* < 4 × 10^−7^), and strong-mutators (*f* ≥ 4 × 10^−7^; Turrientes et al., 2010).

#### *In vivo* virulence

*Galleria mellonella* virulence assay was performed as previously described (Betts et al., 2014), with modifications. Overnight cultures of each *S. maltophilia* strain grown in TSB were washed and resuspended in PBS. Twenty larvae were inoculated—directly into the hemocoel via the right proleg using Hamilton syringe—with 10 μl containing 10^3^, 10^4^, 10^5^, and 10^6^ CFU, or PBS only (controls). Larvae were incubated in the dark at 37°C and the number of dead caterpillars was scored every 24 h until 96 h, considering as dead those nonresponsive to touch. A “pathogenicity score” was assigned to each strain, considering both time and dose needed to achieve LD_50_ and LD_100_.

#### Antibiotic susceptibility

*In vitro* susceptibility to doxicycline, trimethoprim-sulfamethoxazole, minocycline, ciprofloxacin, levofloxacin, ticarcillin-clavulanate, ceftazidime, piperacillin-tazobactam, amikacin, and chloramphenicol was assessed by MIC-Test Strip (Liofilchem; Roseto degli Abruzzi, Italy). When CLSI breakpoints were not available for *S. maltophilia* (CLSI, 2016), ECOFF (epidemiological cut-off) values were calculated according EUCAST guidelines (http://www.eucast.org/mic_distributions_and_ecoffs/): 128/4 μg/ml (piperacillin/tazobactam), 16 μg/ml (ciprofloxacin), 64 μg/ml (amikacin).

### Statistical analysis and interpretative criteria

Statistical analysis was performed using Prism 6 (version 6.01; GraphPad Software Inc., La Jolla, USA), considering *p*-values lower than 0.05 as significant. Gaussian distribution was evaluated by D'Agostino-Pearson omnibus and Shapiro-Wilk normality tests. Differences between groups were evaluated using both parametric (one-way ANOVA-test followed by Newman-Keuls' multiple comparison post-test), and non-parametric (Mann-Whitney test; Kruskal-Wallis ANOVA test followed by Dunn's multiple comparison post-test) tests. Differences between proportions were assessed by chi-square test. Correlation between each phenotype and the phylogenetic structure of the population was assessed by Pagel's lambda (Pagel, 1999), using the phylosig() function of the R-package phytools.

To identify temporal trends and correlation among phenotypes, only the monophyletic groups with the highest number of strains colonizing a single patient were investigated in order to strengthen the statistical significance, yet minimizing confounding variables connected to the genetic background of the strain itself and of the host. By this way, five groups of strains were selected: ST184 from patient TG (*n* = 14, TG_184); ST91 from patient GC (*n* = 10, GC_91); ST185 from patient FMa (*n* = 8, FMa_185); ST179 from patient ZC (*n* = 7, ZC_179), and ST5 from patient CV (*n* = 7, CV_5). The two groups with the highest number of strains (TG_184 and GC_91) were selected for a microevolutionary analysis based on a reduced dataset consisting of SNPs that were occurring only in the group under study.

Correlations among pairs of phenotypic traits in each group, as well as the trend of each phenotype over time, were inferred by linear regression analysis. With regard to antibiotic susceptibility tests, out-of-range MICs ≥256 and ≥ 32 μg/ml were considered as 256 and 32 μg/ml, respectively. The graphics displaying the temporal trends in the ST and patient_ST combination were obtained using the “ggplot2” package, the confidence interval was calculated using the function geom_smooth() with the default parameters.

## Results

### Bioinformatic analyses

Table 2 and Supplementary Table 1 report the statistics regarding assembly, SNP analysis, annotation, and phenotypes. The values of assembly statistics and annotation are comparable with those found in other studies (Youenou et al., 2015; Yu et al., 2016). Genome sequences are publicly available under GenBank accession numbers MQWK00000000-MQZW00000000; unassembled reads that passed the cleaning step are available from the Sequence Reads Archive (SRA) at the acc. no. SRR5569163–SRR5569253. ANI values ranged from 0.90 to 1 (with 1 representing perfect id), with an average of 0.95 ± 0.03, calculated over an alignment length ranging from 3.450 to 5.214 kbp, with an average of 3.996 ± 330 kbp (Supplementary Table 2).

**Table 2:** Main assembly, SNP, and annotation statistics.

	**Assembly statistics**							**Mutations**							**Annotation statistics**			
	**#Read pairs**	**Avg. cov**	**Contigs no**.	**N50**	**% Reads mapping**	**GC %**	**Tot. length**	**COMP^a^**	**DEL^b^**		**INS^c^**	**MNP^d^**	**SNP^e^**	**Total**	**genes**	**CDS^f^**	**tRNA**	**rRNA**
Min	299841	21.4	63	45004	95.4	66.0	4340930	3925	132	141		792	18534	23524	3972	3864	70	6
Avg	477042	34.1	105	93888	97.1	66.4	4662510	15193	338	311		2687	57867	76396	4330	4217	74	6
Median	463220	33.1	100	94682	97.1	66.4	4735262	7028	261	250		1348	38944	47777	4403	4290	74	6
Max	759185	54.2	181	143022	98.2	66.8	5214241	42115	753	609		7110	115498	166085	4881	4755	82	11
																		

*Data for each isolate is reported in Supplementary Table 1*.

a *Complex mutations (for instance rearrangements)*.

b *Deletions (with respect to S. maltophilia K279a reference strain)*.

c *Insertions*.

d *Multiple Nucleotide Polymorphisms*.

e *Single Nucleotide Polymorphisms*.

f *Coding Sequences*.

**Figure 1:**
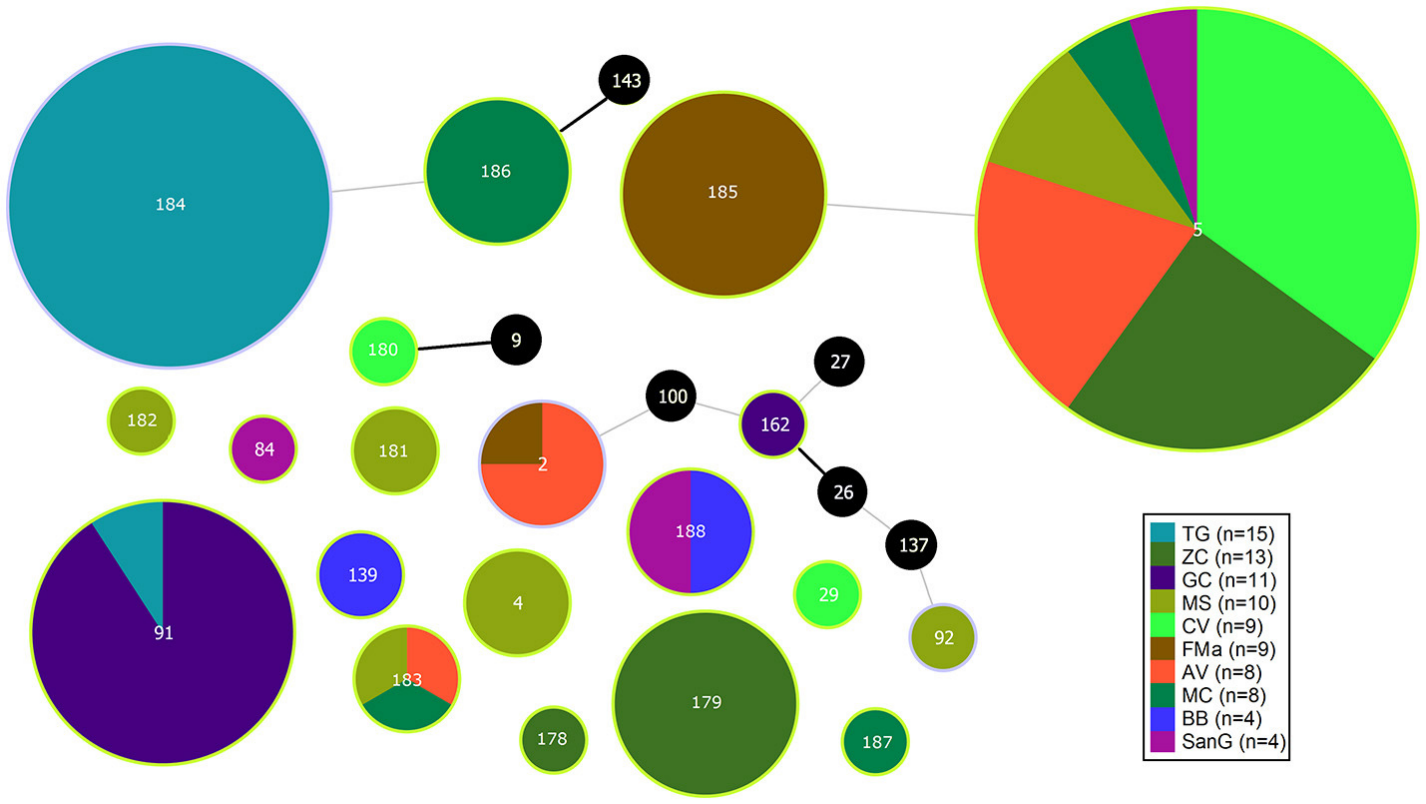
eBURST diagram of *S. maltophilia* population. Each circle represents one ST. Diameter of circles is proportional to the number of isolates belonging to each ST. The thickness and color of the connections between STs represent the number of loci with one or more different allele (thick black link, 1 different allele; thin gray link, 2 different alleles). Black circles represent previously characterized STs which were not found in the present study.

The population consisted of 20 different STs, 11 of which are new ones, including 33 new alleles and 47 strains out of 91 (51.6%); the new STs were submitted to pubMLST.org (Table 3). The eBURST analysis showed that eight of the STs found in this study were linked either each other or with known STs (Figure 1), while the remaining ones showed no first or second level connection to any described ST. Nine out of eleven new STs were found in a single patient whereas this was the case for six out of nine known STs. The most prevalent ST in the population was ST5 (20 isolates from 6 patients), followed by the new ST184 (14 isolates from a single patient), and ST91 (11 isolates from 2 patients). Among the new STs, ST183 was found in the largest number of patients (3) (Figure 1). Table 1 reports the timeline of isolation of STs from each patient. In some cases (patient TG), intermittent infection of the same clone was observed. In some other cases, there are either different clones co-infecting the same patient (as in patient ZC during years 2012–2013), or ST replacement through time was observed (as for patient AV, that was initially infected by ST2 and successively by ST5). Furthermore, in two patients (GC and FMa), the ST found in the initial isolation was no more isolated for 1 or 2 years, and appeared again in a persistent way.

**Table 3:** MLST table resuming allele number of each ST.

**#Unique ST**	***atpD***	***gapA***	***guaA***	***mutM***	***nuoD***	***ppsA***	***recA***	**ST**	**Counts**
1	1	1	18	6	1	4	6	**ST-178**	1
2	5	22	9	4	27	5	7	ST-5	20
3	1	**96**	105	4	28	**108**	62	**ST-179**	7
4	22	26	20	9	4	14	2	**ST-180**	1
5	2	2	5	2	2	3	5	ST-29	1
6	1	**96**	**125**	**82**	6	**109**	7	**ST-181**	2
7	**89**	**95**	**124**	**81**	**90**	**107**	**87**	**ST-182**	1
8	**90**	**97**	**129**	**84**	**91**	**112**	**88**	**ST-183**	3
9	1	4	7	7	28	19	6	ST-4	3
10	1	1	82	3	1	1	1	ST-92	1
11	3	73	89	3	25	80	7	ST-91	11
12	**91**	**98**	**126**	**83**	**92**	**110**	**89**	**ST-184**	14
13	5	22	9	75	27	80	7	**ST-185**	8
14	1	1	12	3	28	7	1	ST-2	4
15	1	1	4	3	6	4	1	ST-162	1
16	13	**99**	**127**	**83**	**89**	**111**	80	**ST-186**	5
17	**92**	4	**128**	57	25	20	**90**	**ST-188**	1
18	3	4	110	46	6	38	58	ST-139	2
19	1	4	18	3	28	80	72	**ST-187**	4
20	3	1	82	3	25	4	62	ST-84	1

*In bold, the new ones found in this study*.

**Figure 2:**
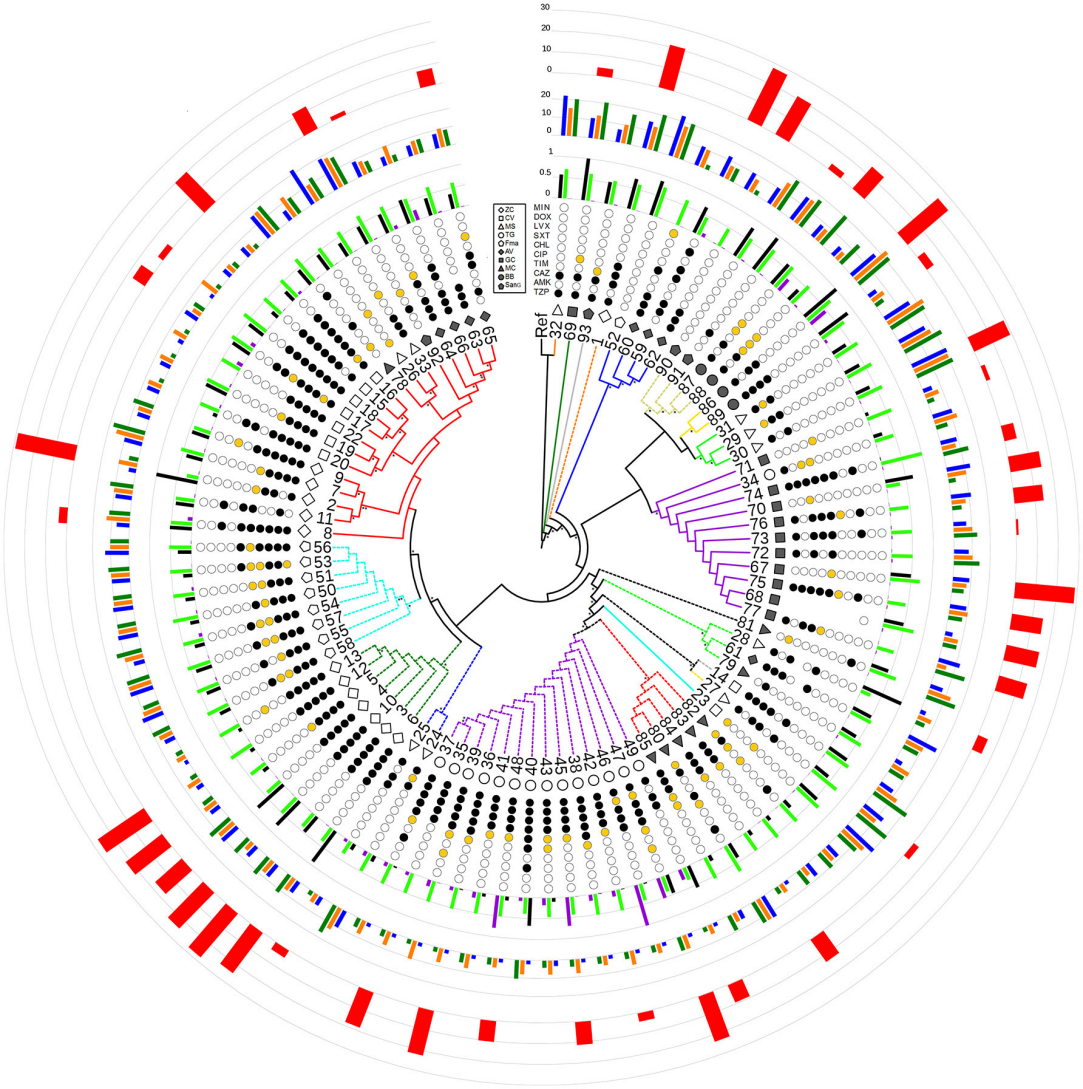
Radial cladogram inferred from a Maximum Likelihood analysis of core genome SNPs and including all phenotypic traits. Branches are colored according to ST, and dashed branches represent new STs. Innermost to outermost circles represent: (i) patient IDs (ZC, empty diamond; CV, empty square; MS, empty triangle; TG, empty circle; FMa, empty pentagon; AV, full diamond; GC, full square; MC, full triangle; BB, full circle; SanG, full pentagon); (ii) antibiotic susceptibility profiles (codified as: white-susceptible, orange-intermediate, and black-resistant) for the following antibiotics: TZP, piperacillin/tazobactam; AMK, amikacin; CAZ, ceftazidime; TIM, ticarcillin/clavulanic acid; CIP, ciprofloxacin; CHL, chloramphenicol; SXT, trimethoprim/sulfamethoxazole; LVX, levofloxacin; DOX, doxicycline; and MIN, minocycline; (iii) biofilm biomass (black), growth rate (green), and mutation frequency (purple), normalized dividing each value by the maximum value (the out-of-range biofilm values of strains 2, 28, and 69 were reported as 1 for visualization issues); (iv) swimming (blue), swarming (orange), and twitching motility (green); (v) *G. mellonella* virulence scores. Bootstrap support is codified as “^*^” for >95% and “^**^” for 75–95%, placed at the nodes of the tree.

**Figure 3:**
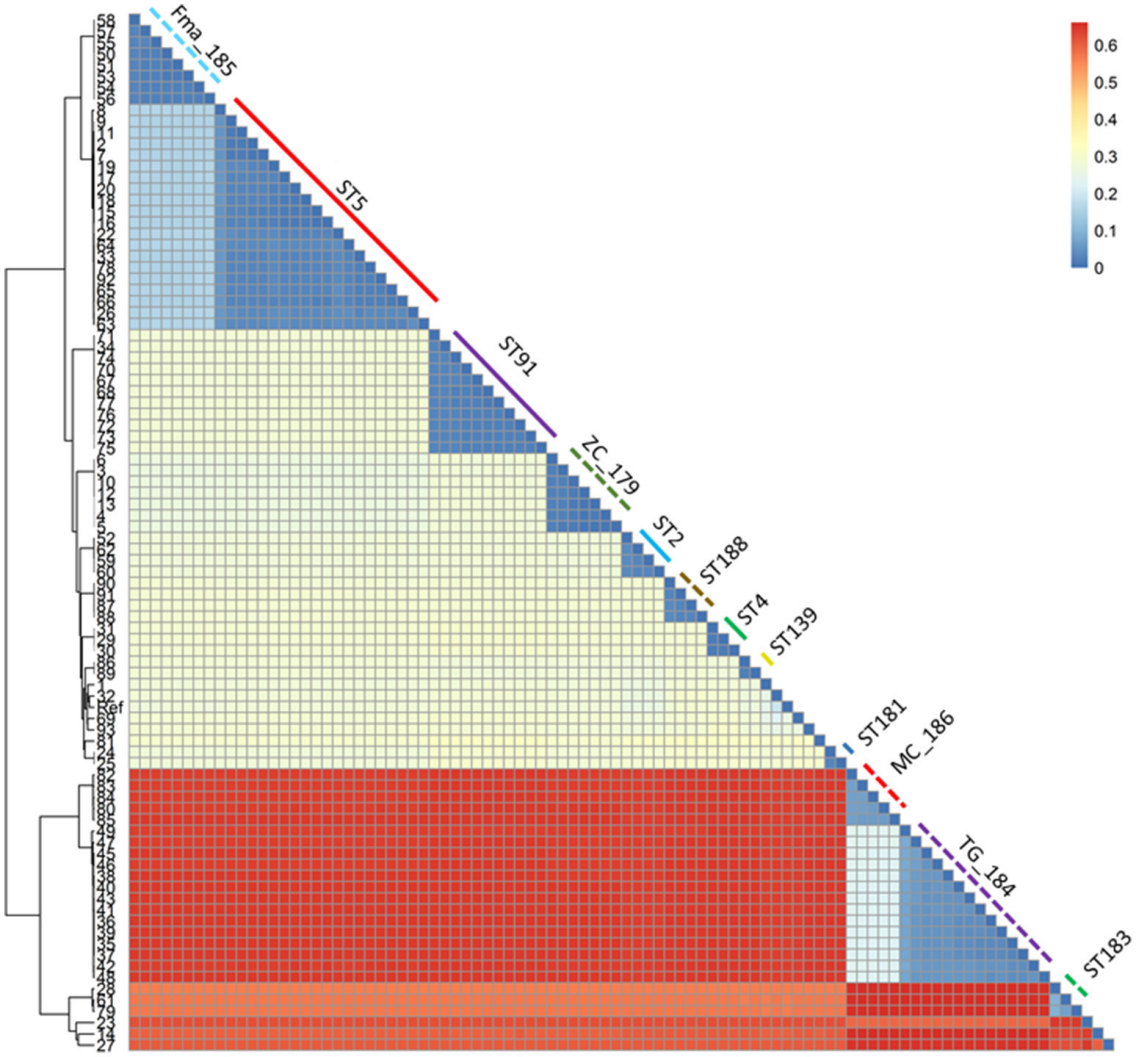
Heatmap representing the phylogenetic distances as inferred by the SNP data. The clustering pattern reflects the one in Figure 2, but the distance between the two main clusters and the minor ones is highlighted. The monophyletic STs are shown with the same color and line type as in Figure 2.

**Figure 4:**
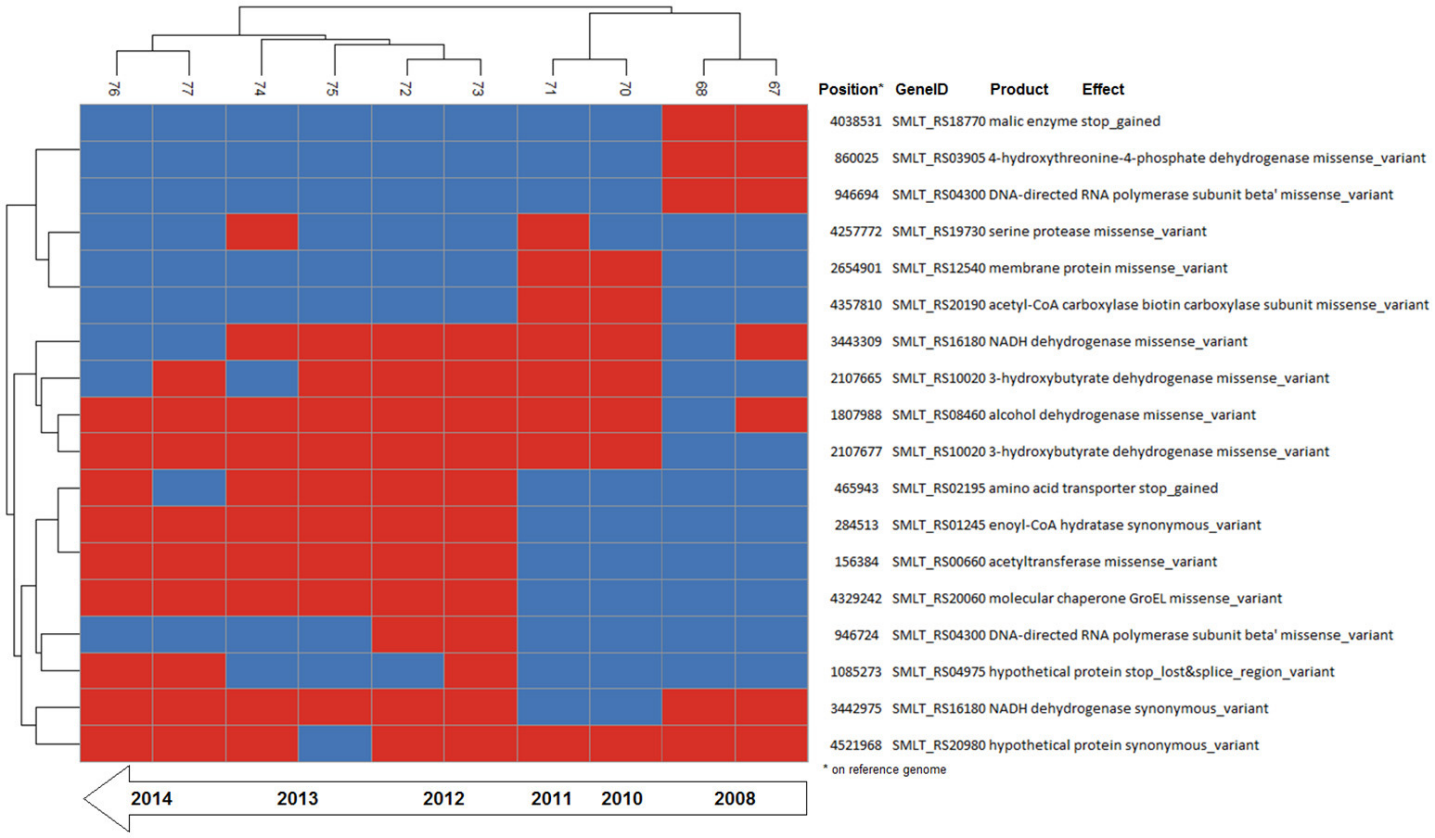
Binary heatmap of the subset of SNP data present only in isolates of patient_ST combination GC_91. Annotations for each SNP are shown on the right panel.

A total of 279,538 SNPs was identified. The phylogenetic tree based on the alignment of all polymorphic positions consisted of two major lineages separated by a large phylogenetic distance (Figures 2, 3). One cluster included the reference strain with most strains belonging to known STs, while the other one comprised all isolates from TG_184 and MC_186; another minor monophyletic lineage was also present (ST183, Figure 3). The number of SNPs unique to TG_184 and GC_91 were 2,940 and 1,109, respectively. All SNPs were monomorphic and the number of variable SNPs within the group was 783 (in TG_184) and 18 (in GC_91). A cluster analysis based on the reduced datasets was performed for both TG_184 and GC_91, revealing that only in the latter case the clustering pattern was consistent with the timeline of isolation (Figure 4, Supplementary Figure 1). This set of isolates was considered as likely deriving from a single recent common ancestor. A closer look was given on the kind of mutations (missense, nonsense, silent) occurring in this series (Figure 4). Seven genes with fixed mutations were identified during the course of infection, namely: SMLT_RS18770, SMLT_RS03905, SMLT_RS04300, SMLT_RS10020, SMLT_RS01245, SMLT_RS00660, and SMLT_RS20060 (Figure 4). The pangenome consisted of 16,486 genes, with a core genome of 1,911 genes (Supplementary Figure 2). The number of genes present in a single genome (strain-specific genes) was 5,599 (33.9% of the whole gene pool). According to Heaps' law, the pangenome was open (alpha = 0.77). The phylogenomic dendrogram based on gene presence/absence was globally consistent with the topology and branch lengths of the SNPs-based tree (Supplementary Figure 3). *S. maltophilia*-specific genes for antibiotic resistance (identified using BLAST) were ubiquitous, with the exception of *qnrR* (found in 89 isolates), *smeO* and *smeY* (90 isolates), and *spgM* (66 isolates). ResFinder analysis instead, which looks at genes nonspecific to *S. maltophilia*, found very few genes in very few strains, except for *aac(6*′*)-Iz* and *aph(3*′*)-IIc* which were found in 50 and 42 strains, respectively (Supplementary Figure 4A). A widely overlapping result was found by the RGI software of CARD database, with 46 genes (among which 24 ubiquitary ones) belonging to 10 different ARO categories (Supplementary Table 3). Similarly, virulence genes were either widely distributed or rarely found (Supplementary Figure 4B). We acknowledge that in some instances (for example for *smeO, smeI*, and *qnrR*), the absence of the gene in a single isolate may be due to the fragmentation of the assembly. The clustering pattern defined by ClonalFrameML was highly consistent with the one shown in Figure 2. The recombination events within the clusters seem to be not homogeneous neither among STs nor among patient_ST combinations (Supplementary Figure 5). An additional analysis was performed on the alignment of nucleotide sequences of genes known to be involved in the mutator phenotype (*mutL, mutS*, and *uvrD*). Main statistics about polymorphic sites and neutral evolution test are reported in Supplementary Table 4.

### Phenotypic trends over time

All traits showed significant temporal trends or significantly different values among STs, patient or both (see Supplementary Figures 6A–G).

#### Biofilm formation

ST5 isolates formed significantly higher biofilm biomass amount than those belonging to ST184 (OD_492_ median values: 0.369 vs. 0.029, respectively; *p* < 0.05; Supplementary Figure 6A2). A significantly higher percentage of “strong producers” strains was observed in ST5 compared to ST184 (35 vs. 0%; *p* < 0.05), whereas “non-producers” strains were found mostly in ST184 (60 vs. 5%, respectively; *p* < 0.01; Supplementary Table 5). ST91 isolates significantly decreased their ability to form biofilm over time in patient GC (*r*^2^ = 0.697; *p* < 0.01; Table 4; Supplementary Figure 6A4).

**Table 4:** *Stenotrophomonas maltophilia* trends in expression over time across all phenotypic traits, both in the most prevalent sequence types (STs) and ST_patient combinations.

**ST^a^ or ST_patient (n)**	**Traits**							
	**Growth rate**	**Biofilm formation**	**Mutation frequency**	**Swimming motility**	**Twitching motility**	**Swarming motility**	***In vivo* virulence**	**Antibiotic resistance^b^**
**ST5 (20)**	0.042 (0.384)	0.066 (0.273)	0.05 (0.342)	**0.248 (0.025)**	**0.243 (0.027)**	0.128 (0.121)	0.127 (0.122)	0.094 (0.186)
**ST91 (11)**	0.003 (0.859)	0.094 (0.357)	0.241 (0.124)	0.01 (0.766)	0.196 (0.172)	0.266 (0.104)	0.252 (0.115)	0.308 (0.095)
**ST179 (7)**	**0.768 (0.009)**	0.171 (0.355)	0.153 (0.384)	**0.648 (0.028)**	0.544 (0.058)	0.497 (0.076)	0.21 (0.3)	0.340 (0.169)
**ST184 (15)**	0.193 (0.1)	0.153 (0.149)	**0.502 (0.003)**	0.003 (0.835)	0.002 (0.846)	0.1 (0.25)	0.018 (0.625)	0.204 (0.09)
**ST185 (8)**	0.003 (0.892)	0.484 (0.055)	0.12 (0.4)	0.036 (0.649)	0.018 (0.746)	0.01 (0.809)	ND^c^	0.438 (0.073)
**CV_5 (7)**	0.142 (0.404)	0.116 (0.454)	0.32 (0.185)	0.074 (0.553)	0.096 (0.496)	0.074 (0.553)	0.11 (0.466)	0.347 (0.163)
**GC_91 (10)**	0.074 (0.446)	**0.697 (0.002)**	0.014 (0.737)	0.176 (0.226)	0.21 (0.182)	0.012 (0.76)	0.185 (0.214)	**0.607 (0.013)**
**ZC_179 (7)**	**0.768 (0.009)**	0.171 (0.355)	0.153 (0.384)	**0.648 (0.028)**	0.544 (0.058)	0.497 (0.076)	0.21 (0.3)	0.340 (0.169)
**TG_184 (14)**	**0.462 (0.007)**	0.151 (0.168)	**0.564 (0.001)**	0.063 (0.386)	0 (0.959)	**0.315 (0.036)**	0.023 (0.599)	0.213 (0.096)
**FMa_185 (8)**	0.003 (0.892)	0.484 (0.055)	0.12 (0.4)	0.036 (0.649)	0.018 (0.746)	0.01 (0.809)	ND^c^	0.438 (0.073)

*The statistical significance of a trend was assessed by linear regression analysis. r^2^ values, along with associated two-tailed p-values (in brackets), are shown. Significant (p < 0.05) values are in bold: red (increasing trend) or blue (decreasing trend)*.

a *ST, sequence type*.

b *Considered as the number of intermediate/resistant isolates*.

c *ND, not determined: virulence score of ST185 isolates was always null*.

#### Growth rate

ST5 isolates showed significantly higher growth rate than ST184 ones (U/h, median values: 0.444 vs. 0.386, respectively; *p* < 0.05; Supplementary Figure 6B2). An opposite trend was found according to the patient_ST combination considered: positive for ZC_179 (*r*^2^ = 0.768; *p* < 0.001), negative for TG_184 (*r*^2^ = −0.462; *p* < 0.01; Table 4; Supplementary Figure 6B4).

#### Motility

ST184 isolates exhibited lower swimming motility compared to ST185, ST5, and ST91 ones (median value: 2 vs. 11, 7, and 9 mm, respectively; *p* < 0.01; Supplementary Figure 6C2), and also decreased swarming and twitching motilities compared with ST185 isolates (swarming: 3 vs. 4 mm; twitching: 3 vs. 8 mm, respectively; *p* < 0.05; Supplementary Figures 6D2,E2). Trends in motility over time differed according to ST and motility type considered. ST5 isolates significantly increased swimming and twitching motility over time (*r*^2^ = 0.248 and 0.243, respectively; *p* < 0.05; Table 4; Supplementary Figures 6C3,E3). Contrarily, a decreasing trend was observed for both swarming in TG_184 isolates (*r*^2^ = 0.315; *p* < 0.05), and swimming in ZC_179 isolates (*r*^2^ = 0.648; *p* < 0.05; Table 4; Supplementary Figures 6C4,D4).

#### Mutation frequency

Most of strains were weak-mutators (45 out of 91, 49.4%), with a frequency significantly higher (*p* ≤ 0.01) than that observed for strong—(28, 30.8%), normo—(16, 17.6%), and hypo-mutators (2, 2.2%). ST184 isolates showed the highest median mutation frequency (1.3 × 10^−6^ vs. 1.4 × 10^−7^, 1.2 × 10^−7^, 2.9 × 10^−8^, and 1.3 × 10^−7^ for ST5, ST91, ST179, and ST185, respectively; *p* < 0.05; Supplementary Figure 6F-2). The occurrence of strong-mutators in ST184 (93.4%) was in fact significantly higher compared to other STs (30, 25, and 0% for ST5, ST185, ST91, and ST179, respectively; *p* < 0.001; Supplementary Table 6). TG_184 isolates showed significant trend toward increased mutation rate (*r*^2^ = 0.564; *p*<0.01; Supplementary Table 6; Supplementary Figure 6F4).

#### *In vivo* virulence

ST179 isolates resulted to be significantly more virulent in *G. mellonella* compared to ST5 (median virulence score: 24 vs. 0, respectively; *p* < 0.001). ST185 isolates were completely avirulent, resulting significantly different from ST179 isolates (*p* < 0.001) and ST91 isolates (median score: 14; *p* < 0.05; Supplementary Figure 6G2). No significant temporal trend was observed for virulence, regardless of ST and ST_patient combination considered (Table 4).

#### Antibiotic susceptibility

Minocycline, doxycycline, and trimethoprim/sulfamethoxazole were the most effective antibiotics against *S. maltophilia*, showing comparable susceptibility rates (98.9, 94.6, and 93.4%, respectively), whereas ceftazidime, piperacillin/tazobactam, and amikacin were the least effective, showing a resistance rate of 86.6, 85.5, and 62%, respectively (Supplementary Table 6). A significant trend toward increased number of resistant strains over time was observed only in GC_91 isolates (*r*^2^ = 0.607; *p* < 0.01; Table 4).

MIC-values were stratified on ST and results are summarized in Supplementary Tables 7, 8. Isolates belonging to ST179 were more resistant to levofloxacin compared with ST184 and ST185 ones (85.7 vs. 26.6% and 0%, respectively; *p* < 0.05), but susceptible to chloramphenicol differently from ST5, ST184, and ST185 isolates (resistance rate: 0 vs. 55%, 53.3%, and 75%, respectively; *p* < 0.05). ST91 isolates were less resistant to amikacin than those belonging to other STs (20 vs. 100%, 93.3, 100, and 80%, for ST179, ST184, ST185, and ST5, respectively; *p* < 0.01). ST5 isolates were less esistant to ticarcillin/clavulanic acid compared to ST179, ST184, and ST185 (55 vs. 100%, 93.3 and 100%, respectively; *p* < 0.05).

Trends in MIC-values differed according to antibiotic, ST, and ST_patient combination (Table 5; Supplementary Figures 7–15). Minocycline MICs only did not significantly change over time, whereas a constant trend was observed for fluoroquinolones (decreasing) and ceftazidime (increasing). ST179 isolates did not significantly change their MICs over time, regardless of antibiotic considered, whereas ST5 ones exhibited significant trends for four antibiotics, mostly decreasing. Only a trend with of increased values was found in ST91 isolates for ticarcillin/clavulanic acid (*r*^2^ = 0.448; *p* < 0.05) and ceftazidime (*r*^2^ = 0.483; *p* < 0.05). Regarding ST_patient combinations, it is worth noting that the persistence of ST91 in patient GC was associated with increased MICs of piperacillin/tazobactam (*r*^2^ = 0.713; *p* < 0.01), ticarcillin/clavulanic acid (*r*^2^ = 0.629; *p* < 0.05), and ceftazidime (*r*^2^ = 0.722; *p* < 0.01).

**Table 5:** *Stenotrophomonas maltophilia* trends in susceptibility (MIC values) to nine antibiotics over time.

**ST^b^ or ST_patient (n)**	**Antibiotics^a^**								
	**TZP**	**LVX**	**AK**	**SXT**	**MIN**	**TIM**	**CHL**	**CIP**	**CAZ**
**ST5 (20)**	0.046 (0.358)	**0.253 (0.023)**	**0.294 (0.013)**	**0.437 (0.001)**	0.082 (0.22)	**0.338 (0.008)**	0.073 (0.248)	0.097 (0.18)	0.013 (0.622)
**ST91 (11)**	0.086 (0.408)	0.199 (0.195)	0.143 (0.28)	0.185 (0.214)	0.187 (0.211)	**0.448 (0.034)**	0.316 (0.09)	0.207 (0.185)	**0.483 (0.025)**
**ST179 (7)**	ND^c^	0.418 (0.116)	ND	0.391 (0.132)	0.271 (0.23)	ND	0.272 (0.229)	0.367 (0.149)	ND
**ST184 (15)**	0.003 (0.828)	0.101 (0.246)	**0.406 (0.01)**	0.004 (0.805)	0.059 (0.382)	0.003 (0.828)	0.028 (0.548)	0.222 (0.075)	0.003 (0.828)
**ST185 (8)**	0.013 (0.783)	0.031 (0.673)	ND	0.263 (0.193)	0.284 (0.173)	0.034 (0.661)	**0.776 (0.003)**	**0.603 (0.023)**	ND
**CV_5 (7)**	ND	0.406 (0.123)	**0.587 (0.044)**	0.404 (0.124)	0.263 (0.238)	ND	**0.722 (0.015)**	0.371 (0.146)	ND
**GC_91 (10)**	**0.713 (0.004)**	0.169 (0.27)	0.089 (0.433)	0.104 (0.395)	0.131 (0.337)	**0.629 (0.01)**	0.2 (0.226)	0.246 (0.174)	**0.722 (0.003)**
**ZC_179 (7)**	ND	0.418 (0.116)	ND	0.391 (0.132)	0.271 (0.23)	ND	0.272 (0.229)	0.367 (0.149)	ND
**TG_184 (14)**	ND	0.103 (0.284)	**0.425 (0.011)**	0.077 (0.336)	0.112 (0.24)	ND	0.027 (0.57)	0.219 (0.09)	ND
**FMa_185 (8)**	0.013 (0.783)	0.031 (0.673)	ND	0.263 (0.193)	0.284 (0.173)	0.034 (0.661)	**0.776 (0.003)**	**0.603 (0.023)**	ND

*The statistical significance of a trend was assessed by linear regression analysis. r^2^ values, along with associated two-tailed p-values (in brackets), are shown. Significant (p < 0.05) values are in bold: red (increasing trend) or blue (decreasing trend)*.

a *TZP, piperacillin/tazobactam; LVX, levofloxacin; AMK, amikacin; SXT, trimethoprim/sulfamethoxazole; MIN, minocycline; TIM, ticarcillin/clavulanic acid; CHL, chloramphenicol; CIP, ciprofloxacin; CAZ, ceftazidime*.

b *ST, sequence type*.

c *ND, not determined: virulence score of ST185 isolates was always null*.

### Correlation among phenotypic traits

Spearman *r*-values measurements (Table 6) showed that: (i) growth rate is positively correlated with all motility types tested, but negatively related to biofilm production; (ii) mutation frequency was inversely correlated with both swimming and twitching motility; and (iii) twitching was positively correlated with both swimming and swarming motility. Several trends were observed when data was stratified according to ST (Supplementary Table 9) or ST_patient combination (Supplementary Table 10), except for ST185 and FMa_185 isolates for which no significant correlations were found. Swimming, twitching, and resistance to amikacin and ciprofloxacin were the only phenotypes showing a low (0.50 > λ > 0.55), but significant (*p* < 0.01), phylogenetic signal (Supplementary Table 11).

**Table 6:** Spearman correlation values (upper half) and *p*-values (lower half) for each pairwise comparison of phenotype values on the whole population (*n* = 91).

	**Biofilm biomass**	**Growth rate**	**Swimming**	**Twitching**	**Swarming**	**Mutation frequency**	**Virulence score (LD_50_+ LD_100_)**
Biofilm biomass		−**0.217**	−0.045	−0.146	−0.183	0.106	0.168
Growth rate	**0.0385**		**0.429**	**0.386**	**0.454**	−**0.229**	0.005
Swimming	0.672	**<0.001**		**0.655**	**0.659**	−**0.359**	−0.044
Twitching	0.167	**<0.001**	**<0.001**		**0.608**	−**0.311**	−0.023
Swarming	0.0812	**<0.001**	**<0.001**	**<0.001**		−0.163	−0.056
Mutation frequency	0.143	**0.028**	**<0.001**	**0.002**	0.126		−0.069
Virulence score (LD_50_ + LD_100_)	0.109	0.958	0.674	0.827	0.597	0.511	

Mutation frequency was associated to antibiotic resistance (data not shown). Considering isolates as a whole, the proportion of MDR strains was significantly higher in strong-mutators compared to weak-mutators (100 vs. 73.3%, respectively; *p* < 0.05). Particularly, the mean number of resistances per isolate was significantly higher in strong- and normo-mutators than weak-mutators (5.4 and 4.3 vs. 3.0, respectively; *p* < 0.0001 and *p* < 0.05, respectively).

Stratifying on antibiotics tested, strong-mutator isolates were found significantly more resistant to ticarcillin/clavulanate and ceftazidime compared to weak-mutators, as shown by both percentage of resistance (ticarcillin/clavulanate: 82.1 vs. 53.3%, respectively; ceftazidime: 100 vs. 77.8%, respectively; *p* < 0.05) and the mean MIC-value of each antibiotic (ticarcillin/clavulanate: 194.9 vs. 95.7, respectively, *p* < 0.01; ceftazidime: 256 vs. 185.8, respectively, *p* < 0.01). The same trend in mean MIC values was observed for piperacillin/tazobactam (247.4 vs. 179.6, respectively; *p* < 0.05).

## Discussion

The CF lung is a very stressful environment for infecting bacterial populations that must overcome these challenges to persist and survive, thus leading to a strong genotypic and phenotypic diversification. After infection, bacteria are exposed to several postulated stressors including mucin hypersecretion and increased osmotic pressure (Henderson et al., 2014), oxidative and nitrosative stresses (Antus, 2016) due to host responses, sublethal concentrations of antibiotics (Wozniak and Keyser, 2004), and the competition with other microorganisms (Pompilio et al., 2015).

However, CF lung is also the source of a wide variety of untapped ecological opportunities, thus originating an explosive radiation of bacterial population toward specialized phenotypes. This is the case for *P. aeruginosa*, whose adaptation to the CF lung is accelerated by the generation of mutant variants, including mutators, that can additionally increase adaptation producing niche specialists (Smith et al., 2006; Waters et al., 2011; Markussen et al., 2014). In fact, higher mutation supply provides more opportunities for adaptation by means of adaptive changes such as, mucoid conversion (Govan and Deretic, 1996), a switch to an non-motile biofilm lifestyle (McElroy et al., 2014), loss of major virulence factors (Bianconi et al., 2015), auxotrophy in the amino acid-rich lung environment (Barth and Pitt, 1996), loss of motility (Mahenthiralingam et al., 1994), and the emergence of hypermutators (Oliver et al., 2000).

Given that CF patients are constantly exposed to repeated and often intensive antibiotic therapy cycles, the evolution of antibiotic resistance is also a common adaptation (Macia et al., 2005). Once chronic infection is established, adapted mutants start new cycles of mutagenesis and selection of further adaptations in a self-sustained vicious circle, which makes the infection virtually impossible to eradicate. Although the genetic adaptations and related phenotypic variations occurring during CF chronic lung infection are well documented in *P. aeruginosa* (Clark et al., 2015; Cullen and McClean, 2015), the mechanisms driving *S. maltophilia* persistence in CF lungs remain largely unknown.

In the present work, we aimed at increase our understanding of the genetic and phenotypic mechanisms underlying *S. maltophilia* persistence in CF lung, looking at the existence of typical genotypic and/or phenotypical profiles related to chronic infection. The approach followed in this study was to combine genotypic profiling with phenotypic characterization to compare 91 *S. maltophilia* isolates recovered, over 12 year-period, from 10 chronically infected CF patients. The high degree of genetic diversity in *S. maltophilia* is well recognized, and it has been proposed that this species actually consists of a complex (Svensson-Stadler et al., 2012). A previous study (Ormerod et al., 2015) found an ANI of 91.5% between two reference genomes (K279a and JV3), while genomes within a given species usually have an ANI >95%. In our dataset we found ANI values as low as 0.90%, therefore our survey corroborates this statement.

Eleven new STs including 33 new alleles were found in the population, and at least two new deeply branching lineages were revealed: a major one constituted by ST184 and ST186 isolates, and a smaller one constituted by ST183 isolates. Despite the fact that all CF patients were colonized by multiple STs (with up to six different STs in patient MS), most STs (15 out of 20) were occurring in a single patient and showed persistence over years in many cases (Figure 1, Table 1). Differently, using DNA fingerprinting by repetitive sequence-based PCR Vidigal et al. (2014) found a unique *S. maltophilia* genotype in 6 out of 19 (31.6%) CF patients during the 4-year study course. This discrepancy might be due both to the lower discriminatory power of rep-PCR compared to MLST that, although more expensive and time consuming, represents along with PFGE the typing method of choice (Gherardi et al., 2015) in the shorter study-period considered in the spanish study.

The monophyletic lineages of the phylogenetic tree were in perfect agreement with the ST, and support their relationships revealed by eBURST analysis. This is the case for instance for the pairs ST5–ST185 and ST184–ST186, which branch as sister groups in the phylogenetic tree and are also connected in the eBURST diagram (Figures 1, 2). The nearly perfect correspondence of the clustering pattern derived from core genome SNPs and pan-genomic analysis of the accessory genome further supports this observation: clusters of isolates belonging to TG_184 and MC_186 combinations have 1,094 genes differing from all others (Supplementary Figure 1). Furthermore, a clear difference of gene presence/absence pattern in isolates 23 (ST29), 14 (ST180), 27 (ST182), and 28, 61, 79 (ST183) was noted. The result of this heterogeneity is a very narrow core genome of only 1,911 genes out of 16,486 genes in the pangenome (11.6%). A possible explanation for this is that *S. maltophilia* lives in a community, and that sympatric species tend to have large genomes and an open pan-genome (Rouli et al., 2015).

The patient_ST combinations TG_184 and GC_91 were those with the highest number of isolates and were thus suitable for a microevolutionary study. The clustering pattern of the combination patient_ST GC_91 was consistent with the time of sampling, with the late isolates branching out from the earlier ones, and 7 out of 18 mutations were fixed over time (Figure 4).

The number of SNPs was about one order of magnitude higher in ST184 isolates (highest in the late isolates 47–49) than in any other isolate (Supplementary Table 1). In this frame, the prevalence of strong-mutators we found (28 out of 91 strains, 30.8%) was comparable to those observed in previous studies (Turrientes et al., 2010; Vidigal et al., 2014). Hypermutator phenotype clusterized mainly in ST184 whose strains were, all but one, strong mutators. In disagreement with Vidigal et al. (2014), we did not observe decreased mutation rate over time. Contrarily, a significant trend toward increased mutation rate in patient TG was observed (Table 4; Supplementary Figure 6F4).

Although *S. maltophilia* chronic infection has recently been shown to be an independent predictor of pulmonary exacerbation requiring hospitalization and antibiotics (Waters et al., 2011, 2013), the role of antibiotic treatment of these infections in CF people is still unclear since randomized clinical trials are lacking (Amin and Waters, 2016). Trimethoprim/sulfamethoxazole, the first-line therapy for *S. maltophilia* infections, and minocycline and doxycycline, that have also shown some promise to assist with the treatment (Farrell et al., 2010; Chung et al., 2013), resulted the most active antibiotics against isolates tested in the present study. Trends in MIC-values, as well as percentage of resistance, differed according to antibiotic, ST, and ST_patient combination. In disagreement with Vidigal et al. (2014), we observed that hypermutation plays a role in the evolution of *S. maltophilia* resistance, at least toward ticarcillin/clavulanate, ceftazidime, and piperacillin/tazobactam. Similarily, *P. aeruginosa* mutators were more frequently resistant to different antibiotics than nonmutators isolates (Oliver et al., 2000).

The analysis of antibiotic resistance and virulence genes showed that the gene repertoire is homogeneous within the population, and no significant association between the antibiotic susceptibility profiles or virulence phenotypes with the presence of those genes was found (Supplementary Figures 4A,B). The lack of genotype-phenotype association has been previously reported for *S. maltophilia*, with specific reference to antibiotic susceptibility profiles (Youenou et al., 2015). It is well known that SNPs accumulated in *P. aeruginosa* populations colonizing CF patients are highly homoplastic, and that phenotypes display significant correlation with recombinational events rather than with SNPs themselves (Darch et al., 2015). The fact that recombination is one of the main driver of genetic diversity in *S. maltophilia* could further explain this discrepancy (Yu et al., 2016). Recombination seems to be widespread within the population (Supplementary Figure 5), although the genomes belonging to the highly mutable ST184 appear to contain more homoplasic characters, implying an higher recombination rate for this ST. Conversely the patient_ST combinations ZC_179, Fma_185, and GC_91 display an higher degree of clonality.

**Figure 5:**
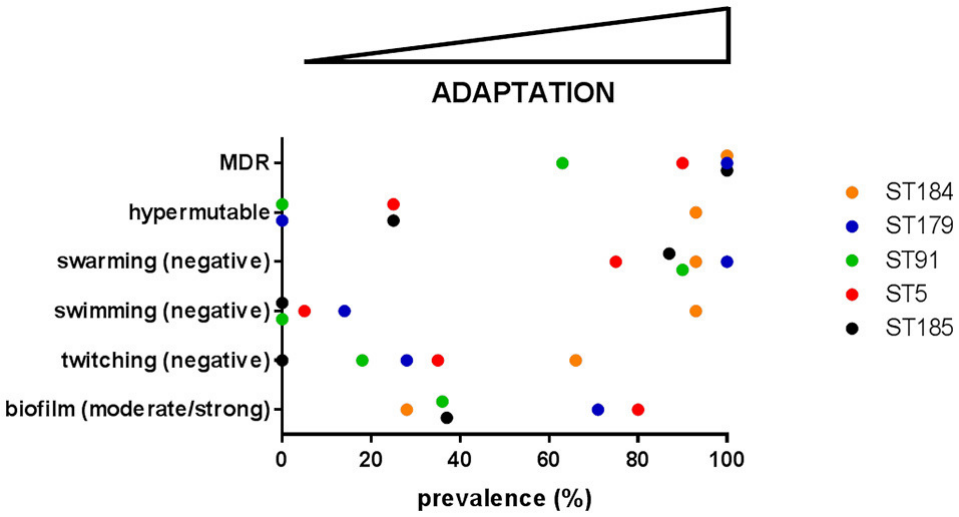
The prevalence of each evolutionary change observed in *P. aeruginosa*—namely transition to a biofilm lifestyle, increased antibiotic resistance, loss of motility, and the emergence of hypermutators (Winstanley et al., 2016)—was calculated for each of the five most abundant *S. maltophilia* ST. On this basis, adaptation level to CF lung during chronic infection increases from left-to-right. MDR: multidrug resistance.

Trend in phenotypic traits changed over time, depending on both patient and ST, therefore suggesting niche separation and the existence of a competition among different genotypes for the same specific niches of CF lung. Particularly, in four cases (growth rate and swarming motility in ST184, biofilm formation and antibiotic resistance in ST91) temporal trends that were not significant for the ST itself resulted to be significant when considering only isolates from a single patient. Conversely, in one case (twitching motility in ST5) a significant trend for the whole ST was not confirmed by the corresponding patient_ST combination, probably due to the low number of isolates for CV_5. Clonal complexes showed different levels of adaptation and persistence in their host, with replacements of infecting ST. ST5 was the most frequent among known STs, thus confirming its successful genetic background (Corlouer et al., 2017). However, according to the main evolutionary changes observed in *P. aeruginosa* during CF lung chronic infection (transition to a biofilm lifestyle, increased antibiotic resistance, loss of motility, and the emergence of hypermutators; Winstanley et al., 2016), *S*. *maltophilia* isolates belonging to the new ST184 showed the highest level of adaptation to CF lung (Figure 5).

The main conclusion arising from our results is that during chronic infection in CF patients *S. maltophilia* populations exhibit consistent genotypic and phenotypic heterogeneity. Probably, this is due to the “biological cost” the microorganism has to pay to successfully adapt to the highly stressful CF lung environment, and in the presence of different selection pressures depending on the host environment. In confirmation of this, the findings from the present study could not confirm trends we observed in a preliminary study aimed at investigating the adaptation of *S. maltophilia* to the lung of a single CF patient (Pompilio et al., 2016). The high genomic heterogeneity results in a wide range of phenotypes, which are only marginally correlated with the distribution of mutations across the genomes. This poor genotype-phenotype correlation is probably a result of the inherent complexity of *S. maltophilia* regulatory networks, whose mechanisms need to be explored in order to design better strategies for clinical intervention.

## Author contributions

AE, AP, OJ, and GDB contributed equally to this report. GDB and OJ jointly conceived the study. AP, VC, CB, and EG performed the experiments. AE and AP analyzed genomic and phenotypic data. AE, GDB, AP, and OJ analyzed data, interpreted results, and wrote the manuscript. EF interpreted results. All authors have read and approved the final version of the manuscript.

### Conflict of interest statement

The authors declare that the research was conducted in the absence of any commercial or financial relationships that could be construed as a potential conflict of interest.
