# Supplementary material for: Evolution of Stenotrophomonas maltophilia in Cystic Fibrosis Lung over Chronic Infection: A Genomic and Phenotypic Population Study
Source: Front Microbiol. 2017 Aug 28;8:1590. doi: 10.3389/fmicb.2017.01590 (PMC5581383; doi:10.3389/fmicb.2017.01590)
Supplement: Supplementary file 1 [file Table1.PDF]

Supplementary Table 1. Extended dataset including all data from Table 2 (divided by library) and data regarding the S. maltophilia phenotypes measured.

Library	Assembly statistics						
	# read pairs	Avg. Cov.	contigs no.	N50 (bp)	% reads mapping	GC %	Tot. Length (bp)
1	410.354	29,31	123	74.976	97,7	66,58	4.560.217
2	585.490	41,82	96	105.706	97,6	66,26	4.890.779
3	409.628	29,26	108	72.339	97,7	66,63	4.355.336
4	547.445	39,10	79	143.022	97,7	66,63	4.356.215
5	637.650	45,55	67	137.737	97,8	66,63	4.354.335
6	373.821	26,70	104	82.907	97,4	66,63	4.355.956
7	690.264	49,30	107	103.764	97,8	66,28	4.871.626
8	299.841	21,42	181	45.004	97,4	66,24	4.905.665
9	443.995	31,71	109	82.819	97,5	66,24	4.909.200
10	472.760	33,77	94	115.442	95,5	66,63	4.354.518
11	496.712	35,48	105	96.123	97,6	66,29	4.888.781
12	577.089	41,22	86	137.736	98	66,63	4.349.273
13	542.428	38,74	84	98.025	98,2	66,64	4.347.102
14	581.887	41,56	132	67.150	98,1	66,18	4.833.414
15	651.856	46,56	91	133.771	98	66,32	4.869.460
16	574.192	41,01	114	98.044	97,7	66,31	4.868.908
17	444.253	31,73	130	76.566	96,9	66,35	4.804.712
18	463.487	33,11	111	95.154	96,9	66,35	4.800.384
19	376.653	26,90	137	71.949	96	66,35	4.797.657
20	467.010	33,36	106	82.576	97,3	66,35	4.801.064
22	329.721	23,55	136	68.301	96,2	66,35	4.797.610
23	332.597	23,76	148	64.087	95,9	66,42	4.790.996
24	367.194	26,23	141	59.983	95,4	66,15	4.842.464
25	684.296	48,88	118	78.126	97,9	66,14	4.846.530
26	342.187	24,44	175	57.930	95,6	66,21	4.936.760
27	657.616	46,97	68	125.713	97,6	66,51	4.463.214
28	384.635	27,47	123	77.053	97,3	66,31	4.796.803
29	683.609	48,83	130	83.165	97,9	66,32	4.937.645
30	557.032	39,79	123	83.803	97,7	66,32	4.939.361
31	520.625	37,19	100	83.752	97,4	66,33	4.905.248
32	656.796	46,91	96	124.530	97,1	66,15	4.939.680
33	592.461	42,32	142	86.828	97,6	66,24	4.975.717
34	504.079	36,01	96	115.124	97,1	66,63	4.530.605
35	653.900	46,71	81	97.004	97,5	66,79	4.350.184
36	609.726	43,55	63	111.485	97,3	66,79	4.349.041
37	759.185	54,23	70	115.518	97,5	66,79	4.348.768
38	515.839	36,85	85	104.601	97,3	66,8	4.342.069
39	628.107	44,86	70	104.672	97,33	66,79	4.350.454
40	608.106	43,44	82	107.247	97,33	66,79	4.340.930
41	573.166	40,94	78	100.367	97,8	66,79	4.346.125
42	490.227	35,02	98	80.068	96,9	66,79	4.344.612
43	579.745	41,41	75	96.579	96,6	66,8	4.347.965
45	512.522	36,61	72	122.934	96,1	66,79	4.349.228
46	443.034	31,65	85	106.782	96,9	66,79	4.345.979
47	435.752	31,13	105	73.205	97	66,79	4.345.910
48	516.706	36,91	98	78.811	96,5	66,78	4.344.781
49	350.611	25,04	109	86.306	97,44	66,79	4.368.938
50	387.577	27,68	103	88.039	97,5	66,49	4.615.168

51	462.407	33,03	88	83.639	97,5	66,49	4.612.267
52	474.483	33,89	134	67.037	97,1	66,43	4.604.200
53	477.993	34,14	80	117.956	97,8	66,45	4.774.412
54	382.390	27,31	98	91.209	97,2	66,44	4.657.158
55	382.697	27,34	109	68.565	97,2	66,46	4.650.229
56	411.830	29,42	105	77.141	97,2	66,46	4.651.334
57	373.024	26,64	119	69.055	97	66,46	4.648.365
58	336.079	24,01	117	71.979	96,9	66,46	4.650.208
59	425.502	30,39	132	76.828	97,1	66,34	4.720.331
60	463.212	33,09	123	82.508	96,6	66,4	4.651.562
61	413.102	29,51	89	94.682	97,4	66,34	4.827.344
62	428.439	30,60	135	73.002	96,7	66,42	4.616.995
63	340.349	24,31	146	64.353	96,6	66,27	4.869.988
64	366.325	26,17	136	64.953	96,5	66,26	4.906.983
65	525.975	37,57	99	97.603	96,7	66,3	5.015.330
66	614.288	43,88	98	107.275	97,1	66,27	4.913.778
67	461.580	32,97	95	110.927	97,2	66,49	4.735.262
68	464.054	33,15	95	127.350	96,8	66,49	4.736.495
69	468.378	33,46	118	83.722	97,4	66,01	5.214.241
70	431.667	30,83	97	112.847	96,7	66,49	4.739.130
71	361.208	25,80	110	94.504	96,8	66,49	4.735.422
72	406.542	29,04	104	105.012	96,9	66,5	4.731.968
73	411.930	29,42	92	123.704	97	66,5	4.740.614
74	377.861	26,99	105	85.193	96,6	66,49	4.618.853
75	469.960	33,57	96	100.302	96,9	66,49	4.738.647
76	410.329	29,31	92	122.444	96,4	66,48	4.629.835
77	471.214	33,66	90	131.596	97,2	66,52	4.686.641
78	469.420	33,53	89	115.812	96,9	66,28	4.888.041
79	444.401	31,74	91	100.158	96,9	66,28	4.882.433
80	483.549	34,54	88	109.537	96,9	66,81	4.378.819
81	431.155	30,80	152	59.251	96,6	66,44	4.795.198
82	414.230	29,59	97	78.077	96,8	66,81	4.377.916
83	416.675	29,76	89	100.888	96,9	66,81	4.380.841
84	431.822	30,84	94	92.631	96,5	66,81	4.381.561
85	463.227	33,09	81	92.765	97,03	66,81	4.379.631
86	380.846	27,20	116	83.791	96,6	66,55	4.606.604
87	416.356	29,74	106	96.785	96,9	66,41	4.823.756
88	444.087	31,72	106	98.561	96,4	66,4	4.825.907
89	464.228	33,16	92	107.273	97,1	66,56	4.763.892
90	408.629	29,19	115	82.239	97,3	66,43	4.789.620
91	433.936	31,00	97	96.676	97,4	66,63	4.758.044
92	531.679	37,98	103	97.464	97,62	66,12	4.909.362
93	574.885	41,06	80	123.778	97,6	66,26	4.801.838

Library	Mutations					
	COMPLEX	DEL	INS	MNP	SNP	Total
1	4.979	203	184	983	29.794	36.143
2	7.181	281	262	1.411	39.860	48.995
3	6.047	247	246	1.156	36.066	43.762
4	6.391	272	277	1.220	36.673	44.833
5	6.519	286	280	1.244	36.820	45.149
6	5.928	235	238	1.140	35.780	43.321
7	7.299	287	261	1.439	40.047	49.333
8	5.380	182	172	1.057	34.515	41.306
9	6.888	255	232	1.363	39.352	48.090
10	6.362	265	261	1.211	36.534	44.633
11	7.043	268	258	1.401	39.669	48.639
12	6.485	281	281	1.243	36.779	45.069
13	6.451	277	274	1.235	36.754	44.991
14	35.105	590	454	5.912	110.366	152.427
15	7.746	305	305	1.514	41.006	50.876
16	7.627	299	283	1.485	40.780	50.474
17	7.339	278	241	1.421	40.277	49.556
18	7.425	264	258	1.451	40.550	49.948
19	6.971	242	228	1.369	39.668	48.478
20	7.459	280	260	1.450	40.531	49.980
22	6.579	232	199	1.277	38.637	46.924
23	32.106	450	378	5.499	102.620	141.053
24	6.573	238	219	1.305	39.943	48.278
25	7.497	291	295	1.484	41.771	51.338
26	6.005	198	176	1.199	36.977	44.555
27	36.162	571	552	6.136	110.084	153.505
28	27.275	340	362	4.686	101.088	133.751
29	7.082	262	258	1.376	38.997	47.975
30	6.966	259	240	1.339	38.756	47.560
31	6.921	250	243	1.348	38.714	47.476
32	3.925	132	141	792	18.534	23.524
33	7.012	278	267	1.380	39.058	47.995
34	7.218	231	241	1.356	39.404	48.450
35	41.568	715	591	6.997	114.968	164.839
36	41.396	689	575	6.971	114.816	164.447
37	42.115	753	609	7.110	115.498	166.085
38	40.367	648	541	6.813	114.123	162.492
39	41.271	709	581	6.949	114.862	164.372
40	41.259	691	593	6.929	114.727	164.199
41	40.741	660	555	6.867	114.450	163.273
42	39.835	633	517	6.727	113.451	161.163
43	40.767	663	564	6.877	114.313	163.184
45	39.712	620	508	6.675	113.518	161.033
46	39.099	588	482	6.547	112.742	159.458
47	38.933	579	492	6.546	112.675	159.225
48	40.220	631	538	6.775	113.832	161.996
49	36.401	529	424	6.075	109.062	152.491
50	6.269	243	221	1.239	37.852	45.824

51	6.525	260	256	1.312	38.589	46.942
52	5.916	217	204	1.102	33.414	40.853
53	6.625	270	266	1.321	38.715	47.197
54	6.195	242	227	1.262	37.710	45.636
55	6.115	251	230	1.213	37.549	45.358
56	6.213	243	231	1.250	37.964	45.901
57	6.145	243	217	1.220	37.552	45.377
58	5.791	209	217	1.157	36.633	44.007
59	5.972	216	218	1.155	33.257	40.818
60	6.145	229	225	1.163	33.604	41.366
61	29.231	420	421	5.005	103.039	138.116
62	6.101	235	224	1.163	33.503	41.226
63	6.142	211	201	1.218	37.295	45.067
64	6.335	222	207	1.229	37.894	45.887
65	7.133	271	268	1.403	40.054	49.129
66	7.376	287	273	1.427	40.353	49.716
67	7.059	222	241	1.332	39.058	47.912
68	7.028	220	247	1.338	38.944	47.777
69	6.260	196	193	1.143	32.087	39.879
70	6.922	226	236	1.319	38.746	47.449
71	6.551	199	224	1.228	37.857	46.059
72	6.852	222	246	1.291	38.500	47.111
73	6.972	227	250	1.308	38.860	47.617
74	6.503	196	214	1.231	37.527	45.671
75	6.985	222	236	1.312	38.935	47.690
76	6.700	204	221	1.254	38.020	46.399
77	7.043	231	234	1.326	39.018	47.852
78	6.727	261	236	1.321	38.602	47.147
79	29.879	416	426	5.097	104.841	140.659
80	38.912	611	504	6.623	113.001	159.651
81	7.835	241	244	1.510	43.266	53.096
82	37.601	571	475	6.332	111.422	156.401
83	38.154	559	467	6.452	112.241	157.873
84	38.668	591	488	6.524	112.609	158.880
85	38.889	592	494	6.609	113.026	159.610
86	5.434	200	208	1.051	33.248	40.141
87	7.145	229	249	1.417	38.583	47.623
88	7.084	231	234	1.404	38.519	47.472
89	5.794	216	235	1.114	34.069	41.428
90	6.766	205	241	1.343	37.837	46.392
91	7.044	230	246	1.401	38.659	47.580
92	6.783	272	256	1.332	38.653	47.296
93	7.129	225	235	1.335	35.390	44.314

Library	Annotations			
	genes	CDS	tRNA	rRNA
1	4.230	4.102	76	6
2	4.559	4.443	73	7
3	4.042	3.936	71	7
4	4.041	3.934	72	5
5	4.038	3.933	73	7
6	4.040	3.933	72	7
7	4.536	4.416	75	7
8	4.596	4.481	71	7
9	4.594	4.475	75	7
10	4.042	3.936	71	7
11	4.554	4.435	74	4
12	4.031	3.927	72	4
13	4.025	3.921	72	7
14	4.510	4.395	73	6
15	4.587	4.468	77	7
16	4.585	4.465	76	7
17	4.519	4.400	76	7
18	4.516	4.395	78	7
19	4.514	4.398	73	4
20	4.508	4.396	73	11
22	4.510	4.394	75	6
23	4.408	4.300	72	6
24	4.463	4.359	73	4
25	4.473	4.370	71	4
26	4.617	4.506	71	6
27	4.130	4.029	71	5
28	4.403	4.297	72	5
29	4.607	4.493	77	7
30	4.623	4.508	77	8
31	4.582	4.469	77	6
32	4.569	4.450	76	7
33	4.644	4.530	73	6
34	4.171	4.058	78	5
35	3.984	3.875	73	6
36	3.987	3.877	73	6
37	3.984	3.875	72	7
38	3.972	3.864	72	6
39	3.986	3.877	72	6
40	3.987	3.877	72	7
41	3.980	3.870	73	6
42	3.986	3.878	72	6
43	3.975	3.871	70	4
45	3.979	3.876	70	3
46	3.981	3.877	70	4
47	3.988	3.881	72	4
48	3.993	3.884	72	6
49	4.018	3.909	73	6
50	4.282	4.173	73	6

51	4.273	4.161	73	6
52	4.225	4.111	76	7
53	4.434	4.323	73	6
54	4.322	4.206	75	6
55	4.321	4.208	74	8
56	4.316	4.205	73	6
57	4.314	4.203	73	7
58	4.317	4.208	72	7
59	4.368	4.245	76	6
60	4.302	4.188	76	7
61	4.456	4.350	71	6
62	4.275	4.158	76	6
63	4.549	4.435	74	9
64	4.602	4.488	75	7
65	4.669	4.552	77	7
66	4.607	4.490	75	7
67	4.419	4.303	75	7
68	4.419	4.300	77	6
69	4.881	4.755	82	6
70	4.418	4.300	76	6
71	4.424	4.304	77	6
72	4.408	4.290	76	7
73	4.417	4.301	76	6
74	4.319	4.204	75	6
75	4.408	4.294	74	6
76	4.326	4.210	77	6
77	4.345	4.226	77	6
78	4.570	4.456	74	6
79	4.540	4.435	70	7
80	4.023	3.919	70	6
81	4.519	4.407	75	6
82	4.030	3.921	74	6
83	4.037	3.926	74	7
84	4.034	3.925	74	7
85	4.040	3.930	73	6
86	4.229	4.118	76	6
87	4.498	4.386	76	7
88	4.512	4.397	78	7
89	4.388	4.275	78	9
90	4.501	4.383	79	6
91	4.488	4.374	76	9
92	4.621	4.506	73	6
93	4.494	4.384	74	7

Library	Phenotypes						
	Biof	Growth	Swim	Twitch	Swarm	Mutation	Virul
1	0,6276	0,483	15	16	5	2,63E-08	21
2	37.033	0,2191	4	4	4	9,98E-08	29
3	0,4004	0,3088	8	9	3	1,85E-08	27
4	0,6977	0,319	11	11	4	4,67E-08	17
5	0,1279	0,338	6	5	3	0,00000008	22
6	0,7746	0,2973	7	9	3	6,24E-08	24
7	0,3399	0,438	10	14	6	0,000000107	0
8	0,4495	0,3977	7	9	5	0,000001009	4
9	0,3658	0,3265	7	9	3	7,33E-08	0
10	0,6713	0,3854	7	8	2	1,27E-08	24
11	0,3714	0,3531	9	10	4	1,26E-07	0
12	0,0923	0,4635	4	3	3	2,42E-08	26
13	0,0708	0,418	2	3	2	2,95E-08	0
14	0,4369	0,5006	8	17	5	3,91E-08	0
15	0,1563	0,4206	5	3	2	6,83E-08	0
16	0,2405	0,4313	6	2	2	0,000000514	20
17	0,4169	0,4584	8	5	2	0,00000103	7
18	0,3623	0,5212	2	2	4	5,40E-09	3
19	0,4683	0,4505	6	2	3	0,000000241	0
20	0,0887	0,3775	7	2	3	0,000000254	0
22	0,0023	0,3876	3	2	3	0,00000129	0
23	0,4605	0,5442	20	10	6	8,29E-08	0
24	0,1256	0,369	10	13	5	0,00000108	0
25	0,1287	0,2571	5	2	2	0,000000631	4
26	0,3745	0,4277	6	8	5	4,81E-08	0
27	0,3377	0,5802	13	16	4	1,74E-08	3
28	4.052	0,1869	3	4	3	4,10E-09	5
29	0,3337	0,5128	16	18	7	6,49E-08	0
30	0,3083	0,4958	21	17	8	0,000000128	19
31	0,3161	0,4899	16	17	9	5,54E-08	0
32	0,4829	0,5483	22	16	6	2,72E-08	0
33	0,3104	0,4741	22	10	4	4,56E-08	0
34	0,1828	0,3627	11	5	5	2,04E-08	0
35	0	0,3868	2	3	3	0,000000726	18
36	0,0176	0,4028	2	2	3	0,000000786	22
37	0,0291	0,3964	2	4	3	0,000000903	0
38	0,0087	0,389	2	3	2	0,0000013	0
39	0,0028	0,3953	2	2	4	0,00000141	0
40	0,5693	0,2968	2	8	3	0,000000712	0
41	0,0611	0,3952	2	4	3	0,00000128	0
42	0,01	0,3918	2	3	3	0,00000125	4
43	0,0751	0,3563	3	3	3	0,00000153	0
45	0,0011	0,3317	2	3	3	0,00000629	11
46	0,0214	0,316	2	3	2	0,00000918	0
47	0,3886	0,2907	2	6	3	0,00000459	23
48	0,0797	0,3804	2	3	2	0,00000713	10
49	0,4772	0,2379	2	2	2	0,00000233	9
50	0,1816	0,4761	11	8	4	0,000000202	0

51	0,1932	0,3944	11	8	4	0,000000441	0
52	0,7344	0,5754	23	16	7	1,81E-08	0
53	0,1859	0,4087	10	9	4	3,28E-08	0
54	0,2069	0,3856	6	11	4	0,00000073	0
55	0,3003	0,4311	12	7	4	0,000000197	0
56	0,2746	0,4131	13	8	4	6,92E-08	0
57	0,3359	0,4452	13	10	5	0,000000068	0
58	0,233	0,4268	6	8	3	7,18E-08	0
59	0,5769	0,3582	12	6	3	0,000000775	27
60	0	0,5003	11	2	4	2,42E-08	0
61	0,4131	0,4248	17	7	4	4,23E-08	0
62	0,0038	0,3636	10	2	3	8,82E-08	21
63	0,3668	0,5249	6	6	3	0,000000256	0
64	0,4166	0,4945	9	5	3	0,000000772	2
65	0,3071	0,4808	8	7	4	0,000000086	8
66	0,5122	0,4557	5	3	4	0,00000228	0
67	0,4176	0,5562	9	5	3	5,81E-08	28
68	0,4066	0,5849	4	6	3	0,000000216	16
69	53.023	0,5046	11	16	5	0,00000008	4
70	0,5727	0,2603	13	3	4	5,75E-08	15
71	0,063	0,4844	5	3	3	4,14E-08	2
72	0,0034	0,3962	8	11	4	0,000000233	0
73	0,007	0,3843	7	10	4	0,000000209	1
74	0,0097	0,7839	9	11	3	0,000000121	6
75	0,0023	0,3408	9	6	4	0,000000146	15
76	0,0031	0,3569	11	4	3	0,000000106	14
77	0,0027	0,3197	12	13	2	0,000000143	14
78	0,3798	0,5378	15	13	6	5,22E-08	0
79	0,3366	0,4695	4	7	4	0,00000021	0
80	0,3289	0,5453	13	10	5	0,000000015	0
81	0,3642	0,5172	7	13	5	6,34E-08	0
82	0,1848	0,4377	8	8	3	2,11E-08	0
83	0,1003	0,4314	8	7	2	0,000000043	0
84	0,1098	0,3795	2	3	2	1,12E-08	13
85	0,1192	0,4324	5	7	2	1,92E-08	0
86	0,8576	0,5577	17	17	9	4,21E-06	25
87	0,5251	0,4939	8	16	3	0,00000312	14
88	0,2751	0,443	6	11	2	0,000000284	0
89	0,7257	0,5079	5	4	3	0,00000052	4
90	0,6769	0,437	10	15	6	2,21E-07	0
91	0,7973	0,5142	15	14	6	0,000000135	3
92	0,3984	0,5426	19	13	7	0,000000161	12
93	0,448	0,4454	7	13	4	0,000000142	0
