# Supplementary material for: Evolution of Stenotrophomonas maltophilia in Cystic Fibrosis Lung over Chronic Infection: A Genomic and Phenotypic Population Study
Source: Front Microbiol. 2017 Aug 28;8:1590. doi: 10.3389/fmicb.2017.01590 (PMC5581383; doi:10.3389/fmicb.2017.01590)
Supplement: Supplementary file 2 [file Table2.PDF]

Supplementary table 2 - Average nucleotide identity (ANI) calculated through pairwise blast. In the table are reported the ANI values (lower triangular half) and the alignment length (upper triangular half). Reference strain K279a and type strain 13637 are also included.

	16	43	71	67	89	79	54	77	55	49
16	-	3609161	4175643	4177041	4138026	3862744	4375093	4168023	4366032	3595469
43	0,914	-	3637840	3642108	3638482	3510460	3609913	3644778	3606203	4306948
71	0,979	0,914	-	4717821	4254459	3906819	4207548	4661928	4203355	3632815
67	0,979	0,914	1,000	-	4261234	3882464	4205516	4658186	4203517	3634700
89	0,981	0,914	0,982	0,982	-	3894956	4266856	4321254	4264161	3714608
79	0,933	0,910	0,933	0,933	0,933	-	3822409	3910330	3821972	3492147
54	0,994	0,914	0,981	0,981	0,981	0,933	-	4153512	4629503	3612433
77	0,980	0,913	1,000	1,000	0,982	0,933	0,981	-	4153890	3635696
55	0,995	0,914	0,981	0,981	0,981	0,933	1,000	0,981	-	3610411
49	0,915	0,999	0,915	0,915	0,915	0,911	0,915	0,915	0,915	-
47	0,915	1,000	0,914	0,914	0,914	0,910	0,914	0,914	0,914	1,000
15	1,000	0,914	0,979	0,979	0,980	0,934	0,995	0,980	0,994	0,914
28	0,932	0,910	0,933	0,933	0,933	0,999	0,933	0,933	0,933	0,910
13	0,983	0,915	0,982	0,982	0,982	0,934	0,984	0,982	0,984	0,915
8	0,998	0,914	0,980	0,980	0,979	0,933	0,994	0,980	0,994	0,914
26	0,997	0,914	0,978	0,978	0,980	0,932	0,994	0,978	0,994	0,915
45	0,914	1,000	0,914	0,914	0,914	0,910	0,914	0,914	0,914	1,000
29	0,979	0,913	0,979	0,979	0,982	0,934	0,980	0,979	0,980	0,913
73	0,980	0,913	1,000	1,000	0,982	0,933	0,981	1,000	0,981	0,914
36	0,915	1,000	0,914	0,914	0,915	0,911	0,915	0,914	0,915	1,000
53	0,995	0,914	0,981	0,981	0,981	0,933	1,000	0,981	1,000	0,914
18	1,000	0,914	0,980	0,980	0,980	0,934	0,995	0,980	0,994	0,914
70	0,980	0,914	1,000	1,000	0,982	0,933	0,981	1,000	0,981	0,914
12	0,983	0,915	0,982	0,982	0,982	0,934	0,984	0,982	0,984	0,915
75	0,980	0,914	1,000	1,000	0,982	0,933	0,981	1,000	0,981	0,914
65	0,996	0,915	0,978	0,978	0,980	0,933	0,995	0,978	0,995	0,915
37	0,915	1,000	0,914	0,914	0,914	0,910	0,914	0,914	0,914	1,000
76	0,980	0,914	1,000	1,000	0,982	0,934	0,981	1,000	0,981	0,914
41	0,914	1,000	0,914	0,914	0,914	0,910	0,914	0,914	0,914	1,000
17	1,000	0,915	0,979	0,979	0,980	0,934	0,994	0,980	0,994	0,915

1	0,980	0,914	0,982	0,982	0,984	0,934	0,982	0,982	0,982	0,915
86	0,980	0,915	0,982	0,982	1,000	0,933	0,981	0,982	0,981	0,915
85	0,914	0,989	0,914	0,914	0,915	0,911	0,914	0,914	0,914	0,989
24	0,979	0,914	0,977	0,977	0,979	0,933	0,980	0,977	0,980	0,914
39	0,915	1,000	0,914	0,914	0,914	0,911	0,915	0,914	0,915	1,000
Type_strain_13637	0,978	0,913	0,977	0,977	0,978	0,932	0,980	0,977	0,980	0,913
34	0,981	0,914	0,999	0,999	0,982	0,934	0,981	1,000	0,981	0,914
30	0,979	0,913	0,979	0,979	0,982	0,934	0,980	0,979	0,980	0,913
46	0,915	1,000	0,914	0,914	0,914	0,910	0,914	0,914	0,915	0,999
66	0,996	0,914	0,978	0,978	0,980	0,932	0,994	0,978	0,994	0,914
4	0,983	0,915	0,982	0,981	0,982	0,935	0,984	0,982	0,984	0,915
59	0,981	0,915	0,980	0,980	0,982	0,933	0,981	0,980	0,981	0,915
38	0,915	1,000	0,914	0,914	0,914	0,910	0,915	0,914	0,915	1,000
35	0,915	1,000	0,914	0,914	0,914	0,911	0,914	0,914	0,914	1,000
7	0,998	0,914	0,980	0,980	0,979	0,933	0,994	0,980	0,994	0,915
40	0,915	1,000	0,914	0,914	0,914	0,911	0,915	0,914	0,915	1,000
11	0,998	0,914	0,980	0,980	0,979	0,933	0,994	0,980	0,994	0,914
20	1,000	0,914	0,979	0,979	0,981	0,933	0,994	0,980	0,994	0,915
82	0,914	0,989	0,914	0,914	0,915	0,911	0,914	0,914	0,914	0,989
63	0,996	0,915	0,978	0,978	0,980	0,932	0,994	0,978	0,994	0,915
5	0,983	0,915	0,982	0,982	0,982	0,934	0,984	0,982	0,984	0,915
48	0,915	1,000	0,914	0,914	0,914	0,910	0,914	0,914	0,914	1,000
27	0,923	0,913	0,925	0,924	0,924	0,921	0,923	0,924	0,923	0,913
92	0,997	0,914	0,978	0,978	0,979	0,932	0,995	0,978	0,994	0,914
50	0,995	0,915	0,981	0,981	0,981	0,933	1,000	0,981	1,000	0,915
31	0,979	0,913	0,979	0,979	0,982	0,934	0,980	0,979	0,980	0,913
84	0,914	0,989	0,914	0,914	0,915	0,911	0,914	0,914	0,914	0,989
2	0,998	0,915	0,980	0,980	0,979	0,933	0,994	0,980	0,994	0,915
91	0,980	0,914	0,981	0,981	0,982	0,933	0,980	0,981	0,980	0,914
33	0,997	0,915	0,978	0,978	0,979	0,933	0,994	0,978	0,994	0,915
10	0,983	0,914	0,982	0,982	0,982	0,934	0,984	0,982	0,984	0,914
83	0,914	0,989	0,914	0,914	0,915	0,911	0,914	0,914	0,914	0,989
56	0,995	0,915	0,981	0,981	0,981	0,933	1,000	0,981	1,000	0,915

32	0,978	0,914	0,980	0,980	0,983	0,932	0,979	0,980	0,979	0,914
K279a	0,979	0,914	0,980	0,980	0,984	0,933	0,981	0,980	0,981	0,914
81	0,978	0,915	0,977	0,977	0,977	0,932	0,979	0,977	0,979	0,915
22	1,000	0,914	0,980	0,980	0,980	0,934	0,994	0,980	0,994	0,914
9	0,998	0,914	0,980	0,980	0,979	0,933	0,994	0,980	0,994	0,914
64	0,996	0,914	0,978	0,978	0,980	0,932	0,994	0,978	0,994	0,914
62	0,980	0,914	0,981	0,981	0,982	0,933	0,980	0,981	0,981	0,914
78	0,998	0,914	0,978	0,978	0,980	0,932	0,995	0,978	0,995	0,915
42	0,914	1,000	0,914	0,914	0,914	0,911	0,914	0,914	0,914	1,000
88	0,979	0,914	0,980	0,980	0,982	0,933	0,979	0,980	0,979	0,914
25	0,979	0,914	0,978	0,978	0,979	0,932	0,980	0,978	0,980	0,914
90	0,980	0,914	0,981	0,981	0,982	0,933	0,979	0,981	0,979	0,914
93	0,979	0,914	0,979	0,980	0,982	0,932	0,979	0,979	0,979	0,914
80	0,914	0,989	0,914	0,914	0,915	0,911	0,914	0,914	0,914	0,989
87	0,980	0,914	0,980	0,980	0,982	0,933	0,979	0,980	0,979	0,914
72	0,980	0,914	1,000	1,000	0,982	0,933	0,981	1,000	0,981	0,914
58	0,995	0,914	0,981	0,981	0,981	0,933	1,000	0,981	1,000	0,914
23	0,916	0,929	0,915	0,915	0,916	0,911	0,916	0,915	0,917	0,929
74	0,980	0,914	1,000	1,000	0,982	0,934	0,981	1,000	0,981	0,914
52	0,980	0,914	0,981	0,981	0,982	0,933	0,980	0,981	0,980	0,914
68	0,980	0,913	1,000	1,000	0,982	0,933	0,981	1,000	0,981	0,914
19	1,000	0,914	0,980	0,980	0,980	0,934	0,994	0,980	0,994	0,914
14	0,924	0,911	0,926	0,926	0,924	0,919	0,924	0,926	0,924	0,912
60	0,981	0,914	0,981	0,981	0,982	0,933	0,981	0,981	0,981	0,915
3	0,983	0,914	0,982	0,982	0,982	0,934	0,984	0,982	0,984	0,914
61	0,932	0,910	0,933	0,933	0,933	1,000	0,932	0,933	0,932	0,910
51	0,995	0,914	0,981	0,981	0,981	0,933	1,000	0,981	1,000	0,915
57	0,995	0,915	0,981	0,981	0,981	0,933	1,000	0,981	1,000	0,915
6	0,983	0,915	0,982	0,982	0,982	0,934	0,984	0,982	0,984	0,915
69	0,977	0,914	0,979	0,979	0,981	0,932	0,979	0,979	0,979	0,914

47	15	28	13	8	26	45	29	73	36	53
3608184	4853039	3769304	4098334	4445124	4478384	3610290	4212817	4182802	3619064	4373265
4315697	3605896	3493203	3523449	3576601	3583764	4332610	3626528	3650129	4334054	3616207
3639898	4188940	3873228	4075922	4276714	4176742	3649831	4288066	4719246	3655877	4210089
3642850	4187369	3851411	4083149	4270653	4174600	3654192	4276137	4717587	3655427	4212767
3723539	4259232	3897036	4124891	4234322	4290372	3734937	4437027	4365551	3740568	4273845
3495885	3870065	4625809	3725861	3897908	3824115	3513040	3861891	3912514	3516081	3828900
3625708	4380754	3776055	4069096	4355943	4328239	3630616	4170370	4202984	3640878	4640656
3646381	4174178	3875402	4079885	4267099	4162956	3656938	4236960	4664712	3664675	4162996
3619298	4372631	3776315	4067825	4350030	4329324	3625522	4167633	4205456	3632385	4634083
4340489	3617415	3505180	3531642	3587054	3589722	4349872	3625956	3652469	4354147	3624313
-	3612381	3488541	3521287	3578111	3584690	4332924	3625337	3649203	4334764	3619350
0,914	-	3768629	4097216	4440090	4473238	3604812	4203494	4175556	3612337	4373575
0,910	0,932	-	3701593	3831214	3806662	3501427	3872452	3884990	3507271	3782363
0,915	0,983	0,934	-	4034017	4070840	3504839	4001730	4064316	3510494	4067544
0,914	0,998	0,933	0,983	-	4546729	3619090	4314405	4329651	3625463	4394444
0,914	0,997	0,933	0,983	0,997	-	3617022	4305195	4210250	3625941	4363611
1,000	0,914	0,910	0,915	0,915	0,915	-	3603852	3638155	4328382	3606757
0,913	0,979	0,934	0,980	0,979	0,980	0,913	-	4287111	3654420	4186402
0,914	0,980	0,934	0,981	0,981	0,979	0,913	0,980	-	3655673	4209298
1,000	0,915	0,911	0,915	0,915	0,915	1,000	0,914	0,914	-	3610447
0,914	0,995	0,933	0,984	0,995	0,995	0,914	0,980	0,981	0,914	-
0,914	1,000	0,933	0,983	0,998	0,998	0,914	0,979	0,980	0,914	0,994
0,914	0,980	0,934	0,982	0,981	0,979	0,914	0,980	1,000	0,914	0,981
0,915	0,983	0,935	1,000	0,984	0,983	0,915	0,981	0,982	0,915	0,984
0,914	0,980	0,934	0,982	0,980	0,979	0,914	0,980	1,000	0,914	0,981
0,915	0,996	0,933	0,983	0,998	0,999	0,915	0,979	0,978	0,915	0,995
1,000	0,915	0,910	0,915	0,915	0,915	1,000	0,914	0,914	1,000	0,914
0,914	0,980	0,934	0,982	0,981	0,979	0,914	0,980	1,000	0,914	0,981
1,000	0,914	0,910	0,915	0,915	0,915	1,000	0,913	0,914	1,000	0,914
0,915	1,000	0,933	0,982	0,998	0,998	0,914	0,979	0,979	0,915	0,994

0,915	0,980	0,934	0,981	0,982	0,980	0,914	0,982	0,982	0,915	0,982
0,915	0,981	0,933	0,982	0,981	0,981	0,915	0,982	0,982	0,915	0,981
0,989	0,914	0,911	0,915	0,915	0,914	0,989	0,914	0,914	0,989	0,914
0,914	0,979	0,933	0,981	0,980	0,980	0,914	0,979	0,977	0,914	0,980
1,000	0,915	0,911	0,915	0,915	0,915	1,000	0,914	0,914	1,000	0,915
0,913	0,978	0,933	0,980	0,979	0,978	0,913	0,978	0,977	0,913	0,980
0,914	0,981	0,934	0,982	0,981	0,980	0,914	0,980	0,999	0,914	0,981
0,913	0,978	0,934	0,980	0,979	0,980	0,913	1,000	0,980	0,913	0,980
1,000	0,915	0,911	0,915	0,915	0,915	1,000	0,914	0,914	1,000	0,914
0,914	0,996	0,933	0,983	0,998	0,999	0,914	0,979	0,978	0,914	0,994
0,915	0,983	0,935	1,000	0,984	0,983	0,915	0,981	0,982	0,915	0,984
0,915	0,981	0,933	0,982	0,980	0,980	0,915	0,979	0,980	0,915	0,981
1,000	0,915	0,910	0,915	0,915	0,915	1,000	0,914	0,914	1,000	0,914
1,000	0,915	0,911	0,915	0,915	0,915	1,000	0,914	0,914	1,000	0,914
0,914	0,998	0,933	0,983	1,000	0,997	0,914	0,979	0,980	0,914	0,994
1,000	0,915	0,911	0,915	0,915	0,915	1,000	0,914	0,914	1,000	0,915
0,914	0,998	0,933	0,983	1,000	0,997	0,914	0,979	0,980	0,914	0,994
0,915	1,000	0,932	0,982	0,998	0,998	0,914	0,979	0,979	0,914	0,994
0,989	0,914	0,911	0,915	0,914	0,914	0,989	0,914	0,914	0,989	0,914
0,915	0,996	0,933	0,983	0,998	0,999	0,915	0,979	0,978	0,915	0,994
0,915	0,983	0,935	1,000	0,984	0,983	0,915	0,981	0,982	0,915	0,984
1,000	0,915	0,910	0,915	0,915	0,915	1,000	0,913	0,914	1,000	0,914
0,913	0,923	0,921	0,924	0,923	0,923	0,913	0,924	0,924	0,913	0,923
0,914	0,997	0,933	0,983	0,998	0,999	0,914	0,979	0,978	0,914	0,995
0,915	0,995	0,933	0,984	0,994	0,995	0,915	0,980	0,981	0,915	1,000
0,913	0,979	0,934	0,980	0,979	0,980	0,913	1,000	0,979	0,913	0,980
0,989	0,915	0,911	0,915	0,915	0,914	0,989	0,914	0,914	0,989	0,914
0,915	0,998	0,933	0,983	1,000	0,997	0,915	0,979	0,980	0,915	0,994
0,914	0,980	0,933	0,980	0,980	0,980	0,914	0,981	0,981	0,914	0,980
0,915	0,997	0,933	0,983	0,998	1,000	0,915	0,979	0,978	0,915	0,994
0,914	0,983	0,935	1,000	0,984	0,983	0,914	0,981	0,982	0,914	0,984
0,989	0,914	0,911	0,914	0,915	0,914	0,989	0,914	0,914	0,989	0,914
0,915	0,995	0,933	0,984	0,995	0,995	0,915	0,980	0,981	0,915	1,000

0,914	0,978	0,932	0,981	0,978	0,979	0,914	0,980	0,980	0,914	0,979
0,914	0,979	0,933	0,981	0,980	0,980	0,914	0,982	0,980	0,914	0,981
0,915	0,979	0,933	0,980	0,978	0,978	0,915	0,976	0,977	0,915	0,979
0,914	1,000	0,933	0,982	0,998	0,998	0,914	0,979	0,980	0,914	0,994
0,914	0,998	0,933	0,983	1,000	0,997	0,914	0,979	0,980	0,914	0,994
0,914	0,996	0,933	0,983	0,998	0,999	0,914	0,978	0,978	0,914	0,994
0,914	0,980	0,934	0,981	0,981	0,980	0,914	0,980	0,981	0,914	0,981
0,914	0,998	0,933	0,983	0,998	1,000	0,914	0,979	0,978	0,914	0,995
1,000	0,914	0,911	0,914	0,915	0,915	1,000	0,913	0,914	1,000	0,914
0,914	0,980	0,933	0,980	0,979	0,979	0,914	0,980	0,980	0,914	0,979
0,914	0,979	0,932	0,981	0,980	0,980	0,914	0,979	0,978	0,914	0,980
0,914	0,980	0,933	0,980	0,980	0,979	0,914	0,980	0,981	0,914	0,979
0,914	0,979	0,932	0,980	0,979	0,978	0,914	0,979	0,979	0,914	0,979
0,989	0,914	0,911	0,914	0,915	0,914	0,989	0,914	0,914	0,989	0,914
0,914	0,980	0,933	0,980	0,979	0,979	0,914	0,980	0,980	0,914	0,979
0,914	0,979	0,934	0,982	0,980	0,979	0,914	0,980	1,000	0,914	0,981
0,914	0,995	0,933	0,984	0,994	0,995	0,914	0,980	0,981	0,914	1,000
0,929	0,916	0,911	0,916	0,916	0,916	0,929	0,915	0,915	0,929	0,916
0,914	0,980	0,934	0,981	0,981	0,979	0,914	0,980	1,000	0,914	0,981
0,914	0,980	0,933	0,982	0,981	0,981	0,914	0,981	0,981	0,914	0,980
0,914	0,980	0,933	0,982	0,981	0,979	0,913	0,980	1,000	0,913	0,981
0,914	1,000	0,933	0,982	0,998	0,998	0,914	0,979	0,980	0,914	0,994
0,911	0,924	0,919	0,925	0,926	0,924	0,911	0,924	0,926	0,911	0,924
0,914	0,981	0,933	0,982	0,981	0,980	0,914	0,980	0,981	0,914	0,981
0,914	0,983	0,935	1,000	0,984	0,983	0,914	0,981	0,982	0,914	0,984
0,910	0,932	1,000	0,933	0,933	0,933	0,910	0,933	0,933	0,910	0,932
0,915	0,995	0,933	0,984	0,994	0,995	0,914	0,980	0,981	0,914	1,000
0,915	0,995	0,933	0,984	0,994	0,994	0,915	0,980	0,981	0,915	1,000
0,915	0,983	0,934	1,000	0,984	0,983	0,915	0,981	0,982	0,915	0,984
0,914	0,977	0,933	0,980	0,978	0,978	0,914	0,979	0,979	0,914	0,979

18	70	12	75	65	37	76	41	17	1	86
4774176	4177606	4097437	4181183	4523215	3612952	4096474	3614491	4775146	4130223	4132909
3598164	3641062	3523776	3646362	3604013	4332113	3583221	4330453	3595063	3616584	3627357
4183429	4720797	4076246	4717611	4207292	3652939	4614108	3652646	4182027	4193947	4246094
4171322	4710675	4083816	4713028	4205555	3654410	4607631	3651223	4171446	4183859	4250215
4249189	4370362	4125429	4370513	4286674	3733643	4274233	3732852	4243994	4317527	4688452
3857440	3908508	3728265	3908780	3842097	3510663	3845019	3508633	3854858	3776952	3784889
4371080	4198981	4068669	4201513	4350840	3632120	4118419	3633669	4372652	4112539	4143781
4163941	4656849	4078212	4661816	4194996	3658563	4554333	3657958	4161028	4190767	4199627
4361798	4199229	4064305	4202893	4348185	3629614	4119454	3630615	4357468	4116078	4158346
3609510	3647830	3529629	3652727	3614748	4352726	3588039	4351705	3608891	3621712	3632049
3603582	3639784	3524690	3642311	3608268	4330946	3582033	4330720	3601505	3611606	3622331
4772846	4172594	4098298	4175197	4525451	3611744	4090823	3607191	4769376	4138684	4129189
3773452	3884064	3703031	3882300	3825083	3502151	3818785	3501677	3771676	3765776	3803893
4085495	4054999	4327989	4060986	4070120	3504553	4038382	3507012	4078721	4022801	3986013
4482504	4321649	4080763	4329112	4502493	3619864	4245144	3621253	4472891	4133614	4130905
4514133	4202702	4100059	4207386	4722860	3621487	4101201	3620726	4513070	4154864	4171048
3592410	3632407	3504785	3635904	3594399	4332430	3575406	4325401	3585657	3604772	3608877
4213585	4288124	4017473	4285551	4302007	3651016	4194917	3647139	4209612	4209575	4304707
4168574	4713323	4081854	4720148	4202369	3653679	4607364	3652214	4167307	4189236	4251334
3597033	3635813	3513685	3639604	3596561	4327824	3581715	4327057	3591682	3608561	3617903
4488112	4317577	4187232	4316955	4468280	3689690	4231868	3690357	4482378	4223037	4256762
-	4171241	4099252	4175123	4528651	3621863	4094891	3618837	4779811	4142489	4127644
0,980	-	4077339	4717114	4206505	3631706	4613163	3632905	4170682	4193951	4238413
0,983	0,982	-	4054091	4065167	3517772	4030759	3516986	4079443	4015935	3989192
0,980	1,000	0,982	-	4200977	3658759	4608505	3658611	4172371	4191787	4246851
0,996	0,978	0,983	0,978	-	3750679	4275113	3747911	4677671	4289563	4316971
0,915	0,914	0,915	0,914	0,914	-	3587204	4331839	3608113	3613231	3627779
0,980	1,000	0,982	1,000	0,979	0,914	-	3590026	4085669	4094062	4151310
0,914	0,914	0,915	0,914	0,914	1,000	0,914	-	3602010	3620551	3631990
1,000	0,979	0,982	0,979	0,998	0,915	0,979	0,915	-	4147822	4134880

0,980	0,982	0,981	0,982	0,982	0,915	0,982	0,914	0,980	-	4208786
0,981	0,982	0,982	0,982	0,981	0,915	0,982	0,915	0,981	0,984	-
0,914	0,914	0,915	0,914	0,914	0,989	0,914	0,989	0,914	0,915	0,915
0,979	0,978	0,981	0,978	0,980	0,914	0,977	0,914	0,979	0,980	0,979
0,915	0,914	0,915	0,914	0,915	1,000	0,914	1,000	0,915	0,914	0,914
0,978	0,977	0,980	0,977	0,979	0,914	0,977	0,913	0,978	0,980	0,979
0,981	0,999	0,982	0,999	0,980	0,914	0,999	0,914	0,981	0,982	0,982
0,979	0,980	0,980	0,980	0,979	0,913	0,979	0,913	0,979	0,982	0,982
0,915	0,914	0,915	0,914	0,915	1,000	0,914	1,000	0,915	0,914	0,914
0,996	0,978	0,983	0,978	1,000	0,914	0,979	0,914	0,996	0,981	0,980
0,983	0,982	1,000	0,982	0,984	0,915	0,982	0,915	0,983	0,982	0,982
0,981	0,980	0,982	0,980	0,980	0,915	0,980	0,915	0,981	0,983	0,982
0,915	0,914	0,915	0,914	0,915	1,000	0,914	1,000	0,915	0,915	0,914
0,915	0,914	0,915	0,914	0,914	1,000	0,914	1,000	0,915	0,914	0,914
0,998	0,980	0,983	0,980	0,998	0,914	0,980	0,914	0,998	0,982	0,980
0,915	0,914	0,915	0,914	0,915	1,000	0,914	1,000	0,915	0,915	0,914
0,998	0,980	0,983	0,980	0,998	0,914	0,980	0,914	0,998	0,981	0,980
1,000	0,979	0,982	0,979	0,997	0,914	0,980	0,914	1,000	0,981	0,980
0,914	0,914	0,915	0,914	0,914	0,989	0,914	0,989	0,914	0,914	0,915
0,996	0,978	0,983	0,978	1,000	0,915	0,979	0,915	0,996	0,981	0,980
0,983	0,982	1,000	0,982	0,983	0,915	0,982	0,915	0,983	0,982	0,982
0,915	0,914	0,915	0,914	0,915	1,000	0,914	1,000	0,915	0,914	0,914
0,923	0,925	0,924	0,925	0,923	0,913	0,925	0,913	0,923	0,924	0,924
0,997	0,978	0,983	0,978	0,999	0,914	0,979	0,914	0,997	0,981	0,980
0,995	0,981	0,984	0,981	0,995	0,915	0,981	0,915	0,995	0,982	0,981
0,979	0,979	0,980	0,979	0,979	0,913	0,979	0,913	0,979	0,982	0,982
0,914	0,914	0,915	0,914	0,914	0,989	0,914	0,989	0,914	0,915	0,915
0,998	0,980	0,983	0,980	0,998	0,915	0,980	0,915	0,998	0,981	0,980
0,980	0,981	0,980	0,981	0,979	0,914	0,981	0,914	0,980	0,982	0,982
0,997	0,978	0,983	0,978	0,999	0,915	0,979	0,915	0,997	0,980	0,979
0,983	0,982	1,000	0,982	0,983	0,914	0,982	0,914	0,983	0,982	0,982
0,914	0,914	0,915	0,914	0,914	0,989	0,914	0,989	0,914	0,914	0,915
0,995	0,981	0,984	0,981	0,995	0,915	0,981	0,915	0,995	0,982	0,981

0,978	0,980	0,981	0,980	0,979	0,914	0,980	0,914	0,978	0,985	0,983
0,979	0,981	0,981	0,980	0,980	0,914	0,980	0,914	0,979	0,986	0,984
0,979	0,977	0,979	0,977	0,978	0,915	0,977	0,915	0,979	0,978	0,978
1,000	0,980	0,982	0,980	0,997	0,914	0,980	0,914	1,000	0,981	0,980
0,998	0,980	0,983	0,980	0,998	0,914	0,980	0,914	0,998	0,981	0,980
0,996	0,978	0,983	0,978	1,000	0,914	0,979	0,914	0,996	0,981	0,980
0,981	0,981	0,981	0,981	0,980	0,914	0,981	0,914	0,980	0,982	0,982
0,998	0,978	0,983	0,978	0,999	0,914	0,979	0,914	0,998	0,980	0,980
0,914	0,914	0,914	0,914	0,914	1,000	0,914	1,000	0,914	0,914	0,914
0,980	0,980	0,980	0,980	0,978	0,914	0,980	0,914	0,980	0,981	0,982
0,979	0,978	0,981	0,978	0,981	0,914	0,978	0,914	0,979	0,980	0,979
0,980	0,981	0,980	0,981	0,978	0,914	0,981	0,914	0,980	0,981	0,982
0,979	0,980	0,980	0,979	0,979	0,914	0,979	0,914	0,979	0,982	0,982
0,914	0,914	0,914	0,914	0,914	0,989	0,914	0,989	0,914	0,915	0,915
0,980	0,980	0,980	0,980	0,978	0,914	0,980	0,914	0,980	0,981	0,982
0,979	1,000	0,982	1,000	0,979	0,914	1,000	0,914	0,980	0,982	0,982
0,995	0,981	0,984	0,981	0,995	0,914	0,981	0,914	0,994	0,982	0,981
0,916	0,915	0,916	0,915	0,916	0,929	0,916	0,929	0,916	0,916	0,916
0,980	1,000	0,981	1,000	0,979	0,914	1,000	0,914	0,980	0,981	0,982
0,980	0,982	0,982	0,981	0,980	0,914	0,981	0,914	0,980	0,984	0,982
0,980	1,000	0,982	1,000	0,979	0,914	1,000	0,914	0,980	0,982	0,982
1,000	0,980	0,982	0,979	0,997	0,915	0,980	0,914	1,000	0,980	0,981
0,924	0,926	0,925	0,926	0,924	0,911	0,927	0,911	0,924	0,924	0,924
0,981	0,981	0,982	0,981	0,980	0,915	0,981	0,915	0,981	0,983	0,982
0,983	0,982	1,000	0,982	0,984	0,914	0,982	0,914	0,983	0,982	0,982
0,932	0,933	0,933	0,933	0,932	0,910	0,933	0,910	0,932	0,933	0,933
0,995	0,981	0,984	0,981	0,995	0,915	0,981	0,914	0,995	0,982	0,981
0,995	0,981	0,984	0,981	0,995	0,915	0,981	0,915	0,995	0,982	0,981
0,983	0,982	1,000	0,981	0,983	0,915	0,982	0,915	0,983	0,982	0,982
0,977	0,979	0,980	0,979	0,978	0,914	0,979	0,914	0,977	0,985	0,981

85	24	39	Type_strain_13637	34	30	46	66	4	59
3647229	4170794	3619070	4269022	4161447	4212011	3609588	4525256	4107234	4145370
4059178	3609609	4333558	3631753	3616925	3630982	4324138	3605083	3525484	3576647
3697290	4137956	3654786	4220621	4426569	4284507	3644859	4202773	4085701	4188926
3695496	4139387	3653700	4223461	4420817	4278422	3646173	4201991	4093290	4184961
3760104	4279218	3733661	4377376	4297732	4435559	3725087	4283537	4136329	4236641
3507573	3847125	3511850	3854817	3803120	3858416	3498116	3839339	3728510	3770265
3644176	4137830	3637472	4216833	4137023	4165973	3623292	4351817	4080779	4087164
3694662	4139681	3660846	4217971	4417455	4233219	3647894	4195837	4090193	4186615
3633968	4130181	3632611	4213013	4139663	4166397	3617986	4348477	4074826	4087909
4081119	3619810	4352834	3637365	3624943	3629477	4336841	3617468	3534103	3581412
4075624	3607492	4334229	3627469	3617885	3628312	4324605	3609867	3528944	3576889
3642349	4171304	3610653	4271142	4149847	4208060	3603343	4525548	4108834	4139258
3492100	3792257	3504935	3816075	3796009	3869125	3490227	3824401	3705506	3762060
3533588	3974052	3509071	4096917	4039734	4001626	3494195	4071882	4326359	4004468
3650580	4225129	3625021	4323890	4141603	4318290	3615995	4502087	4092164	4158380
3667168	4193115	3623288	4401827	4161046	4302080	3615106	4719574	4104776	4157631
4061194	3596133	4330768	3621324	3613327	3607488	4318180	3597334	3508122	3568243
3659848	4197146	3652602	4323060	4183704	4918744	3637803	4299819	4026091	4159474
3691257	4144898	3655514	4224579	4424826	4288286	3641609	4204221	4094526	4192669
4057582	3605928	4332511	3625225	3616918	3616299	4319759	3596111	3517458	3569593
3692035	4258578	3692313	4334377	4251019	4283375	3679144	4468060	4193094	4194698
3644180	4164560	3621308	4276534	4156344	4215338	3608075	4527636	4108488	4140708
3670573	4128934	3639428	4223612	4422135	4284454	3629555	4200903	4091067	4185719
3534622	3977268	3519740	4098469	4041476	4002054	3509248	4064210	4333980	4002263
3692339	4142271	3664576	4221750	4422983	4279845	3656174	4201000	4080628	4192107
3808737	4328530	3754420	4450240	4334394	4447110	3742564	4998732	4250964	4264539
4067017	3611193	4334992	3636498	3625149	3640097	4323419	3616951	3532151	3582208
3606669	4037674	3595409	4127049	4314544	4182623	3580466	4102842	4058332	4087571
4068291	3615429	4335544	3646609	3629117	3637988	4320090	3613796	3542511	3584850
3643506	4177173	3618661	4277161	4159683	4217169	3605800	4527689	4112183	4144070

3623840	4096562	3618606	4235147	4177786	4189845	3603635	4129436	4060949	4181781
3639855	4156935	3619182	4229894	4185006	4291953	3601408	4171867	4017304	4129004
-	3626998	4054639	3650551	3637771	3625304	4034308	3647250	3536959	3576557
0,914	-	3625283	4246233	4115285	4181791	3615062	4199571	4011601	4060865
0,988	0,914	-	3635600	3619560	3620591	4320787	3602366	3521684	3576780
0,913	0,979	0,913	-	4168371	4272975	3619535	4279180	4091576	4124618
0,914	0,979	0,914	0,978	-	4172741	3629573	4189563	4060454	4121970
0,913	0,979	0,913	0,978	0,980	-	3645553	4302385	4035186	4163899
0,989	0,914	1,000	0,914	0,914	0,913	-	3611774	3532641	3582502
0,913	0,980	0,914	0,978	0,980	0,979	0,914	-	4103242	4126812
0,914	0,981	0,915	0,980	0,982	0,981	0,915	0,984	-	4005526
0,914	0,979	0,915	0,977	0,982	0,979	0,915	0,980	0,982	-
0,989	0,914	1,000	0,914	0,914	0,914	1,000	0,915	0,915	0,915
0,988	0,914	1,000	0,914	0,914	0,914	1,000	0,914	0,915	0,915
0,914	0,979	0,914	0,978	0,981	0,979	0,914	0,998	0,983	0,979
0,989	0,914	1,000	0,914	0,915	0,914	1,000	0,915	0,915	0,915
0,913	0,979	0,914	0,978	0,981	0,979	0,914	0,998	0,983	0,979
0,913	0,979	0,914	0,978	0,980	0,979	0,914	0,997	0,982	0,980
1,000	0,914	0,989	0,914	0,914	0,914	0,989	0,914	0,915	0,915
0,913	0,980	0,915	0,978	0,980	0,979	0,915	1,000	0,982	0,980
0,914	0,982	0,915	0,980	0,982	0,981	0,915	0,983	1,000	0,982
0,988	0,914	1,000	0,914	0,914	0,913	1,000	0,915	0,915	0,915
0,913	0,923	0,913	0,924	0,925	0,924	0,913	0,923	0,924	0,925
0,913	0,980	0,914	0,978	0,980	0,980	0,914	0,999	0,983	0,979
0,914	0,981	0,915	0,980	0,981	0,980	0,915	0,995	0,983	0,981
0,913	0,979	0,913	0,978	0,980	1,000	0,913	0,979	0,980	0,978
1,000	0,914	0,989	0,914	0,914	0,914	0,989	0,914	0,915	0,915
0,914	0,979	0,915	0,978	0,980	0,979	0,915	0,998	0,983	0,979
0,914	0,979	0,914	0,977	0,981	0,981	0,914	0,979	0,980	0,980
0,914	0,979	0,915	0,977	0,980	0,979	0,915	0,999	0,982	0,979
0,914	0,981	0,914	0,980	0,982	0,981	0,914	0,983	1,000	0,982
1,000	0,914	0,989	0,913	0,914	0,914	0,989	0,914	0,914	0,915
0,914	0,980	0,915	0,980	0,981	0,980	0,915	0,995	0,983	0,980

0,914	0,978	0,914	0,977	0,980	0,980	0,914	0,979	0,981	0,981
0,914	0,980	0,914	0,977	0,981	0,982	0,914	0,980	0,981	0,981
0,914	0,979	0,915	0,975	0,978	0,976	0,915	0,978	0,980	0,977
0,913	0,980	0,914	0,978	0,980	0,979	0,914	0,997	0,982	0,980
0,914	0,979	0,914	0,978	0,980	0,979	0,914	0,998	0,983	0,979
0,912	0,980	0,914	0,978	0,979	0,978	0,914	1,000	0,982	0,979
0,914	0,978	0,914	0,977	0,981	0,980	0,914	0,980	0,981	1,000
0,913	0,980	0,914	0,977	0,980	0,979	0,914	0,999	0,983	0,980
0,988	0,914	1,000	0,914	0,914	0,913	1,000	0,914	0,914	0,915
0,914	0,978	0,914	0,976	0,980	0,980	0,914	0,978	0,980	0,979
0,914	1,000	0,914	0,979	0,979	0,979	0,914	0,981	0,981	0,978
0,914	0,978	0,914	0,976	0,980	0,980	0,914	0,978	0,980	0,980
0,913	0,979	0,914	0,976	0,980	0,979	0,914	0,979	0,980	0,981
1,000	0,914	0,989	0,914	0,914	0,914	0,989	0,914	0,914	0,915
0,914	0,978	0,914	0,976	0,980	0,980	0,914	0,978	0,979	0,979
0,913	0,978	0,914	0,977	0,999	0,980	0,914	0,979	0,981	0,980
0,913	0,980	0,914	0,980	0,981	0,980	0,914	0,995	0,983	0,981
0,929	0,916	0,929	0,915	0,916	0,915	0,929	0,916	0,916	0,916
0,913	0,978	0,914	0,977	0,999	0,980	0,914	0,979	0,981	0,980
0,914	0,978	0,914	0,978	0,982	0,981	0,914	0,980	0,982	0,999
0,913	0,978	0,913	0,977	0,999	0,980	0,913	0,979	0,981	0,980
0,914	0,980	0,914	0,978	0,980	0,979	0,914	0,997	0,982	0,980
0,911	0,924	0,911	0,923	0,924	0,924	0,911	0,924	0,925	0,922
0,915	0,978	0,914	0,977	0,982	0,980	0,914	0,980	0,982	1,000
0,914	0,981	0,914	0,980	0,982	0,981	0,914	0,983	1,000	0,982
0,910	0,932	0,910	0,932	0,933	0,933	0,910	0,933	0,933	0,932
0,914	0,980	0,914	0,980	0,981	0,980	0,914	0,995	0,983	0,981
0,914	0,981	0,915	0,980	0,981	0,980	0,915	0,995	0,984	0,981
0,914	0,981	0,915	0,980	0,982	0,981	0,915	0,983	1,000	0,982
0,914	0,977	0,914	0,975	0,980	0,979	0,914	0,978	0,980	0,980

38	35	7	40	11	20	82	63	5	48	27
3611684	3613562	4477213	3605226	4472239	4774881	3646954	4504064	4105816	3606570	3714087
4319642	4332166	3604668	4319966	3605500	3596817	4053327	3586435	3524508	4318962	3634279
3645807	3652261	4297324	3645093	4295730	4187115	3691780	4187270	4090257	3645043	3760510
3647296	3654739	4297228	3645464	4287053	4180186	3691597	4183496	4095486	3642320	3752109
3729035	3737779	4276330	3725650	4271659	4254260	3753601	4260720	4135119	3720318	3845501
3501954	3510286	3921766	3505585	3915611	3866973	3498562	3821417	3734474	3501352	3689556
3631998	3630914	4394548	3626720	4391281	4371231	3641518	4333284	4078397	3625985	3717882
3653054	3659728	4289438	3648536	4285258	4164784	3689530	4172323	4095826	3647180	3760541
3623494	3631884	4386031	3624806	4376981	4367089	3633939	4326424	4072102	3623644	3722795
4341265	4351861	3617032	4346618	3613161	3613735	4076268	3597362	3536856	4342280	3644135
4323234	4332209	3617222	4322775	3607171	3608614	4071362	3589449	3527947	4325022	3629854
3604388	3610797	4473692	3603444	4470954	4771875	3639332	4496201	4106448	3599873	3709560
3494097	3502930	3861998	3494170	3858817	3778404	3489022	3807800	3706847	3490854	3676342
3498986	3506775	4050176	3502626	4048355	4090743	3527954	4052927	4330710	3492858	3632608
3619032	3619230	4842763	3615031	4847147	4480343	3646390	4489054	4087030	3617800	3721210
3618686	3622649	4545258	3613757	4544205	4511510	3660423	4707250	4102956	3610738	3734681
4318653	4324638	3599625	4316737	3591750	3593369	4056721	3572649	3510964	4312679	3622483
3642306	3646740	4323287	3639963	4324939	4221440	3654756	4278535	4029084	3639011	3741817
3646416	3654655	4294045	3640606	4291401	4177057	3686823	4181248	4098538	3643103	3770388
4321842	4325908	3603344	4313947	3600106	3594951	4052042	3578730	3519749	4318011	3628687
3685097	3692700	4502444	3686847	4497218	4486530	3689312	4450940	4191384	3680116	3804880
3618263	3618139	4480571	3612273	4476739	4782085	3644051	4500803	4106619	3610821	3703928
3632172	3633683	4293401	3620006	4288296	4175418	3668535	4175990	4093426	3624621	3745510
3511154	3519722	4041280	3511895	4038748	4086318	3528361	4041524	4333682	3501311	3624501
3652921	3658533	4298390	3649624	4290863	4174769	3687708	4185203	4086100	3648744	3747899
3750270	3749197	4631762	3739419	4627755	4684006	3804032	4975163	4248975	3741900	3870896
4325342	4333792	3618046	4319570	3611301	3616164	4060935	3594626	3528961	4321239	3645612
3584503	3591680	4206264	3583621	4201122	4089494	3603786	4085550	4061061	3585027	3685018
4321296	4329490	3612761	4315473	3609532	3610051	4064286	3593016	3543029	4318586	3639716
3616090	3616382	4476114	3609034	4474237	4780951	3635959	4508548	4110119	3605862	3712434

3608662	3612553	4120656	3605081	4117128	4157111	3621890	4113238	4064879	3602452	3726261
3610051	3613704	4128729	3607330	4125013	4133575	3629923	4150098	4017881	3608065	3740341
4037170	4048613	3611432	4040902	3609952	3631743	4353174	3632195	3535871	4036834	3614855
3620215	3624761	4212608	3617772	4206504	4189004	3643194	4184291	4007566	3615640	3733621
4322826	4332513	3605549	4318565	3603578	3602356	4060065	3585889	3522533	4317997	3628779
3622401	3629349	4253549	3619128	4252361	4262665	3651437	4255564	4093429	3617958	3741088
3634652	3638195	4141394	3629309	4132192	4162739	3649398	4160677	4058324	3625973	3745284
3651650	3656671	4329991	3648987	4329494	4224737	3660152	4282264	4035926	3645722	3733816
4323132	4327802	3616075	4319333	3612941	3610610	4059347	3591899	3534786	4316423	3636480
3621940	3627022	4479422	3619398	4477005	4536366	3676391	4816121	4098705	3615781	3732974
3519830	3527336	4050505	3520705	4044478	4090241	3540535	4052719	4344381	3514852	3635229
3580140	3585755	4158213	3579197	4158221	4136330	3585342	4103893	4029835	3576998	3703376
-	4328081	3604138	4315958	3599873	3597627	4061640	3579991	3518358	4320605	3629440
1,000	-	3605756	4322238	3601228	3604306	4063672	3586940	3516274	4319830	3638182
0,914	0,915	-	3606692	4844372	4472462	3620833	4461086	4071170	3606074	3704030
1,000	1,000	0,915	-	3609871	3611309	4053634	3596351	3533552	4316540	3620271
0,914	0,914	1,000	0,914	-	4489559	3635745	4486902	4086400	3620973	3740477
0,914	0,914	0,998	0,914	0,998	-	3646048	4499255	4109578	3611959	3712912
0,989	0,989	0,914	0,989	0,914	0,914	-	3634232	3543441	4038716	3630742
0,915	0,915	0,998	0,915	0,998	0,996	0,913	-	4106726	3607843	3732549
0,915	0,915	0,984	0,915	0,984	0,983	0,914	0,984	-	3510523	3615064
1,000	1,000	0,915	0,999	0,915	0,915	0,989	0,915	0,915	-	3641331
0,913	0,913	0,923	0,913	0,923	0,923	0,913	0,923	0,924	0,913	-
0,914	0,914	0,999	0,914	0,998	0,997	0,913	0,999	0,983	0,914	0,923
0,915	0,915	0,995	0,915	0,995	0,995	0,914	0,995	0,983	0,915	0,924
0,913	0,913	0,979	0,913	0,979	0,979	0,913	0,979	0,980	0,913	0,923
0,989	0,989	0,915	0,989	0,915	0,914	1,000	0,914	0,914	0,989	0,913
0,915	0,915	1,000	0,915	1,000	0,998	0,914	0,998	0,983	0,915	0,924
0,914	0,914	0,980	0,914	0,980	0,980	0,914	0,979	0,980	0,914	0,923
0,915	0,915	0,998	0,915	0,998	0,997	0,914	0,999	0,982	0,915	0,923
0,914	0,914	0,984	0,914	0,984	0,983	0,914	0,984	1,000	0,914	0,925
0,989	0,989	0,914	0,989	0,914	0,914	1,000	0,914	0,914	0,989	0,913
0,915	0,915	0,995	0,915	0,995	0,995	0,914	0,995	0,983	0,915	0,924

0,914	0,914	0,978	0,914	0,978	0,978	0,914	0,979	0,981	0,914	0,924
0,914	0,914	0,980	0,914	0,980	0,979	0,914	0,980	0,981	0,914	0,925
0,914	0,915	0,978	0,915	0,978	0,979	0,914	0,978	0,980	0,915	0,925
0,914	0,914	0,998	0,914	0,998	1,000	0,913	0,997	0,982	0,914	0,924
0,914	0,914	1,000	0,914	1,000	0,998	0,914	0,998	0,983	0,914	0,923
0,914	0,914	0,999	0,914	0,998	0,996	0,912	1,000	0,983	0,914	0,923
0,914	0,914	0,981	0,914	0,981	0,980	0,914	0,980	0,981	0,914	0,925
0,914	0,915	0,999	0,914	0,999	0,998	0,913	0,999	0,983	0,914	0,923
1,000	1,000	0,914	0,999	0,914	0,914	0,988	0,914	0,914	1,000	0,913
0,914	0,914	0,979	0,914	0,979	0,979	0,914	0,978	0,980	0,914	0,923
0,914	0,914	0,980	0,914	0,980	0,979	0,914	0,981	0,981	0,914	0,923
0,914	0,914	0,979	0,914	0,979	0,980	0,914	0,978	0,980	0,914	0,923
0,914	0,914	0,978	0,914	0,979	0,979	0,913	0,979	0,980	0,914	0,923
0,989	0,989	0,915	0,989	0,915	0,914	1,000	0,914	0,914	0,989	0,913
0,914	0,914	0,979	0,914	0,979	0,980	0,914	0,979	0,979	0,914	0,923
0,914	0,914	0,981	0,914	0,981	0,979	0,913	0,979	0,981	0,914	0,925
0,914	0,914	0,994	0,914	0,994	0,994	0,913	0,995	0,983	0,914	0,924
0,929	0,929	0,917	0,929	0,917	0,916	0,929	0,916	0,916	0,929	0,912
0,914	0,914	0,981	0,914	0,981	0,980	0,913	0,979	0,981	0,914	0,925
0,914	0,914	0,981	0,914	0,981	0,980	0,914	0,981	0,982	0,914	0,924
0,913	0,914	0,981	0,914	0,981	0,980	0,913	0,979	0,981	0,914	0,925
0,914	0,915	0,998	0,914	0,998	1,000	0,914	0,997	0,982	0,915	0,923
0,911	0,912	0,926	0,911	0,926	0,924	0,911	0,925	0,925	0,911	0,928
0,914	0,915	0,981	0,915	0,981	0,981	0,915	0,980	0,982	0,915	0,925
0,914	0,914	0,984	0,914	0,984	0,983	0,914	0,984	1,000	0,914	0,925
0,910	0,910	0,933	0,910	0,933	0,932	0,910	0,933	0,933	0,910	0,921
0,914	0,915	0,995	0,914	0,994	0,995	0,914	0,995	0,983	0,914	0,924
0,915	0,915	0,995	0,915	0,995	0,995	0,914	0,995	0,984	0,915	0,924
0,915	0,915	0,984	0,915	0,984	0,983	0,914	0,984	1,000	0,915	0,925
0,914	0,914	0,979	0,914	0,979	0,977	0,914	0,978	0,980	0,914	0,924

92	50	31	84	2	91	33	10	83	56	32
4467012	4370729	4209781	3648890	4485649	4200375	4510117	4095529	3650048	4365860	4204095
3576086	3613918	3625373	4056823	3609214	3644163	3606593	3519675	4058647	3608225	3649724
4189235	4205877	4282655	3693874	4315467	4230449	4202812	4076811	3693979	4204259	4245948
4183345	4205834	4277234	3691072	4313527	4232730	4203980	4084736	3693310	4203139	4247802
4292151	4263549	4426240	3759128	4280597	4358843	4326325	4125683	3757778	4261714	4387690
3822810	3825052	3858410	3504352	3935064	3787841	3846774	3717260	3506641	3824371	3856465
4314663	4594528	4166359	3642055	4393924	4188683	4344402	4066846	3642296	4632622	4172915
4175999	4158533	4232789	3693382	4304921	4190646	4193250	4080260	3693397	4154806	4249350
4305611	4588220	4166970	3634241	4388577	4182862	4341763	4065765	3636330	4625492	4174906
3598848	3623705	3625578	4083933	3614116	3642538	3613329	3527493	4081540	3620817	3652976
3586357	3615346	3626855	4074987	3611557	3639336	3606495	3520033	4073110	3610783	3652418
4466830	4363706	4206179	3640198	4489981	4196935	4510298	4097060	3641471	4365708	4202790
3799367	3782984	3872348	3491925	3863747	3781034	3823990	3698331	3492599	3778975	3807708
4046834	4061008	3996002	3532976	4073779	4000587	4090552	4321946	3528788	4058378	4015603
4524666	4391601	4317316	3647850	4865158	4206347	4556994	4084082	3647351	4387442	4222653
4732817	4362471	4299432	3664675	4563811	4223420	4886785	4098233	3664668	4353738	4246814
3572515	3606484	3606273	4063323	3600223	3623666	3590371	3502816	4060560	3601472	3638923
4296137	4183760	4903607	3657334	4330338	4343822	4310908	4018390	3657474	4176342	4294049
4185890	4203815	4286736	3687147	4313272	4236698	4201305	4086620	3688661	4200055	4249599
3574836	3609831	3614052	4056372	3598483	3633710	3597895	3512891	4056608	3601286	3637334
4427753	4706436	4282623	3691898	4502779	4304991	4458594	4186810	3694529	4744900	4284301
4459965	4374110	4210877	3641961	4495419	4201251	4510476	4099872	3641839	4370527	4195903
4185929	4195882	4281655	3671709	4312512	4232243	4198915	4083786	3673870	4191559	4235693
4041609	4056885	3999945	3532262	4066958	4006332	4084217	4322416	3533291	4057561	4016945
4183566	4207178	4281391	3686476	4314935	4232762	4198259	4073898	3690731	4203240	4235667
4867537	4493527	4444396	3810846	4665090	4357518	4873941	4237099	3807870	4488251	4368082
3593611	3619364	3631444	4067475	3615385	3636160	3615858	3525541	4064704	3617929	3650419
4080713	4111968	4178562	3605858	4226607	4125507	4100790	4048163	3606292	4110551	4140189
3591901	3616042	3628040	4071864	3614916	3641575	3613394	3535922	4074102	3616095	3653226
4460779	4375798	4219044	3639239	4496566	4206899	4516578	4101300	3640555	4369488	4204146

4114361	4100308	4190173	3621579	4121401	4176679	4166797	4055285	3621126	4098808	4256654
4150699	4159460	4286269	3636018	4129617	4201865	4176502	4008660	3635225	4151338	4258868
3633203	3617708	3622659	4363306	3628372	3653326	3648737	3531280	4363633	3618577	3643520
4168850	4163298	4187216	3647197	4216212	4140844	4202044	4002431	3648869	4156586	4284883
3583702	3608060	3618469	4063273	3607247	3633818	3605544	3516835	4064660	3606300	3640645
4305258	4204449	4277238	3657798	4275172	4153065	4360985	4084882	3662561	4198971	4339532
4153696	4142084	4171865	3654526	4140918	4217267	4160608	4052526	3652435	4145054	4184074
4296687	4181856	4903620	3658806	4331943	4342065	4313995	4025754	3660592	4175310	4291722
3586920	3622924	3640102	4061066	3615518	3653394	3611316	3525879	4064898	3616520	3659005
4726375	4346659	4283331	3680290	4512266	4198299	4718642	4091748	3683429	4339631	4221859
4046010	4065717	4001736	3543115	4073192	4001061	4094949	4335613	3543599	4067131	4023438
4114736	4088752	4157886	3589896	4160189	4097161	4152823	4018523	3589127	4088328	4212559
3579248	3610401	3609803	4067936	3604150	3632346	3598449	3513000	4065143	3603632	3636688
3583259	3611616	3611935	4068413	3606429	3626027	3606808	3508127	4066733	3607675	3643458
4511442	4392044	4318346	3620367	4828318	4196509	4542321	4065751	3621569	4391555	4210447
3586576	3619719	3618784	4055348	3617067	3633989	3610419	3525452	4055363	3615057	3650573
4528786	4412605	4333755	3637951	4844639	4216980	4557304	4081174	3641026	4406584	4228124
4462773	4371982	4201985	3647258	4486245	4199795	4513356	4097585	3648327	4365282	4209318
3636938	3620096	3630121	4356914	3632975	3652933	3651364	3539540	4359495	3616766	3648432
4719374	4355122	4301237	3674298	4519063	4212053	4732834	4097880	3676771	4350108	4227938
4046516	4068272	3997900	3535301	4070677	4001183	4092748	4329811	3532445	4064002	4023552
3599232	3628877	3626712	4070517	3623732	3643212	3621256	3528753	4072748	3622447	3659914
3713584	3715212	3715117	3637783	3707831	3713894	3723061	3630436	3638106	3709630	3731256
-	4316771	4258525	3660953	4504793	4166815	4728807	4057545	3661869	4304402	4193885
0,995	-	4173561	3636933	4395791	4190848	4349111	4080639	3635623	4590444	4173760
0,980	0,980	-	3653216	4315118	4308849	4283215	4021144	3650077	4174492	4288509
0,914	0,914	0,914	-	3630733	3654561	3648348	3541613	4366850	3623752	3654449
0,998	0,994	0,979	0,914	-	4200002	4554620	4077875	3637439	4376557	4206104
0,979	0,980	0,980	0,914	0,980	-	4285256	4100112	3739490	4283799	4282231
0,999	0,994	0,979	0,914	0,998	0,979	-	4133434	3697611	4371001	4290741
0,983	0,984	0,981	0,914	0,983	0,980	0,983	-	3547981	4062305	4029117
0,914	0,914	0,914	1,000	0,914	0,914	0,914	0,915	-	3615667	3644662
0,995	1,000	0,980	0,914	0,995	0,980	0,995	0,983	0,914	-	4170379

0,979	0,979	0,980	0,914	0,978	0,980	0,978	0,981	0,914	0,979	-
0,980	0,981	0,982	0,914	0,980	0,980	0,979	0,981	0,914	0,981	0,990
0,978	0,979	0,976	0,914	0,978	0,976	0,978	0,980	0,914	0,979	0,975
0,998	0,994	0,979	0,913	0,998	0,980	0,998	0,982	0,913	0,994	0,979
0,998	0,994	0,979	0,914	1,000	0,979	0,997	0,983	0,914	0,994	0,978
0,999	0,994	0,978	0,912	0,998	0,978	0,999	0,982	0,912	0,994	0,979
0,980	0,981	0,980	0,914	0,980	0,980	0,980	0,981	0,914	0,980	0,981
0,999	0,995	0,979	0,913	0,998	0,979	1,000	0,983	0,913	0,995	0,979
0,914	0,914	0,913	0,988	0,914	0,914	0,914	0,914	0,988	0,914	0,914
0,979	0,979	0,980	0,914	0,979	0,999	0,979	0,980	0,914	0,979	0,979
0,980	0,980	0,979	0,914	0,980	0,978	0,980	0,981	0,914	0,980	0,979
0,979	0,979	0,980	0,914	0,979	0,999	0,979	0,980	0,914	0,979	0,980
0,979	0,979	0,979	0,913	0,978	0,980	0,978	0,980	0,913	0,979	0,984
0,914	0,914	0,914	1,000	0,914	0,914	0,914	0,914	1,000	0,914	0,915
0,979	0,979	0,980	0,914	0,979	0,999	0,979	0,979	0,914	0,979	0,979
0,979	0,981	0,980	0,914	0,980	0,981	0,978	0,981	0,913	0,981	0,981
0,995	1,000	0,980	0,913	0,994	0,980	0,995	0,983	0,913	1,000	0,980
0,916	0,917	0,915	0,929	0,916	0,915	0,916	0,916	0,929	0,917	0,915
0,979	0,981	0,980	0,913	0,981	0,981	0,979	0,981	0,913	0,981	0,980
0,981	0,980	0,980	0,914	0,981	0,980	0,981	0,982	0,914	0,980	0,982
0,979	0,981	0,980	0,913	0,981	0,981	0,979	0,981	0,913	0,981	0,981
0,998	0,994	0,979	0,914	0,998	0,980	0,998	0,982	0,914	0,994	0,979
0,924	0,925	0,924	0,911	0,926	0,924	0,924	0,925	0,911	0,924	0,922
0,980	0,981	0,980	0,915	0,981	0,980	0,980	0,982	0,915	0,981	0,981
0,984	0,984	0,981	0,914	0,984	0,981	0,983	1,000	0,914	0,984	0,982
0,932	0,932	0,933	0,910	0,933	0,933	0,933	0,933	0,910	0,932	0,931
0,995	1,000	0,980	0,914	0,994	0,979	0,995	0,983	0,914	1,000	0,980
0,995	1,000	0,980	0,914	0,994	0,980	0,994	0,984	0,914	1,000	0,980
0,984	0,984	0,981	0,914	0,984	0,981	0,983	1,000	0,914	0,984	0,982
0,978	0,979	0,979	0,914	0,978	0,980	0,978	0,980	0,914	0,979	0,986

K279a	81	22	9	64	62	78	42	88	25	90
4265795	4185948	4760782	4488309	4505978	4138345	4502927	3606640	4203068	4181798	4198263
3671888	3592708	3588286	3604115	3591512	3578763	3605483	4316016	3633049	3614568	3639809
4266166	4073856	4173754	4313570	4195728	4149760	4201882	3644784	4230609	4151461	4224514
4263873	4069043	4162113	4308553	4188026	4146451	4202916	3641806	4230493	4153007	4222139
4412959	4194437	4235530	4275373	4272773	4238837	4313343	3726533	4351610	4291364	4351188
3865754	3795476	3849197	3926685	3830604	3733217	3840732	3499110	3781059	3862112	3781332
4186535	4095921	4358629	4387016	4332794	4086042	4353946	3623101	4191447	4149291	4190846
4269390	4073479	4148612	4300459	4184350	4145406	4190607	3648469	4189356	4157304	4184001
4185367	4090390	4349680	4378474	4333109	4087377	4348811	3618642	4201070	4142969	4190946
3668485	3599792	3595717	3614912	3609733	3580709	3620022	4340939	3636999	3629028	3640124
3664528	3598455	3593939	3611885	3594835	3580389	3606425	4321508	3631928	3617691	3633679
4262049	4184659	4749487	4488284	4504232	4130877	4505741	3599210	4195396	4184096	4188550
3823461	3726738	3768580	3858650	3816380	3715206	3823590	3494638	3777409	3802096	3776550
4079462	3965695	4069865	4065378	4055161	4004335	4093496	3497496	3995718	3989311	3994396
4218151	4207692	4477864	4892016	4488754	4108133	4567520	3617604	4203030	4228379	4201588
4229650	4186251	4501321	4561983	4710528	4153016	4836379	3612764	4252673	4198709	4255238
3654190	3583472	3578525	3595242	3583666	3568605	3594728	4316601	3618637	3613369	3621904
4323238	4162674	4208640	4324108	4285399	4110118	4308608	3637858	4338256	4209225	4334721
4280435	4080172	4157976	4304717	4186829	4147514	4199530	3645199	4230280	4159504	4234335
3652107	3587022	3581962	3597534	3582646	3569353	3597429	4312838	3626789	3610997	3625764
4300205	4201402	4467590	4493802	4445382	4197644	4463052	3678558	4315853	4263052	4304400
4256807	4186386	4764719	4491085	4512367	4134794	4506605	3609632	4203784	4177511	4194352
4264354	4059955	4160399	4307559	4192532	4146638	4201191	3626667	4237653	4136988	4229963
4077166	3971812	4066252	4057245	4048004	4005489	4087307	3505750	3989675	3987580	3993954
4263260	4070029	4156753	4316264	4184871	4150217	4198532	3649232	4226594	4150768	4229347
4416027	4295319	4667689	4652827	4980533	4252879	4895449	3742911	4381008	4340066	4381122
3670271	3602598	3599455	3611321	3604679	3586337	3615523	4316371	3634988	3620475	3631192
4164615	3977461	4073878	4219367	4093759	4051597	4102080	3577139	4129440	4049984	4127554
3667665	3598811	3592583	3611013	3599991	3585033	3613731	4317034	3634887	3624729	3636320
4264387	4189479	4768651	4488796	4515803	4138670	4505258	3601197	4210947	4186276	4203132

4322116	4038638	4138017	4115456	4118503	4182399	4168009	3603139	4167425	4114388	4170293
4289681	4056768	4120171	4124008	4157925	4129653	4169136	3606083	4190309	4174496	4197458
3670535	3604278	3619904	3624508	3632120	3580410	3648986	4036923	3652992	3635610	3651938
4257460	4083310	4172650	4214095	4192662	4061255	4200823	3617581	4134371	4818868	4133420
3658297	3587339	3586316	3602263	3596048	3575308	3604095	4316749	3625828	3617829	3627939
4364775	4182704	4246411	4265747	4259104	4133886	4322995	3618162	4169483	4214595	4157599
4247043	4080315	4142365	4137918	4176396	4119396	4162464	3626398	4207867	4110111	4210756
4319388	4174384	4209051	4328310	4287581	4117611	4315526	3644434	4341075	4204150	4339807
3671685	3604634	3593666	3610050	3597959	3582517	3610299	4319545	3640481	3627683	3646331
4270046	4146796	4516619	4500579	4871168	4114939	4780374	3613856	4225333	4200942	4227790
4077270	3972424	4064857	4065718	4057462	4002647	4099586	3513743	3997302	3994362	3994122
4255305	4034787	4121636	4155384	4114146	4577302	4154914	3581144	4111904	4058258	4090328
3655307	3587440	3580145	3602592	3591455	3570076	3599626	4318932	3621837	3609472	3626278
3657360	3594301	3589575	3603266	3597819	3573280	3608246	4321261	3622065	3610202	3622504
4211171	4180487	4449947	4845085	4469202	4109589	4549965	3608434	4192117	4210977	4187558
3668943	3605008	3592024	3610305	3604933	3584084	3613401	4310629	3623191	3624171	3631558
4227530	4190304	4474956	4857248	4495584	4119330	4567849	3622641	4210871	4229544	4214056
4266383	4187476	4762731	4478989	4508545	4138194	4501741	3605519	4202536	4195059	4196581
3669017	3608527	3617906	3628823	3640000	3590872	3645440	4040099	3639725	3634558	3646217
4265155	4161703	4534563	4516053	4839044	4117346	4752956	3607091	4242246	4208750	4239681
4074629	3969540	4076254	4063809	4063911	4010393	4095413	3509885	3997914	3994829	3995781
3671707	3604575	3606127	3620419	3612868	3594667	3617179	4318195	3638234	3624063	3641913
3746830	3695775	3694779	3702798	3713778	3690972	3721301	3632971	3709114	3728019	3708065
4200320	4100516	4465171	4501834	4718705	4091571	4742187	3594319	4197319	4174452	4199320
4184969	4093092	4358718	4391009	4340659	4084487	4361146	3626536	4191290	4153257	4192897
4318101	4139309	4209305	4306013	4286024	4113527	4294666	3641227	4310117	4200567	4307372
3676539	3620689	3620704	3626967	3636257	3591538	3650022	4052832	3648241	3643666	3645544
4203365	4191502	4463670	4865828	4479290	4098645	4563839	3594148	4193967	4213614	4191478
4339078	4201289	4275684	4265254	4276653	4176865	4273869	3713992	4690728	4214241	4729976
4274617	4222922	4528942	4586926	4730198	4183659	4847864	3644749	4282906	4243798	4282857
4078495	3966826	4078630	4074164	4067904	4008700	4100213	3520564	3999149	4000710	3997067
3669299	3613751	3614585	3622091	3634520	3582933	3645131	4048107	3644928	3633579	3646239
4181777	4090236	4348825	4386011	4331221	4091241	4349434	3621199	4189460	4150166	4181405

4556062	4194630	4224588	4236582	4235183	4247457	4234551	3679346	4242305	4321090	4217466
-	4142634	4233187	4181747	4228651	4229182	4213670	3636801	4251770	4233264	4231081
0,977	-	4221327	4211054	4161544	4059349	4155866	3615054	4199138	4112469	4156319
0,979	0,979	-	4478646	4501113	4124264	4494208	3596057	4198970	4172686	4192528
0,979	0,977	0,998	-	4479712	4101286	4557902	3610060	4185912	4215357	4185768
0,979	0,977	0,996	0,998	-	4113414	4782167	3618028	4240639	4203807	4240367
0,981	0,977	0,981	0,980	0,980	-	4163342	3593148	4119939	4077246	4102693
0,979	0,979	0,998	0,998	0,999	0,980	-	3608711	4220157	4196902	4219407
0,914	0,914	0,914	0,914	0,914	0,915	0,914	-	3629271	3614757	3634280
0,979	0,975	0,980	0,979	0,978	0,979	0,979	0,914	-	4168612	4734337
0,980	0,978	0,979	0,980	0,981	0,978	0,980	0,914	0,978	-	4137780
0,980	0,976	0,980	0,979	0,978	0,979	0,979	0,914	1,000	0,978	-
0,981	0,977	0,979	0,978	0,979	0,981	0,979	0,914	0,980	0,979	0,980
0,914	0,914	0,914	0,915	0,914	0,915	0,914	0,989	0,914	0,914	0,914
0,979	0,975	0,980	0,979	0,978	0,979	0,979	0,914	1,000	0,978	1,000
0,981	0,977	0,979	0,980	0,979	0,981	0,979	0,914	0,981	0,978	0,981
0,980	0,979	0,994	0,994	0,995	0,981	0,995	0,914	0,980	0,980	0,980
0,916	0,917	0,916	0,916	0,916	0,916	0,916	0,929	0,915	0,916	0,915
0,980	0,977	0,980	0,981	0,979	0,981	0,979	0,914	0,981	0,978	0,981
0,983	0,978	0,980	0,981	0,980	0,999	0,981	0,914	0,980	0,978	0,980
0,981	0,977	0,980	0,981	0,979	0,981	0,979	0,913	0,981	0,978	0,981
0,979	0,979	1,000	0,998	0,997	0,980	0,998	0,914	0,980	0,980	0,980
0,923	0,924	0,924	0,926	0,925	0,923	0,924	0,911	0,922	0,924	0,924
0,981	0,977	0,981	0,981	0,980	1,000	0,980	0,915	0,979	0,978	0,979
0,981	0,980	0,983	0,984	0,984	0,982	0,983	0,914	0,981	0,981	0,981
0,932	0,932	0,932	0,933	0,933	0,933	0,933	0,910	0,933	0,932	0,933
0,981	0,979	0,995	0,994	0,995	0,981	0,995	0,914	0,979	0,980	0,980
0,981	0,979	0,995	0,995	0,995	0,981	0,995	0,915	0,979	0,981	0,980
0,982	0,980	0,983	0,984	0,983	0,982	0,983	0,915	0,981	0,981	0,981
0,981	0,974	0,977	0,978	0,978	0,980	0,978	0,914	0,979	0,977	0,980

93	80	87	72	58	23	74	52	68	19	14
4219760	3647696	4205996	4176409	4356070	3723911	4083972	4162091	4180542	4761152	3838051
3620630	4063429	3638135	3647559	3603610	3666789	3580601	3589967	3642499	3588683	3569735
4253768	3692934	4231661	4709231	4200562	3730501	4602787	4158478	4723145	4176631	3901261
4248894	3692856	4234000	4709001	4197598	3720901	4594812	4156571	4714467	4161468	3896812
4350258	3757396	4347978	4366966	4256743	3785156	4273208	4260103	4373365	4237588	3912830
3853720	3506635	3777301	3912549	3815822	3613484	3840317	3781553	3912131	3856413	3806181
4224397	3641343	4195962	4193840	4622843	3721312	4112856	4143894	4203098	4360195	3846277
4203610	3692955	4191067	4654924	4149269	3733864	4553903	4153527	4665134	4152028	3900574
4223550	3636545	4194013	4199827	4614083	3724878	4120796	4149085	4202701	4351394	3831888
3616241	4078212	3639754	3652050	3616653	3694136	3583371	3602797	3652312	3602949	3588781
3617669	4075293	3631465	3642136	3603282	3681392	3579286	3589715	3643045	3594671	3582647
4219997	3643874	4198637	4167359	4351003	3726336	4080964	4160524	4172810	4755239	3828830
3782094	3490879	3772151	3879414	3774255	3571862	3815212	3733988	3884986	3766209	3778338
4025756	3529146	3990845	4055595	4050400	3586403	4031795	3985507	4061389	4075319	3683461
4179771	3644538	4208275	4324245	4381059	3741659	4241925	4206711	4326398	4474861	3982059
4257386	3661517	4257100	4202527	4351917	3758842	4096225	4169824	4205588	4503441	3908643
3608715	4059982	3620616	3635458	3598107	3669539	3570519	3585640	3640633	3579731	3571156
4267149	3656623	4339836	4283733	4175593	3721898	4188134	4168104	4293144	4204664	3933116
4254542	3687375	4240928	4711651	4196799	3726908	4602034	4147154	4718063	4167178	3911672
3610433	4054421	3626623	3638307	3599087	3663625	3575124	3582396	3642446	3584998	3573020
4336885	3691102	4311469	4309056	4737741	3787047	4225490	4257173	4312269	4473734	3912506
4210414	3645421	4207256	4167060	4365684	3726730	4085479	4165419	4171999	4764395	3841626
4242001	3668608	4239551	4711927	4186937	3713202	4604191	4147332	4718412	4163438	3894589
4025771	3533831	3993575	4051363	4045476	3592173	4026913	3987674	4056682	4072517	3693450
4245639	3686677	4240129	4711598	4199498	3727106	4594521	4153396	4715951	4163096	3898708
4379580	3804841	4378581	4374706	4481531	3880477	4270833	4308925	4376761	4670317	3983202
3623283	4064541	3635276	3650221	3612756	3684729	3578117	3599710	3651640	3601405	3585572
4140822	3608266	4135891	4603966	4103706	3633055	4592464	4054429	4609842	4081079	3815752
3625180	4072945	3637083	3655483	3608345	3686767	3587371	3600752	3655586	3594574	3585091
4224470	3642368	4209879	4171737	4361030	3737759	4086193	4169934	4179456	4769822	3836905

4231877	3622136	4163715	4184094	4095556	3685174	4086553	4172598	4188872	4139565	3787374
4237028	3633087	4199360	4247404	4146918	3690346	4159547	4147258	4254363	4116925	3776291
3631949	4357899	3653775	3668565	3610424	3706231	3575854	3598799	3670201	3618533	3570052
4238091	3644409	4136003	4161145	4150324	3729205	4063182	4131394	4166848	4178340	3837682
3612219	4059192	3633388	3642990	3603099	3677913	3575440	3590089	3645043	3592657	3575348
4238436	3660413	4163789	4208659	4195577	3747041	4109961	4161955	4210891	4246093	3849878
4187107	3652829	4206871	4411759	4137261	3726875	4313701	4132898	4426560	4148474	3816302
4271527	3661589	4339092	4285284	4174319	3716095	4190226	4167329	4290477	4211102	3937449
3620754	4066833	3642425	3654326	3612574	3676755	3591168	3596124	3656058	3596762	3575645
4230480	3682314	4226919	4210031	4329767	3754703	4106737	4163089	4212775	4516691	3851823
4042650	3540176	3994345	4064865	4053229	3599018	4042952	3990672	4074053	4074221	3692916
4227842	3588219	4112699	4189676	4075259	3666435	4090192	4386379	4195411	4124157	3865908
3610156	4066019	3623999	3640513	3599138	3682782	3574933	3579987	3643087	3581497	3567246
3611298	4065298	3624995	3635027	3606194	3678819	3569610	3583383	3638760	3590109	3579156
4179065	3621523	4194765	4297765	4381138	3712692	4214992	4190876	4298685	4457516	3971895
3617548	4054717	3627658	3646971	3610143	3681367	3577175	3599763	3649815	3597985	3575806
4189883	3639856	4214779	4312006	4403060	3739423	4233996	4211626	4316598	4469337	3994592
4213465	3647069	4202919	4162678	4360674	3738659	4079202	4163221	4172487	4764964	3843684
3633341	4361325	3644516	3673105	3606688	3703776	3581662	3610819	3671471	3619385	3585480
4235810	3673910	4241827	4231570	4341562	3762377	4125721	4169153	4231387	4532782	3873657
4040432	3531610	3996391	4064364	4059259	3601893	4044444	3995715	4077033	4077886	3680951
3630133	4072725	3636850	3657168	3618179	3688622	3591557	3610557	3661220	3607013	3581616
3748150	3638408	3709483	3749682	3699644	3627084	3673962	3689871	3751336	3696124	3811196
4196979	3661339	4196863	4193297	4302119	3753702	4086243	4138216	4196162	4473114	3865439
4222923	3635417	4193890	4196216	4586204	3729756	4115677	4145958	4205352	4362443	3832703
4268462	3650140	4312326	4285256	4173564	3711539	4189663	4169994	4289836	4210473	3908546
3643880	4364740	3648732	3680190	3614783	3708620	3592490	3610573	3683264	3621239	3590565
4177314	3632013	4196834	4308752	4361241	3723696	4224283	4192180	4314458	4464076	3958806
4304386	3738204	4687829	4318250	4268573	3796380	4223355	4204339	4326234	4279683	3920312
4278458	3692810	4283151	4230821	4371781	3772116	4136708	4203853	4238282	4530859	3933491
4045264	3545394	4001879	4066731	4059332	3605941	4043499	3994176	4072835	4081923	3686353
3634432	4361883	3647399	3680422	3609705	3713443	3582458	3601271	3677034	3618035	3577370
4222322	3640475	4190012	4202636	4618548	3732367	4114262	4146412	4205365	4352094	3852010

4406547	3689067	4242246	4275942	4203824	3795714	4170596	4276372	4281345	4223787	3897241
4310299	3661137	4253800	4259833	4164566	3761915	4154216	4237935	4261804	4228464	3864924
4095623	3632541	4200794	4107194	4119760	3733937	4019530	4128437	4113243	4218540	3869367
4213853	3644417	4194134	4161185	4357416	3738214	4075058	4156975	4165813	4761851	3817815
4171468	3638030	4193043	4320075	4368113	3736167	4232942	4193813	4326340	4465370	3984386
4226088	3687577	4243086	4221115	4338795	3764986	4123835	4166440	4229051	4525403	3853874
4223322	3605768	4119696	4159773	4089676	3660020	4060394	4389426	4165492	4137958	3849652
4256155	3665136	4219366	4193102	4337321	3749091	4100686	4161566	4202167	4491116	3870437
3608556	4067656	3634619	3642029	3614380	3679070	3577557	3592716	3642268	3593434	3580638
4252107	3677027	4796295	4252062	4216062	3772753	4152404	4145573	4262492	4219131	3907124
4252402	3644045	4135303	4157908	4150058	3748409	4053011	4144523	4166050	4178481	3849868
4240700	3654551	4724098	4228683	4200630	3777165	4133794	4117794	4233413	4204187	3858835
-	3623522	4223389	4233211	4208885	3735220	4139275	4225952	4242393	4201739	3869438
0,914	-	3638437	3673069	3606774	3699582	3583459	3598669	3671256	3612368	3582427
0,979	0,914	-	4260932	4226309	3788919	4161052	4142881	4267456	4229200	3913413
0,980	0,913	0,981	-	4184052	3737024	4597140	4157168	4715264	4159180	3911902
0,980	0,913	0,979	0,981	-	3737720	4110997	4148776	4202129	4359332	3844346
0,915	0,929	0,916	0,915	0,916	-	3660558	3728065	3760354	3753197	3789133
0,980	0,913	0,981	1,000	0,981	0,916	-	4049692	4602920	4070242	3809662
0,981	0,914	0,980	0,981	0,980	0,917	0,981	-	4178176	4167153	3848397
0,980	0,913	0,981	1,000	0,981	0,916	1,000	0,982	-	4165161	3899193
0,979	0,914	0,980	0,980	0,994	0,917	0,980	0,980	0,979	-	3833362
0,923	0,911	0,922	0,926	0,924	0,916	0,928	0,925	0,926	0,924	-
0,981	0,915	0,979	0,981	0,981	0,916	0,981	0,999	0,981	0,981	0,924
0,980	0,914	0,981	0,982	0,984	0,917	0,982	0,983	0,982	0,983	0,926
0,931	0,909	0,933	0,933	0,932	0,911	0,933	0,932	0,933	0,932	0,919
0,979	0,914	0,979	0,981	1,000	0,917	0,981	0,980	0,981	0,995	0,926
0,980	0,914	0,979	0,981	1,000	0,917	0,981	0,981	0,981	0,995	0,925
0,981	0,914	0,981	0,982	0,984	0,918	0,982	0,983	0,982	0,983	0,926
0,983	0,914	0,979	0,979	0,979	0,915	0,979	0,981	0,979	0,977	0,923

60	3	61	51	57	6	69
4132496	4097759	3777828	4370961	4356803	4095769	4172821
3576603	3515384	3513456	3620900	3602377	3518744	3631115
4153896	4078729	3884139	4207849	4198517	4077358	4265140
4143243	4081825	3863313	4213743	4192985	4081431	4259205
4236304	4119961	3909394	4271581	4254472	4119818	4407792
3732980	3712020	4637152	3831538	3815684	3716904	3827914
4083225	4064940	3783582	4598282	4625586	4066248	4171423
4143436	4074055	3892830	4161313	4140947	4078008	4215485
4088344	4063026	3779804	4588961	4621035	4060433	4176059
3586294	3525309	3521526	3626088	3612763	3526833	3629081
3575796	3519517	3496960	3613075	3607487	3519826	3627349
4130056	4089211	3781729	4369233	4351173	4094618	4162004
3721408	3695775	4756740	3782809	3771345	3698732	3817298
4000421	4317817	3677406	4064058	4051836	4314330	4016133
4116987	4076202	3874228	4396274	4381861	4081275	4247326
4151497	4098412	3827491	4361894	4349128	4095982	4330894
3565735	3498796	3488036	3607179	3598316	3499851	3615691
4117823	4011650	3876762	4185007	4174637	4011275	4308409
4146227	4083705	3877005	4206725	4198526	4079569	4264702
3565444	3512985	3493645	3609414	3598938	3509959	3620612
4197432	4182589	3807983	4712211	4736172	4179451	4281133
4138590	4095540	3786954	4378306	4359008	4098957	4166651
4148509	4075329	3855313	4203200	4186368	4077823	4264044
4006604	4318430	3686367	4062564	4050395	4319942	4007421
4154911	4073800	3864235	4215834	4197799	4066576	4252427
4259209	4231806	3968073	4500395	4479414	4230965	4412252
3583716	3521244	3513316	3624158	3613433	3522854	3628742
4045317	4046559	3811337	4116262	4103960	4047054	4151794
3586997	3530715	3510311	3619820	3609979	3533776	3629814
4137820	4096009	3768042	4376022	4365169	4099806	4166996

4181001	4053598	3761168	4109014	4098091	4047776	4205672
4130109	4002136	3806340	4161799	4150917	4004214	4266810
3577343	3528125	3475795	3620999	3607295	3530630	3648900
4060175	4004453	3798837	4163927	4155843	3998451	4285380
3582969	3513798	3500925	3615319	3603647	3513019	3619834
4133185	4081212	3795130	4210992	4197128	4079333	4295762
4123027	4045593	3797193	4150119	4136061	4049719	4160873
4120669	4018286	3885447	4184280	4167741	4022429	4296767
3583432	3526764	3499967	3625449	3611966	3523287	3640643
4119003	4086104	3817671	4351848	4335590	4085627	4266997
4006895	4325496	3680335	4072709	4056721	4331210	4022808
4592943	4015213	3752802	4089483	4078239	4013306	4208941
3569779	3515836	3500713	3609635	3604169	3511829	3614497
3572224	3508819	3503062	3613220	3602286	3511440	3622823
4108458	4057727	3872693	4397129	4386108	4059802	4225497
3581879	3523478	3504637	3624568	3610855	3524935	3618138
4120530	4072786	3878406	4417578	4401528	4068370	4235408
4141344	4096420	3777817	4372552	4356051	4091819	4167597
3591327	3534976	3476807	3622290	3607579	3537853	3653149
4119251	4095894	3843297	4357397	4347153	4090460	4273979
4010208	4324306	3676595	4070479	4055571	4323620	4018349
3595183	3529921	3515482	3626501	3620722	3528248	3635579
3690536	3627651	3666418	3713249	3696902	3632570	3719428
4094630	4056374	3818976	4321350	4303430	4055406	4275344
4085276	4077234	3778113	4595330	4581971	4079128	4169777
4119648	4015174	3868050	4185881	4170249	4016605	4284058
3586416	3537418	3489089	3627125	3612709	3545381	3659338
4102190	4071900	3850476	4383950	4370338	4069114	4241074
4171969	4087063	3851594	4286903	4267571	4089041	4333035
4184780	4125087	3860528	4384779	4368229	4127863	4366268
4011041	4330660	3679647	4075455	4062103	4333001	4022769
3575989	3533019	3485943	3620533	3606981	3537804	3652702
4091615	4070147	3788922	4589504	4621655	4069529	4167991

4244024	4055818	3860478	4219308	4199464	4058089	4413302
4237380	4068913	3796969	4173089	4157373	4069044	4327715
4066570	4008017	3763420	4131476	4128263	4009424	4168720
4130293	4090129	3776483	4365706	4350434	4090244	4157787
4103262	4074391	3870144	4384375	4369259	4069353	4242439
4117054	4087923	3834598	4349291	4339434	4087757	4255006
4591010	4036351	3725842	4098619	4090618	4037778	4229045
4139611	4099825	3828356	4350666	4328923	4105444	4287044
3580783	3521178	3503701	3621724	3608436	3523062	3626304
4144519	4026108	3810536	4225037	4214785	4026077	4281080
4071610	3995425	3820191	4162110	4143880	3991207	4296380
4097355	4010406	3784421	4213699	4197427	4006040	4256141
4205163	4029404	3763674	4219477	4207612	4029656	4283735
3575560	3528493	3465669	3616737	3604852	3528876	3642927
4139711	4036448	3800035	4235120	4224955	4035110	4293515
4145917	4077211	3884254	4208858	4186936	4076690	4257785
4090786	4070990	3785273	4590952	4617470	4066498	4164868
3685321	3633774	3603106	3760116	3750257	3629820	3776707
4042007	4045382	3791453	4116470	4108444	4046794	4154131
4400165	4014972	3748665	4174416	4160044	4016866	4236571
4150691	4073157	3881779	4209931	4195504	4075555	4262881
4142013	4102147	3785043	4368173	4356552	4102489	4169443
3856819	3692096	3777493	3842050	3834087	3698754	3918817
-	4023214	3731931	4086264	4079488	4023647	4224085
0,982	-	3691658	4073270	4064899	4328690	4038873
0,933	0,933	-	3800296	3791689	3708982	3846404
0,981	0,983	0,933	-	4583058	4068184	4167111
0,981	0,984	0,933	1,000	-	4068536	4163205
0,982	1,000	0,934	0,984	0,984	-	4024399
0,980	0,980	0,932	0,979	0,979	0,980	-
