# Supplementary material for: Evolution of Stenotrophomonas maltophilia in Cystic Fibrosis Lung over Chronic Infection: A Genomic and Phenotypic Population Study
Source: Front Microbiol. 2017 Aug 28;8:1590. doi: 10.3389/fmicb.2017.01590 (PMC5581383; doi:10.3389/fmicb.2017.01590)
Supplement: Supplementary file 4 [file Table4.PDF]

Supplementary Table 4. Statistics about the sequence status of the genes mutL, mutS and uvrD, known to be involved in the hypermutable phenotype

	Alignment Length (bp)	# variable sites	Nucleotide diversity per site (Pi)	Tajima's D
mutL	1908	328	0,03	0,36 NS**
mutS	2685	375	0,03	0,26 NS
uvrD*	2329	307	0,02	-0,02 NS

*isolates 52 and 93 were removed from the alignment because the gene was fragmented

**Not Significant
