# Supplementary material for: Evolution of Stenotrophomonas maltophilia in Cystic Fibrosis Lung over Chronic Infection: A Genomic and Phenotypic Population Study
Source: Front Microbiol. 2017 Aug 28;8:1590. doi: 10.3389/fmicb.2017.01590 (PMC5581383; doi:10.3389/fmicb.2017.01590)
Supplement: Supplementary file 5 [file Table5.pdf]

Supplementary Table 5. Biofilm formation by *S. maltophilia* strains belonging to selected STs over 12-year period from 10 CF patients.

ST [a] (n)	No. (%) of strains belonging to the following biofilm producer classes[b]:				*p* -value (Fisher's exact test):	
	Non-producer	Weak	Moderate	Strong	within ST	*vs* other STs
5 (20)	1 (5)	3 (15)	9 (45)	7 (35)	0.004 (M vs NP)<br>0.048 (M vs W)	0.012 (S, vs 1007)
91 (11)	6 (54.5)	1 (9.1)	2 (18.2)	2 (18.2)	NS[c]	NS
179 (7)	0	2 (28.6)	1 (14.3)	4 (57.1)	NS	0.004 (S, vs 1007)<br>0.025 (S, vs 1008)
184 (15)	9 (60)	2 (13.3)	4 (26.6)	0	<0.001 (NP vs S)<br>0.020 (NP vs W)	0.007 (NP, vs 1008)<br>0.016 (NP, vs 1002)<br><0.001 (NP, vs 5)
185 (8)	0	5 (62.5)	3 (37.5)	0	0.025 (W vs NP)	0.025 (W, vs 1007)<br>0.040 (W, vs 91)<br>0.022 (W, vs 5)

[a] ST, sequence type.

[b] Biofilm biomass values were interpreted according to the following biofilm producer classes (see reference 3, supplementary materials and methods): OD ≤ ODc = non producer (NP); ODc < OD ≤ 2xODc = weak producer (W); 2xODc < OD ≤ 4xODc = moderate producer (M); OD> 4xODc = strong producer (S), where ODc is OD_{492} of control (not inoculated) wells.

[c] NS, not significant.
