# Supplementary material for: Evolution of Stenotrophomonas maltophilia in Cystic Fibrosis Lung over Chronic Infection: A Genomic and Phenotypic Population Study
Source: Front Microbiol. 2017 Aug 28;8:1590. doi: 10.3389/fmicb.2017.01590 (PMC5581383; doi:10.3389/fmicb.2017.01590)
Supplement: Supplementary file 6 [file Table6.PDF]

**Supplementary Table 6.** Mutation frequency of *S. maltophilia* strains collected over 12-year period from 10 CF patients.

| **ST[b] (n)** | **No. (%) of strains belonging to the following mutation frequency classes[a]:** | | | | ***p*-value (Fisher's exact test):** | |
|---|---|---|---|---|---|---|
| | **Hypo (H)** | **Normo (N)** | **Weak (W)** | **Strong (S)** | **within ST** | **among STs** |
| 5 (20) | 1 (5) | 0 | 13 (65) | 6 (30) | <0.0001 (W vs H, N) | |
| 91 (11) | 0 | 1 (9.1) | 10 (90.9) | 0 | <0.0001 (W vs H, N, S) | |
| 179 (7) | 0 | 4 (57.1) | 3 (42.9) | 0 | NS[c] | <0.05 (N: 179 vs 5, 91, 184) <0.0001 (W: 184 vs 5, 91, 179) <0.001 (S: 184 vs 5, 91, 179, 185) |
| 184 (15) | 0 | 1 (6.6) | 0 | 14 (93.4) | <0.0001 (S vs H, N, W) | |
| 185 (8) | 0 | 1 (12.5) | 5 (62.5) | 2 (25) | <0.05 (W vs H) | |

[a] Strains were classified into four categories, based on mutation frequency (f) (see reference 6, Supplementary Materials and Methods): hypo-mutators (H) ($f \le 8 \times 10^{-9}$), normo-mutators (N) ($8 \times 10^{-9} < f < 4 \times 10^{-8}$), weak-mutators (W) ($4 \times 10^{-8} \le f < 4 \times 10^{-7}$), and strong-mutators (S) ($f \ge 4 \times 10^{-7}$).

[b] ST, sequence type.

[c] NS, not significant.
