# Supplementary material for: Evolution of Stenotrophomonas maltophilia in Cystic Fibrosis Lung over Chronic Infection: A Genomic and Phenotypic Population Study
Source: Front Microbiol. 2017 Aug 28;8:1590. doi: 10.3389/fmicb.2017.01590 (PMC5581383; doi:10.3389/fmicb.2017.01590)
Supplement: Supplementary file 7 [file Table7.PDF]

		MIC (µg/ml) values for:									
strain ID	ST	P/T	LVX	AK	SXT	MIN	T/C	CHL	CIP	CAZ	DOX
ZC 2004	178	≥256	0,75	8	0,064	1	≥256	4	4	≥256	S
ZC 2005	5	≥256	0,75	12	0,125	0,19	≥256	4	3	≥256	S
ZC 2006	179	≥256	1	≥256	0,094	1	≥256	8	4	≥256	S
ZC 2007	179	≥256	1	≥256	0,19	1	≥256	6	4	≥256	S
ZC 2008	179	≥256	1	≥256	0,125	0,75	≥256	4	3	≥256	S
ZC 2009	179	≥256	1	≥256	0,094	0,5	≥256	4	4	≥256	S
ZC 2010	5	≥256	1	≥256	0,094	0,5	≥256	4	3	≥256	S
ZC 2011	5	≥256	≥32	≥256	0,5	4	≥256	48	≥32	≥256	S
ZC 2012-1	5	≥256	6	≥256	0,75	0,75	≥256	96	≥32	≥256	S
ZC 2012-2	179	≥256	0,5	≥256	0,5	0,5	≥256	3	4	≥256	S
ZC 2013-1	5	16	12	≥256	0,75	1	1	96	≥32	4	S
ZC 2013-2	179	≥256	2	≥256	0,25	0,75	≥256	6	4	≥256	S
ZC 2014	179	≥256	3	≥256	0,25	0,75	≥256	4	8	≥256	S
CV 2003-1	180	≥256	0,19	≥256	0,064	0,38	≥256	32	0,75	≥256	S
CV 2003-2	5	≥256	≥32	4	≥32	2	≥256	32	≥32	≥256	S
CV 2005	5	≥256	≥32	32	≥32	4	≥256	32	≥32	≥256	S
CV 2006-1	5	≥256	≥32	32	≥32	2	≥256	24	≥32	≥256	S
CV 2006-2	5	≥256	≥32	≥256	≥32	4	≥256	32	≥32	≥256	S
CV 2007	5	≥256	0,38	≥256	0,125	0,38	≥256	4	3	≥256	S
CV 2008	5	≥256	≥32	≥256	≥32	2	≥256	4	≥32	≥256	S
CV 2010	5	≥256	0,19	≥256	0,064	0,5	≥256	1	6	≥256	S
CV 2013	29	32	1,5	12	0,38	0,5	6	8	3	0,75	S
MS 2003-1	181	≥256	0,19	32	0,125	0,19	6	4	0,75	≥256	S
MS 2003-2	181	≥256	0,125	8	0,125	0,19	≥256	2	0,5	≥256	S
MS 2006	5	24	0,75	4	0,19	1	4	8	1,5	0,75	S
MS 2008	182	≥256	0,25	4	0,094	0,5	≥256	32	0,32	≥256	S
MS 2009	183	≥256	0,25	12	0,047	0,5		32	0,5	≥256	S
MS 2010	4	48	0,5	≥256	0,125	0,25	2	6	1	≥256	S
MS 2011	4	≥256	0,38	12	0,25	0,25	2	4	0,75	96	S
MS 2012	4	≥256	0,5	32	0,125	0,25	8	4	1	96	S
MS 2013	92	≥256	0,38	16	0,064	0,25	3	6	0,5	≥256	S
MS 2014	5	≥256	0,75	≥256	0,125	1	12	12	1	≥256	S
TG 2004	91	≥256	0,75	6	0,094	0,19	64	4	1	24	S
TG 2005	184	≥256	0,25	≥256	0,064	0,38	≥256	4	6	≥256	S
TG 2006-1	184	≥256	1	≥256	0,125	0,5	≥256	16	6	≥256	S
TG 2006-2	184	≥256	≥32	≥256	0,094	0,5	≥256	12	≥32	≥256	S
TG 2007-1	184	≥256	1,5	≥256	0,064	1,5	≥256	24	≥32	≥256	S
TG 2007-2	184	≥256	4	≥256	0,125	2	≥256	24	6	≥256	S
TG 2008-1	184	≥256	≥32	≥256	0,064	0,5	≥256	256	≥32	≥256	S
TG 2008-2	184	≥256	1	≥256	0,064	0,38	≥256	6	3	≥256	S
TG 2009-1	184	≥256	0,25	≥256	0,38	0,25	≥256	8	2	≥256	S
TG 2009-2	184	≥256	1,5	≥256	0,094	1	≥256	12	3	≥256	S
TG 2012-1	184	≥256	1,5	≥256	0,047	0,38	≥256	3	2	≥256	S
TG 2012-2	184	≥256	1,5	48	0,047	0,75	≥256	4	6	≥256	S
TG 2013	184	≥256	3	32	0,047	0,75	≥256	16	6	≥256	S
TG 2014-1	184	≥256	0,5	≥256	0,047	0,25	≥256	6	1,5	≥256	S
TG 2014-2	184	≥256	0,064	8	0,016	0,023	≥256	2	0,25	≥256	S
FMa 2005-1	185	≥256	0,75	≥256	0,125	0,5	32	4	4	≥256	S
FMa 2005-2	185	≥256	0,75	≥256	0,125	0,5	32	4	3	≥256	S
FMa 2007	2	16	0,38	12	0,125	0,25	1	4	0,5	8	S
FMa 2008	185	96	0,75	≥256	0,125	0,5	48	32	1,5	≥256	S
FMa 2009	185	≥256	0,5	≥256	0,19	0,5	24	32	1,5	≥256	S
FMa 2010-1	185	≥256	0,5	≥256	0,19	0,5	32	32	1	≥256	S
FMa 2010-2	185	≥256	0,75	≥256	0,19	0,75	≥256	64	2	≥256	S
FMa 2011	185	≥256	0,75	≥256	0,19	0,5	32	48	2	≥256	S
FMa 2012	185	≥256	0,75	≥256	0,125	0,75	32	48	1,5	≥256	S
AV 2005	2	16	0,25	≥256	≥32	4	1	≥256	0,5	≥256	R
AV 2006	2	≥256	0,38	≥256	0,064	8	8	64	1	≥256	S
AV 2009	183	≥256	1,5	64	0,125	1,5	4	32		≥256	S
AV 2010	2	≥256	0,25	≥256	0,032	0,094	4	2	0,5	≥256	S
AV 2011	5	≥256	0,5	≥256	0,032	0,25		2	1	≥256	S
AV 2012-1	5	≥256	2	64	0,125	2	6	16	4	≥256	S
AV 2012-2	5	6	4	≥256	0,125	2	1	32	8	1,5	S
AV 2013	5	≥256	1,5	≥256	0,125	1	4	32	4	≥256	S
GC 2008 col.A	91	12	0,75	16	0,125	0,5	1	8	3	3	S
GC 2008 col.B	91										
GC 2009	162	≥256	1,5	1,5	0,125	0,38	2	4	3	≥256	S
GC 2010	91	8	0,38	4	0,064	0,19	0,75	3	0,75	16	S
GC 2011	91	8	1	6	0,094	0,38	0,75	4	2	3	S
GC 2012-1	91	≥256	1	12	0,094	0,5	4	4	≥32	≥256	S
GC 2012-2	91	≥256	≥32	4	0,125	0,125	≥256	4	≥32	≥256	S
GC 2013-1	91	≥256	≥32	≥256	0,047	0,25	≥256	32	16	≥256	S
GC 2013-2	91	≥256	≥32	≥256	0,19	0,25	≥256	12	≥32	≥256	S
GC 2014-1	91	≥256	12	6	0,25	2	≥256	24	≥32	≥256	S
GC 2014-2	91	≥256	1	4	0,094	0,5	≥256	6	2	≥256	S
MC 2009	5	16	1	32	0,75	2	0,75	6	4	6	R
MC 2010-1	183	12	0,25	≥256	0,094	0,38	1	1	1	8	S
MC 2010-2	184	24	1	≥256	0,75	2	1	16	4	4	R
MC 2011-1	187	8	0,19	24	0,25	0,75	0,75	2	0,5	1,5	R
MC 2011-2	186	32	0,38	≥256	0,25	1,5	3	8	1,5	24	R
MC 2012	186	≥256	0,19	≥256	0,38	0,75	32	6	0,5	≥256	S
MC 2013	186	≥256	0,25	≥256	0,5	1	32	8	0,75	≥256	S
MC 2014	186	≥256	0,75	≥256	0,38	1	48	4	2	≥256	S
BB 2010	139	≥256	0,5	16	0,125	0,25	≥256	4	0,5	≥256	S
BB 2011	188	≥256	1	4	0,094	0,5	8	12	3	≥256	S
BB 2012	188	≥256	1	8	0,094	0,75	32	12	3	≥256	S
BB 2013	139	≥256	0,5	≥256	0,125	0,5	≥256	4	1	≥256	S
San G 2010	188	6	0,19	4	0,125	0,25	0,5	4	0,5	1,5	S
San G 2011	188	12	0,38	6	0,125	0,25	2	4	0,75	≥256	S
San G 2012	5	16	0,75	6	0,125	0,5	1,5	8	1,5	≥256	S
San G 2013	84	≥256	0,38	4	0,125	0,19	24	6	1	≥256	S

Supplementary Table 7. In vitro susceptibility to nine antibiotics by 91 *S. maltophilia* strains isolated over 12-year period from 10 CF patients. MIC values were stratified on ST and interpreted according to CLSI-suggested breakpoints. Red box indicates «resistance», orange box indicates «intermediate susceptibility», and green box indicates «susceptibility». TZP, piperacillin/tazobactam; LVX, levofloxacin; AMK, amikacin; SXT, cotrimoxazole; MIN, minocyclin; TIM, ticarcillin/clavulanate; CHL, chloramphenicol; CIP, ciprofloxacin; CAZ, ceftazidime; DOX, doxicycline. Doxicycline was tested by Kirby-Bauer technique (S, susceptibility; R, resistance).
