# Supplementary material for: Evolution of Stenotrophomonas maltophilia in Cystic Fibrosis Lung over Chronic Infection: A Genomic and Phenotypic Population Study
Source: Front Microbiol. 2017 Aug 28;8:1590. doi: 10.3389/fmicb.2017.01590 (PMC5581383; doi:10.3389/fmicb.2017.01590)
Supplement: Supplementary file 8 [file Table8.pdf]

Supplementary Table 8. *In vitro* susceptibility of *S. maltophilia* strains, isolated from 10 CF patients over 12-year period, belonging to selected STs. MIC values were stratified on ST and interpreted according to CLSI-suggested breakpoints. Drug abbreviations: TZP, piperacillin-tazobactam; LVX, levofloxacin; AMK, amikacin; SXT, trimethoprim-sulfamethoxazole; MIN, minocycline; TIM, ticarcillin-clavulanic acid; CHL, chloramphenicol; CIP, ciprofloxacin; CAZ, ceftazidime; DOX, doxicycline.

ST[b] (n)	No. (%) of strains resistant[a] to:									
	**TZP**	**LVX**	**AMK**	**SXT**	**MIN**	**TIM**	**CHL**	**CIP**	**CAZ**	**DOX**
**5 (20)**	16 (80)	9 (45)	16 (80)	5 (25)	0 (0)	11 (55)§	11 (55)	18 (90)	16 (80)	1 (0.5)
**91 (10)**	7 (70)	4 (40)	2 (20)°	0 (0)	0 (0)	6 (60)	3 (30)	8 (80)	8 (80)	0 (0)
**179 (7)**	7 (100)	6 (85.7)*	7 (100)	0 (0)	0 (0)	7 (100)	0 (0)^	7 (100)	7 (100)	0 (0)
**184 (15)**	15 (100)	4 (26.6)	14 (93.3)	0 (0)	0 (0)	14 (93.3)	8 (53.3)	14 (93.3)	14 (93.3)	1 (0.5)
**185 (8)**	8 (100)	0 (0)	8 (100)	0 (0)	0 (0)	8 (100)	6 (75)	7 (87.5)	8 (100)	0 (0)

[a] Strains showing "intermediate susceptibility" were considered as "resistant".

[b] ST, sequence type.

* LVX: ST179 vs ST184 and ST185; $p<0.05$, chi-square test.

^ CHL: ST179 vs ST5, ST184, and ST185; $p<0.05$, chi-square test.

° AMK: ST91 vs other STs; $p<0.01$, chi-square test.

§ TIM: ST5 vs ST179, ST184 and ST185; $p<0.05$, chi-square test.
