# Supplementary material for: Evolution of Stenotrophomonas maltophilia in Cystic Fibrosis Lung over Chronic Infection: A Genomic and Phenotypic Population Study
Source: Front Microbiol. 2017 Aug 28;8:1590. doi: 10.3389/fmicb.2017.01590 (PMC5581383; doi:10.3389/fmicb.2017.01590)
Supplement: Supplementary file 9 [file Table9.PDF]

Spearman r-values / p-values	biofilm biomass	growth rate	swimming	twitching	swarming	mutation frequency	virulence score (LD_{50}+LD_{100})
biofilm biomass		0,1203	0,0416	0,1738	0,2935	0,2294	0,2503
growth rate	0,6134		0,3511	0,2125	0,2470	-0,0985	0,1349
swimming	0,8617	0,1290		**0,7811**	0,3924	-0,1366	-0,0265
twitching	0,4636	0,3683	**<0,0001**		**0,6820**	-0,3144	-0,0994
swarming	0,2092	0,2938	0,0870	**0,0009**		-0,3273	0,0179
mutation frequency	0,3306	0,6794	0,5657	0,1771	0,1590		0,1392
virulence score (LD_{50}+LD_{100})	0,2871	0,5706	0,9118	0,6768	0,9402	0,5583	

ST5

Spearman r-values / p-values	biofilm biomass	growth rate	swimming	twitching	swarming	mutation frequency	virulence score (LD_{50}+LD_{100})
biofilm biomass		0,3091	-0,0506	-0,5230	0,1223	-0,4419	0,2700
growth rate	0,3560		**-0,7219**	0,1651	-0,2592	0,1367	-0,0092
swimming	0,8531	**0,0121**		-0,1044	0,0544	-0,4286	0,0972
twitching	0,0954	0,6258	0,7056		-0,1851	**0,6897**	-0,2217
swarming	0,7234	0,3163	0,8784	0,4354		-0,0417	-0,4358
mutation frequency	0,1693	0,6873	0,1780	**0,0223**	0,7392		0,0115
virulence score (LD_{50}+LD_{100})	0,4189	0,9617	0,7738	0,4579	0,1111	0,9756	

ST91

Spearman r-values / p-values	biofilm biomass	growth rate	swimming	twitching	swarming	mutation frequency	virulence score (LD_{50}+LD_{100})
biofilm biomass		**-0,7857**	**0,8214**	**0,7857**	0,1871	0,1429	0,1442
growth rate	**0,0480**		**-0,7857**	**-0,8214**	-0,2433	-0,3214	-0,1802
swimming	**0,0341**	**0,0480**		**0,9643**	0,5426	0,0000	0,2342
twitching	**0,0480**	**0,0341**	**0,0028**		0,4117	0,0357	0,0541
swarming	0,6905	0,5238	0,2238	0,3762		0,3181	0,1416
mutation frequency	0,7825	0,4976	1,0365	0,9635	0,4952		-0,4865
virulence score (LD_{50}+LD_{100})	0,7571	0,6667	0,6198	0,9190	0,7429	0,2579	

ST179

Spearman r-values / p-values	biofilm biomass	growth rate	swimming	twitching	swarming	mutation frequency	virulence score (LD_{50}+LD_{100})
biofilm biomass		-0,1571	0,3743	0,4370	0,0978	-0,0824	-0,0910
growth rate	0,5756		0,2535	0,0227	**0,5441**	**-0,5663**	-0,2499
swimming	0,1905	0,3143		0,3134	0,4340	**-0,2545**	**-0,3438**
twitching	0,1047	0,9005	0,2571		0,3044	-0,3493	-0,2647
swarming	0,7295	**0,0373**	0,1048	0,2738		**-0,5501**	-0,1127
mutation frequency	0,7622	**0,0290**	**<0,0001**	0,1357	**0,0055**		0,1944
virulence score (LD_{50}+LD_{100})	0,5352	0,2259	**<0,0001**	0,0840	0,2547	0,4837	

ST184

Spearman r-values / p-values	biofilm biomass	growth rate	swimming	twitching	swarming	mutation frequency	virulence score (LD_{50}+LD_{100})
biofilm biomass		0,2143	0,5455	-0,0634	0,3273	-0,2156	nd
growth rate	0,6191		0,4728	-0,3678	0,2182	-0,3353	nd
swimming	0,1829	0,2548		-0,2712	0,6667	-0,3476	nd
twitching	0,7702	0,2893	0,3905		0,4069	-0,1085	nd
swarming	0,5357	0,7500	0,1429	0,3929		-0,2195	nd
mutation frequency	0,5886	0,4000	0,3480	0,6976	0,2500		nd
virulence score (LD_{50}+LD_{100})	nd	nd	nd	nd	nd	nd	

ST185

Supplementary Table 9. Correlation between phenotypic traits evaluated in *S. maltophila* strains according to each ST. Correlation was evaluated by calculating Spearman r correlation coefficient. Positive values are suggestive for direct relationship, whereas negative values are suggested for inverse relationship. Significant Spearman r values, along with associated two-tailed *p*-values, are shown in bold. ND, not determined.
