# Supplementary material for: Evolution of Stenotrophomonas maltophilia in Cystic Fibrosis Lung over Chronic Infection: A Genomic and Phenotypic Population Study
Source: Front Microbiol. 2017 Aug 28;8:1590. doi: 10.3389/fmicb.2017.01590 (PMC5581383; doi:10.3389/fmicb.2017.01590)
Supplement: Supplementary file 10 [file Table10.PDF]

Spearman r-values / p-values	biofilm biomass	growth rate	swimming	twitching	swarming	mutation frequency	virulence score (LD_{50}+LD_{100})
biofilm biomass		**0,8214**	0,2703	-0,2433	-0,1442	-0,3214	0,3744
growth rate	**0,0341**		-0,1261	-0,2433	0,0721	-0,3571	0,5714
swimming	0,5579	0,7571		0,4154	-0,7364	0,3964	0,2088
twitching	0,5095	0,5095	0,3333		-0,4343	0,3555	-0,1445
swarming	0,7286	0,8873	0,0556	0,2714		-0,3424	-0,4970
mutation frequency	0,4976	0,4444	0,3833	0,4381	0,4262		0,1576
virulence score (LD_{50}+LD_{100})	0,4190	0,2000	0,6476	0,5143	0,1429	0,7619	

CV_5

Spearman r-values / p-values	biofilm biomass	growth rate	swimming	twitching	swarming	mutation frequency	virulence score (LD_{50}+LD_{100})
biofilm biomass		0,3455	-0,1030	-0,5636	0,0122	-0,4255	0,3476
growth rate	0,3304		**-0,6848**	0,1515	-0,0671	0,0851	-0,0183
swimming	0,7850	**0,0347**		-0,0909	-0,3171	-0,3891	0,3171
twitching	0,0963	0,6821	0,8113		0,0427	0,6322	-0,3598
swarming	0,9789	0,8430	0,3602	0,9114		0,3914	-0,5123
mutation frequency	0,2126	0,8182	0,2569	0,0553	0,2596		-0,2599
virulence score (LD_{50}+LD_{100})	0,3236	0,9522	0,3696	0,2968	0,1191	0,4416	

GC_91

Spearman r-values / p-values	biofilm biomass	growth rate	swimming	twitching	swarming	mutation frequency	virulence score (LD_{50}+LD_{100})
biofilm biomass		**-0,7857**	**0,8214**	**0,7857**	0,1871	0,1429	0,1442
growth rate	**0,0480**		**-0,7857**	**-0,8214**	-0,2433	-0,3214	-0,1802
swimming	**0,0341**	**0,0480**		**0,9643**	0,5426	0,0000	0,2342
twitching	**0,0480**	**0,0341**	**0,0028**		0,4117	0,0357	0,0541
swarming	0,6905	0,5238	0,2238	0,3762		0,3181	0,1416
mutation frequency	0,7825	0,4976	1,0365	0,9635	0,4952		-0,4865
virulence score (LD_{50}+LD_{100})	0,7571	0,6667	0,6198	0,9190	0,7429	0,2579	

ZC_179

Spearman r-values / p-values	biofilm biomass	growth rate	swimming	twitching	swarming	mutation frequency	virulence score (LD_{50}+LD_{100})
biofilm biomass		-0,5121	-0,3700	0,4013	-0,2594	0,1165	-0,0962
growth rate	0,0641		0,1838	-0,2492	**0,5699**	-0,4681	-0,1666
swimming	0,0841	0,5257		-0,0295	0,1434	-0,1789	-0,2329
twitching	0,1551	0,3787	0,6410		-0,1335	-0,1764	-0,0942
swarming	0,3508	**0,0360**	0,6202	0,6126		**-0,5322**	-0,1160
mutation frequency	0,6930	0,0938	0,3130	0,5333	**0,0482**		0,0821
virulence score (LD_{50}+LD_{100})	0,5792	0,4222	0,1000	0,5752	0,5013	0,7824	

TG_184

Spearman r-values / p-values	biofilm biomass	growth rate	swimming	twitching	swarming	mutation frequency	virulence score (LD_{50}+LD_{100})
biofilm biomass		0,2143	0,5455	-0,0634	0,3273	-0,2156	nd
growth rate	0,6191		0,4728	-0,3678	0,2182	-0,3353	nd
swimming	0,1829	0,2548		-0,2712	0,6667	-0,3476	nd
twitching	0,7702	0,2893	0,3905		0,4069	-0,1085	nd
swarming	0,5357	0,7500	0,1429	0,3929		-0,2195	nd
mutation frequency	0,5886	0,4000	0,3480	0,6976	0,2500		nd
virulence score (LD_{50}+LD_{100})	nd	nd	nd	nd	nd	nd	

FMa_185

Supplementary Table 10. Correlation between phenotypic traits evaluated in *S. maltophila* strains according to each «ST-patient» combination. Correlation was evaluated by calculating Spearman r correlation coefficient. Positive values are suggestive for direct relationship, whereas negative values are suggested for inverse relationship. Significant Spearman r values, along with associated two-tailed *p*-values, are shown in bold. ND, not determined.
