# Supplementary material for: Evolution of Stenotrophomonas maltophilia in Cystic Fibrosis Lung over Chronic Infection: A Genomic and Phenotypic Population Study
Source: Front Microbiol. 2017 Aug 28;8:1590. doi: 10.3389/fmicb.2017.01590 (PMC5581383; doi:10.3389/fmicb.2017.01590)
Supplement: Supplementary file 11 [file Table11.PDF]

Supplementary Table 11. Pagel's lambda value and corresponding p-value for each phenotype.

**Phenotype**	**Pagel's λ**	***p*-value***
Biofilm	0.47	<0.01
Growth rate	0.12	NS
Swimming	0.51	<0.01
Twitching	0.54	<0.01
Swarming	0.48	<0.01
Mutation frequency (MF)	0.26	<0.01
Log10(MF)	0.48	<0.01
In vivo virulence	0.33	<0.01
DOX	$6.61x10^{-5}$	NS
TZP	$6.61x10^{-5}$	NS
LVX	0.11	NS
AMK	0.51	<0.01
SXT	0.07	NS
MIN	$6.61x10^{-5}$	NS
TIM	0.41	<0.01
CHL	0.25	<0.01
CIP	0.55	<0.01
CAZ	$6.61x10^{-5}$	NS

* after 999 permutations.

Drug abbreviations: DOX, doxicycline; TZP, piperacillin-tazobactam; LVX, levofloxacin; AMK, amikacin; SXT, trimethoprim-sulfamethoxazole; MIN, minocycline; TIM, ticarcillin-clavulanic acid; CHL, chloramphenicol; CIP, ciprofloxacin; CAZ, ceftazidime.

NS: not significant.
