# Supplementary material for: Evolution of Stenotrophomonas maltophilia in Cystic Fibrosis Lung over Chronic Infection: A Genomic and Phenotypic Population Study
Source: Front Microbiol. 2017 Aug 28;8:1590. doi: 10.3389/fmicb.2017.01590 (PMC5581383; doi:10.3389/fmicb.2017.01590)
Supplement: Supplementary file 12 [file Image1.PDF]

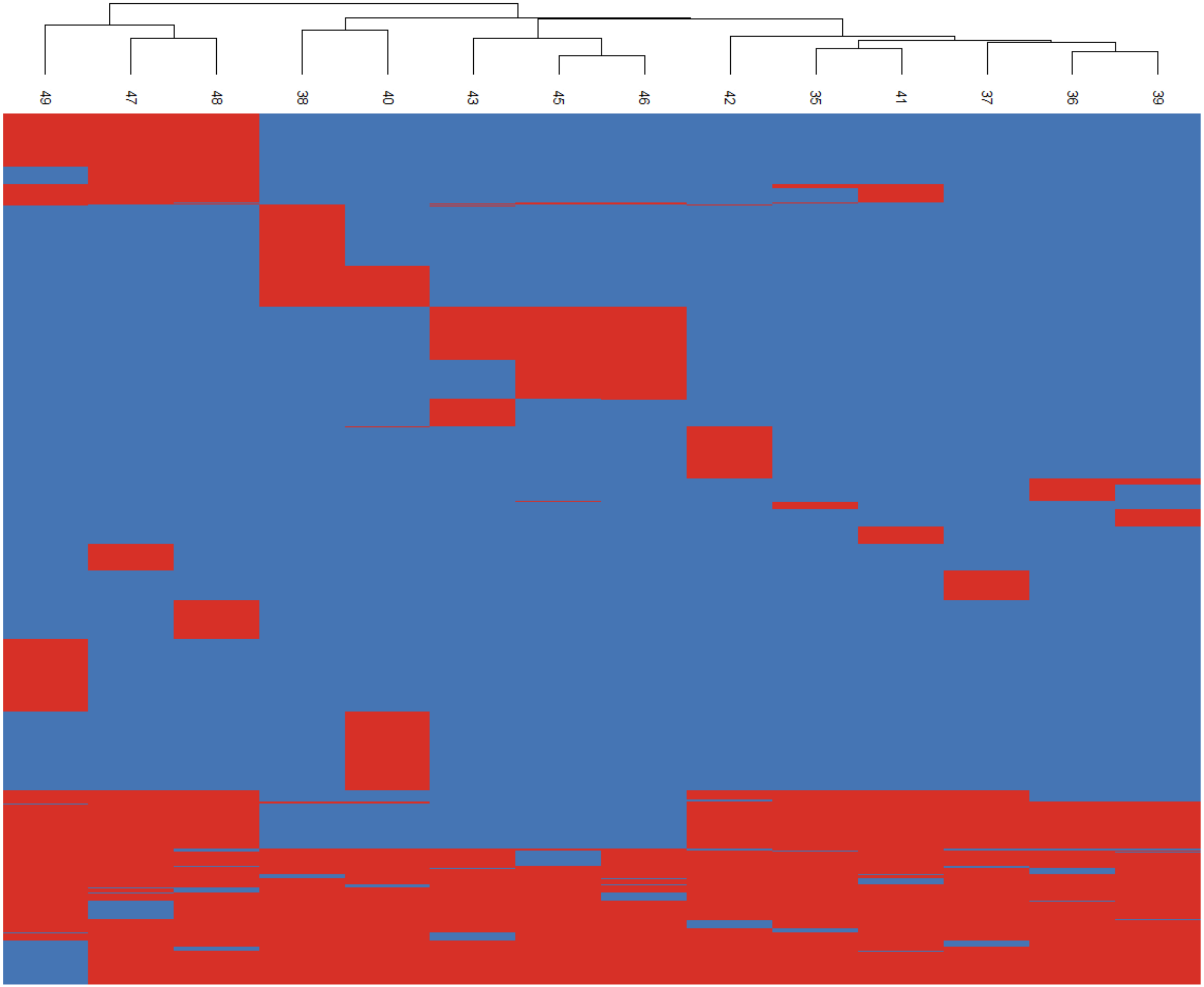

Supplementary Figure 1. Binary heatmap of SNPs occurring in the patient-ST combination TG_184. The clustering pattern does not reflect the time line as in the case of GC_91.
