# Supplementary material for: Evolution of Stenotrophomonas maltophilia in Cystic Fibrosis Lung over Chronic Infection: A Genomic and Phenotypic Population Study
Source: Front Microbiol. 2017 Aug 28;8:1590. doi: 10.3389/fmicb.2017.01590 (PMC5581383; doi:10.3389/fmicb.2017.01590)
Supplement: Supplementary file 14 [file Image3.PDF]

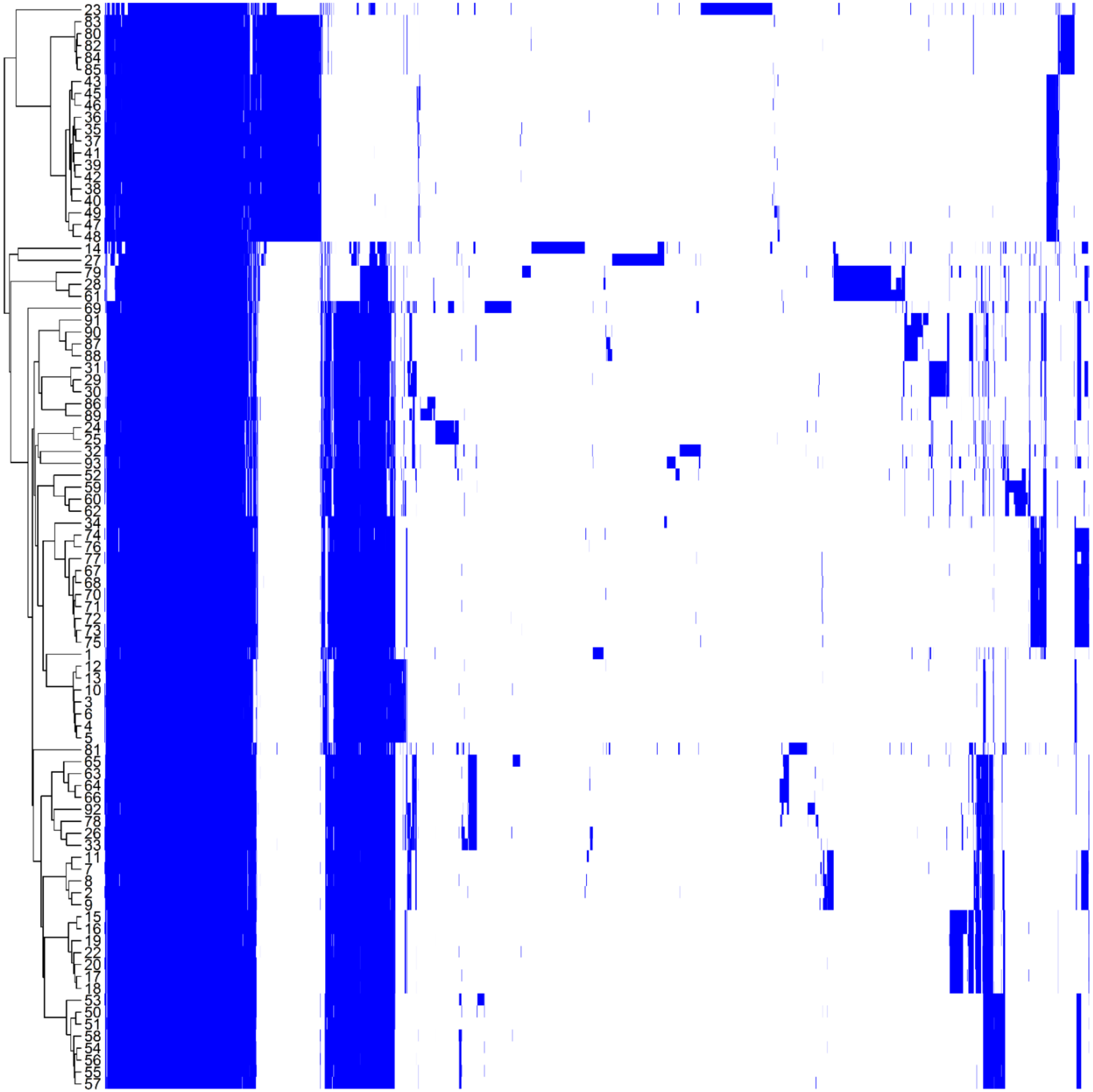

Supplementary Figure 3. Phylogenomic tree based on the gene presence/absence. The clustering pattern is in agreement with the tree shown in Figure 2. The two major lineages of isolates (one containing TG_184 and MC_186, and the other one containing most of the known STs) are also characterized by a peculiar gene content. Other minor lineages (isolates 14-27 and 28-71-69) display also a distinctive genomic fingerprint.
