# Supplementary material for: Evolution of Stenotrophomonas maltophilia in Cystic Fibrosis Lung over Chronic Infection: A Genomic and Phenotypic Population Study
Source: Front Microbiol. 2017 Aug 28;8:1590. doi: 10.3389/fmicb.2017.01590 (PMC5581383; doi:10.3389/fmicb.2017.01590)
Supplement: Supplementary file 15 [file Image4.PDF]

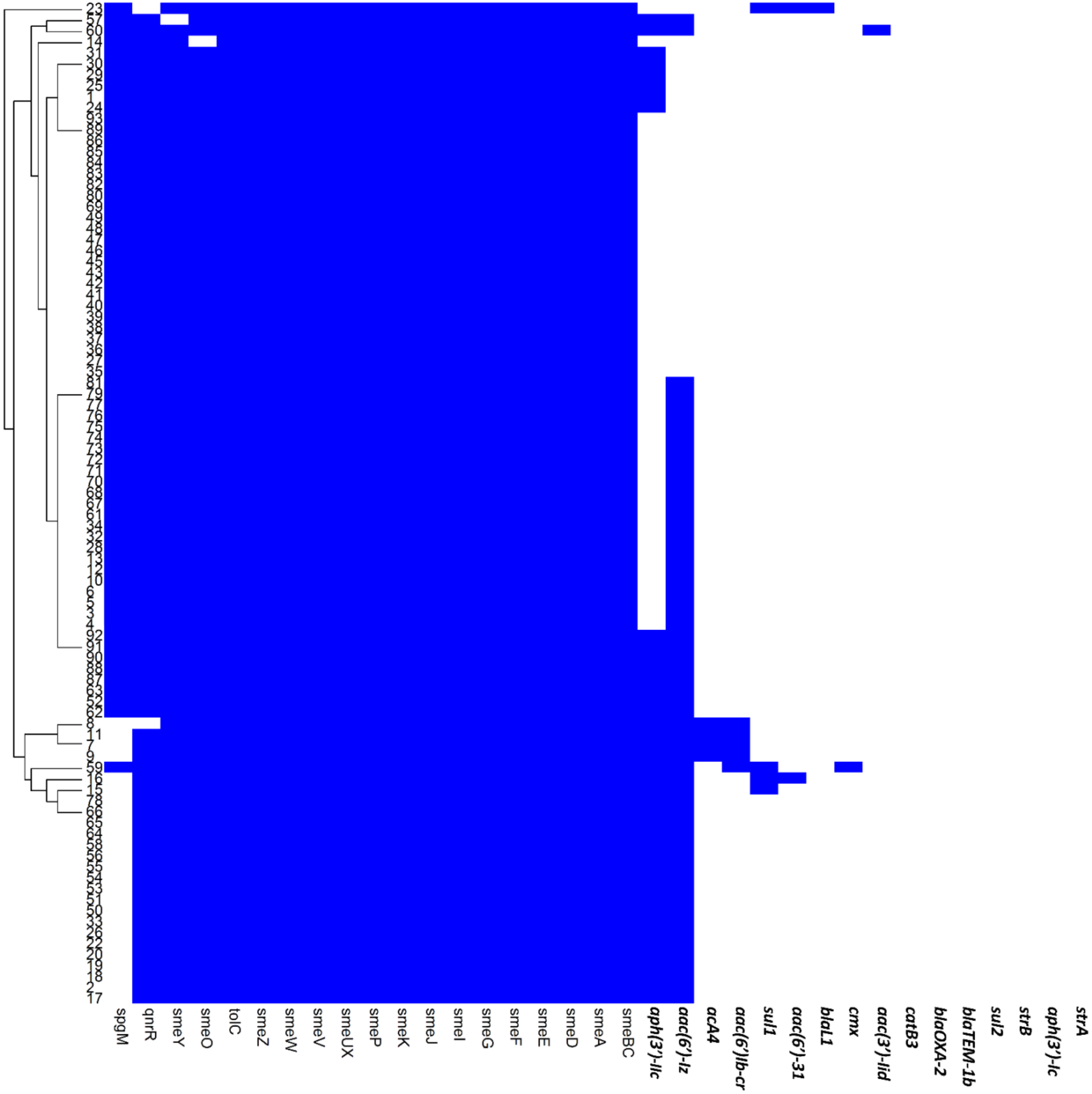

Supplementary Figure 4a. Presence/absence heatmap for antibiotic-resistance genes of *S. maltophilia* strains collected over 12-year period from 10 CF patients. Blue color indicates gene's presence. Gene names in italic bold were searched using ResFinder, the others were identified using BLAST.

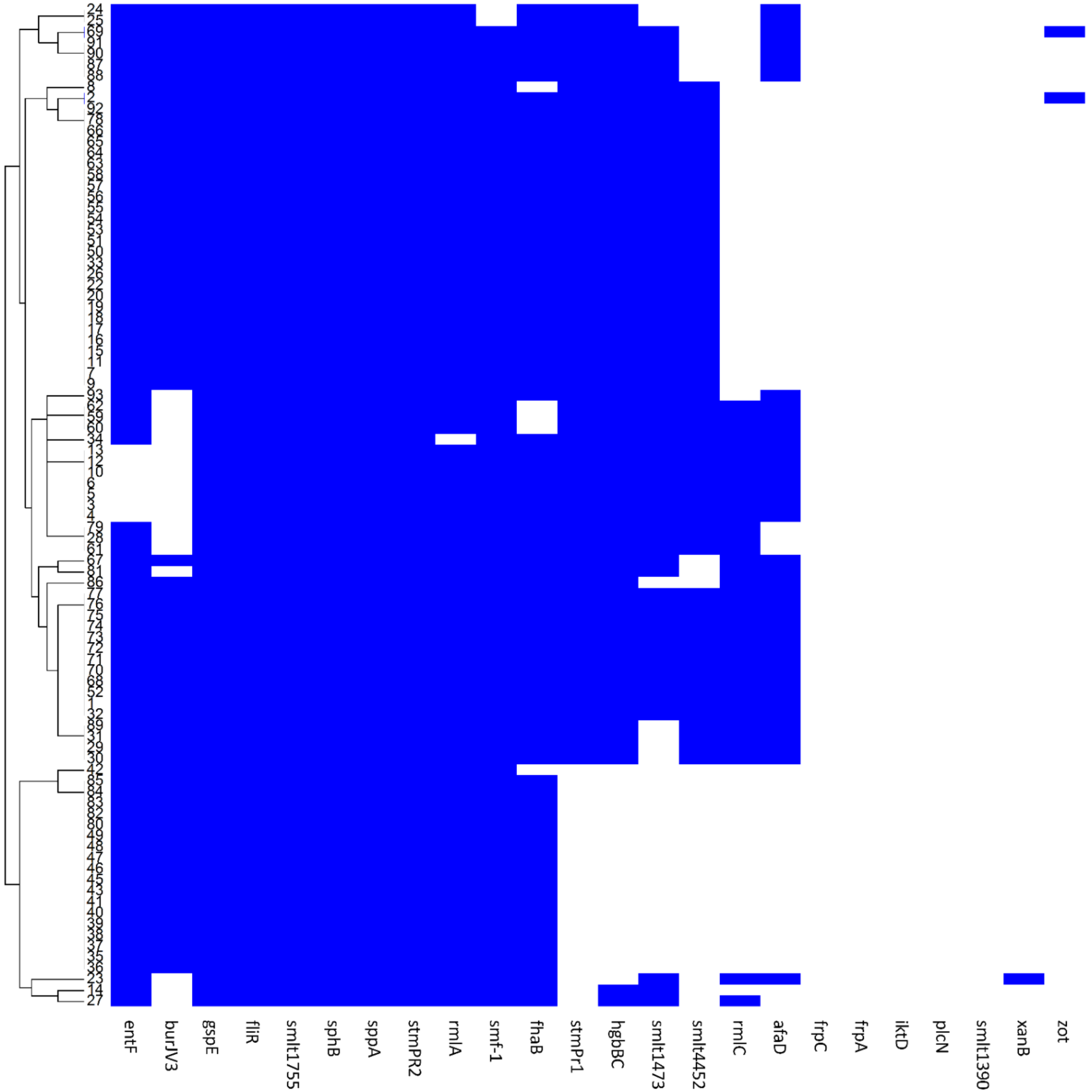

Supplementary Figure 4b. Presence/absence heatmap for virulence genes of *S. maltophilia* strains collected over 12-year period from 10 CF patients. Blue color indicates gene’s presence.
