# Supplementary material for: Evolution of Stenotrophomonas maltophilia in Cystic Fibrosis Lung over Chronic Infection: A Genomic and Phenotypic Population Study
Source: Front Microbiol. 2017 Aug 28;8:1590. doi: 10.3389/fmicb.2017.01590 (PMC5581383; doi:10.3389/fmicb.2017.01590)
Supplement: Supplementary file 16 [file Image5.PDF]

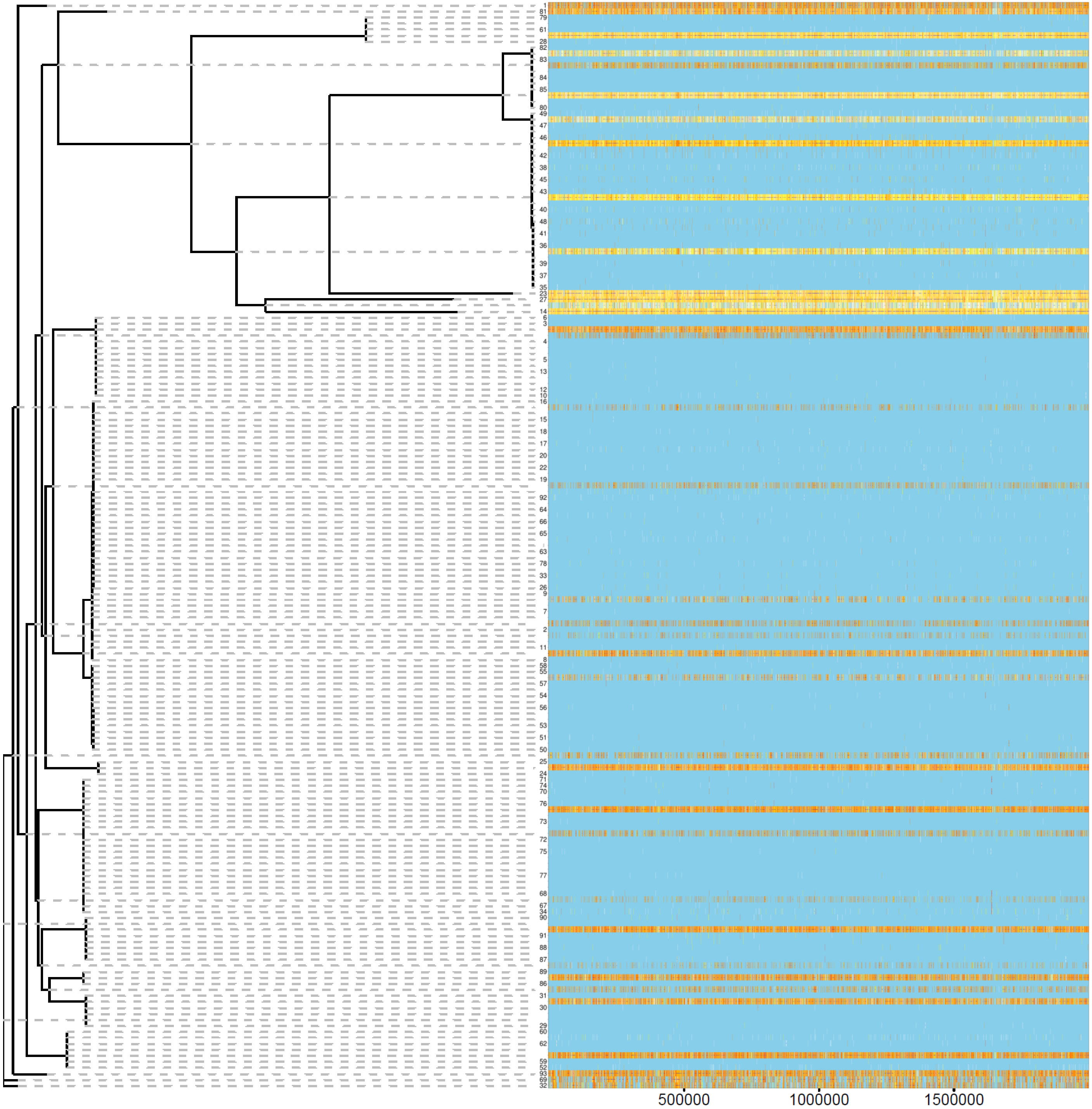

Supplementary Figure 5. Phylogeny based on the alignment of the core genome (including branch length), accounting for recombination. The ancestral state of each character was calculated by the software ClonalFrameML and is displayed along with each tip of the tree. The right panel represents the whole length of the alignment on the x-axes. Homologous, invariant position are showed as light blue. White to red means an increasing probability of homoplasic character at a given position.
