# Supplementary material for: Evolution of Stenotrophomonas maltophilia in Cystic Fibrosis Lung over Chronic Infection: A Genomic and Phenotypic Population Study
Source: Front Microbiol. 2017 Aug 28;8:1590. doi: 10.3389/fmicb.2017.01590 (PMC5581383; doi:10.3389/fmicb.2017.01590)
Supplement: Supplementary file 17 [file Image6.PDF]

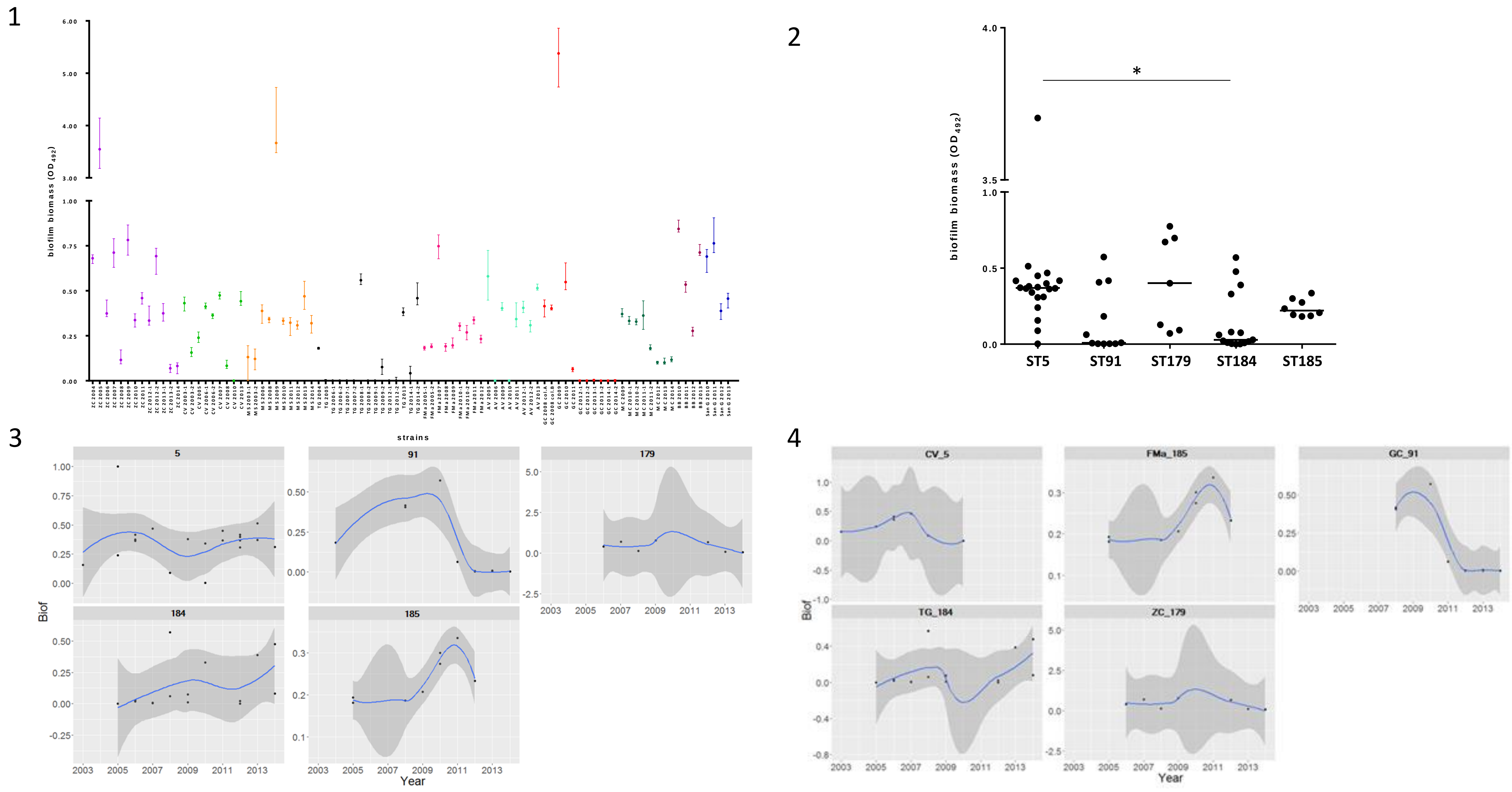

Supplementary Figure 6a. Biofilm formation by *S. maltophilia* strains collected over 12-year period from 10 CF patients. A) Overall biofilm formation. Results are medians with interquartile range. B) Biofilm formation values stratified on selected ST. Horizontal lines are median values. * *p*< 0.05, Kruskall-Wallis + Dunn's multiple comparison post-test. C) Temporal trend of biofilm formation in selected ST. D) Temporal trend of biofilm formation in each «ST-patient» combination. The statistical significance of temporal trends was assessed by linear regression.

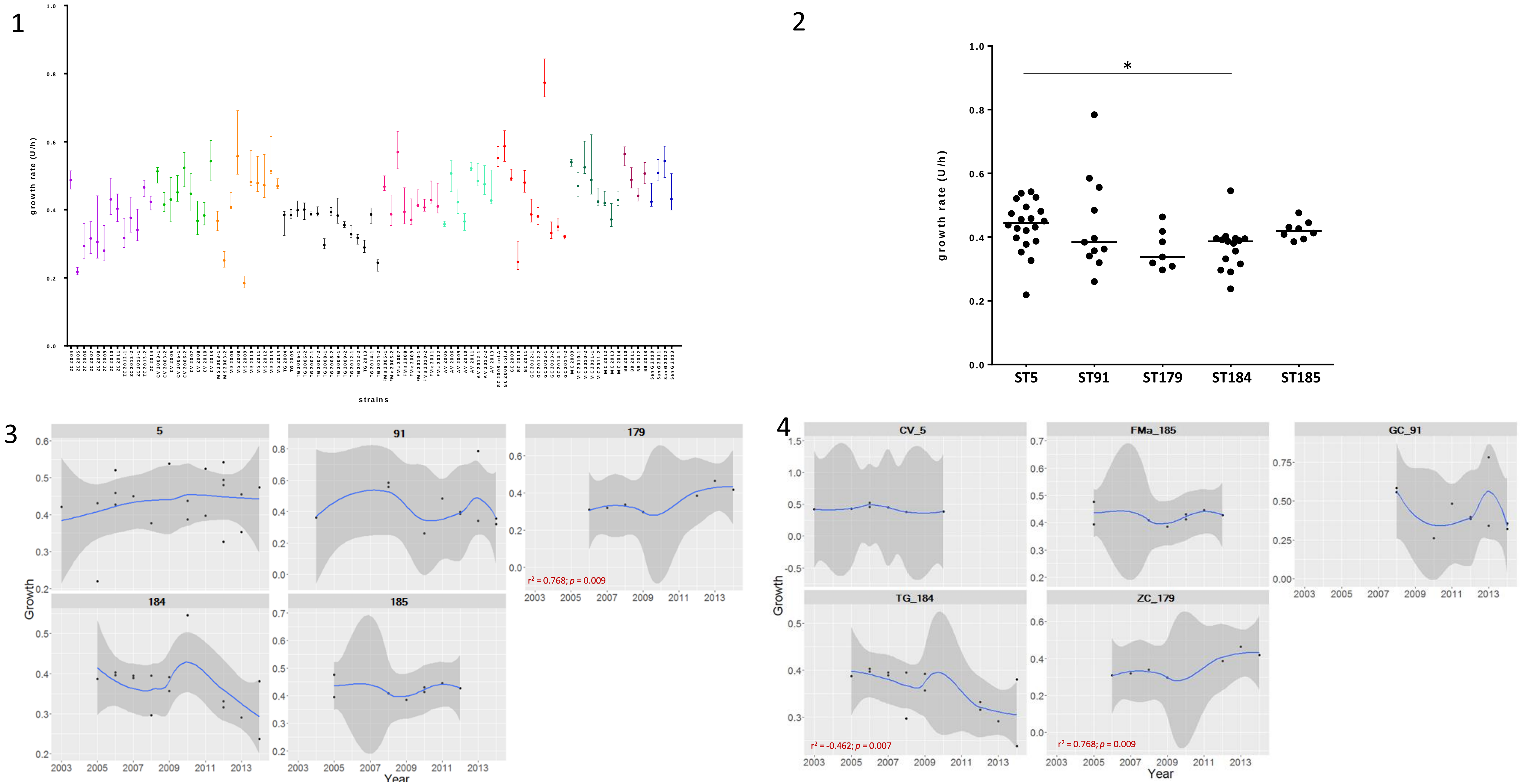

Supplementary Figure 6b. Growth rate of *S. maltophilia* strains collected over 12-year period from 10 CF patients. A) Overall growth rate. Results are medians with interquartile range. B) Growth rate values stratified on selected STs. Horizontal lines are median values. * $p<0.05$, Kruskall-Wallis + Dunn's multiple comparison post-test. C) Temporal trend of growth rate according to selected STs. D) Temporal trend of growth rate in each «ST-patient» combination. The statistical significance of temporal trends was assessed by linear regression.

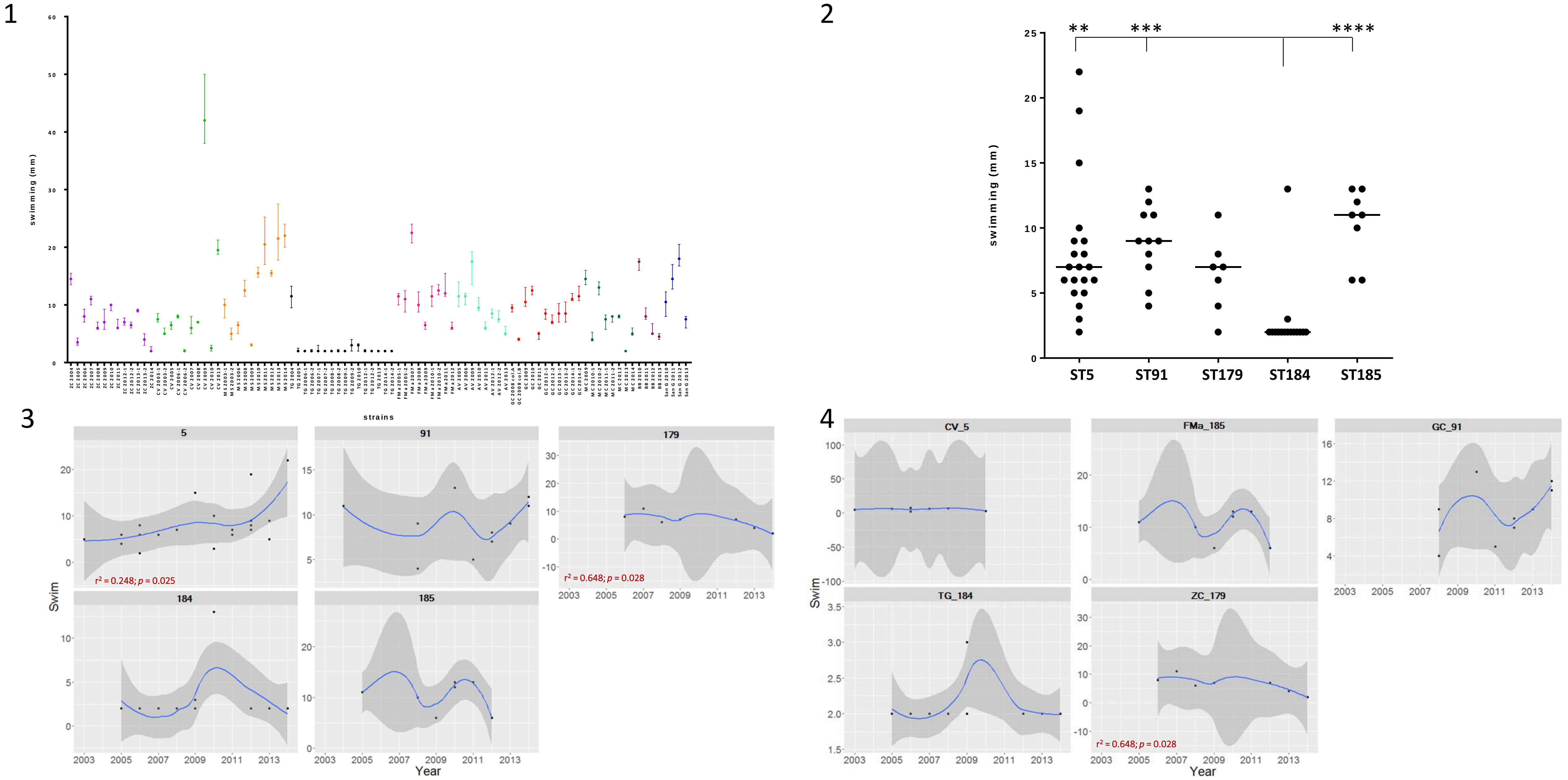

Supplementary Figure 6c. Swimming motility of *S. maltophilia* strains collected over 12-year period from 10 CF patients. A) Overall motility. Results are median + interquartile range. B) Motility stratified on selected STs. Horizontal lines are median values. ** p<0.01, *** p<0.001, **** p<0.0001, Kruskall-Wallis + Dunn's multiple comparison post-test. C) Temporal trend of swimming motility according to selected STs. D) Temporal trend of swimming motility in each «ST-patient» combination. The statistical significance of temporal trends was assessed by linear regression.

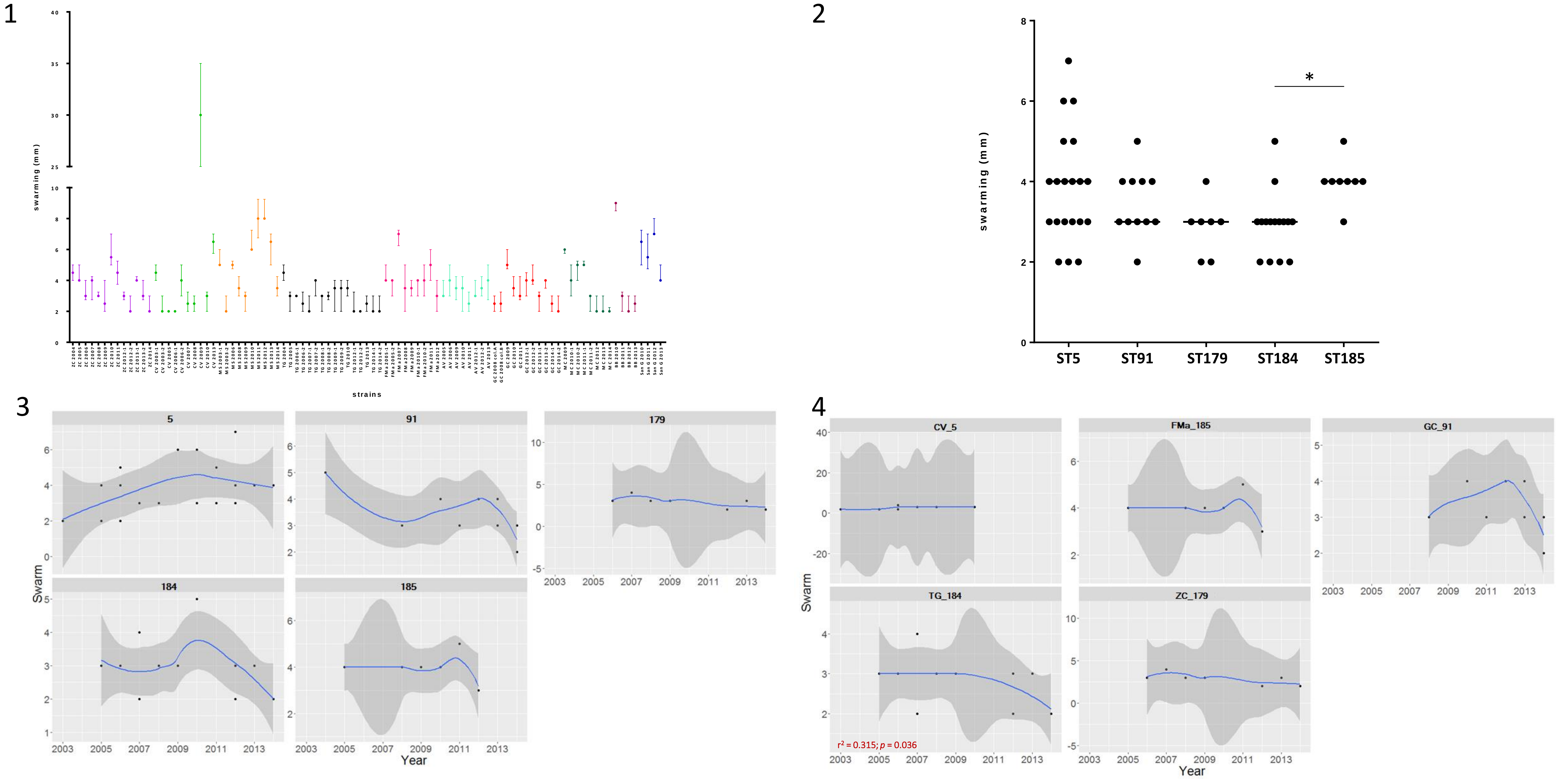

Supplementary Figure 6d. Swarming motility of *S. maltophilia* strains collected over 12-year period from 10 CF patients. A) Overall motility. Results are median + interquartile range. B) Motility stratified on selected STs. Horizontal lines are median values. * p<0.05, Kruskall-Wallis + Dunn's multiple comparison post-test. C) Temporal trend of swarming motility according to selected STs. D) Temporal trend of swarming motility in each «ST-patient» combination. The statistical significance of temporal trends was assessed by linear regression.

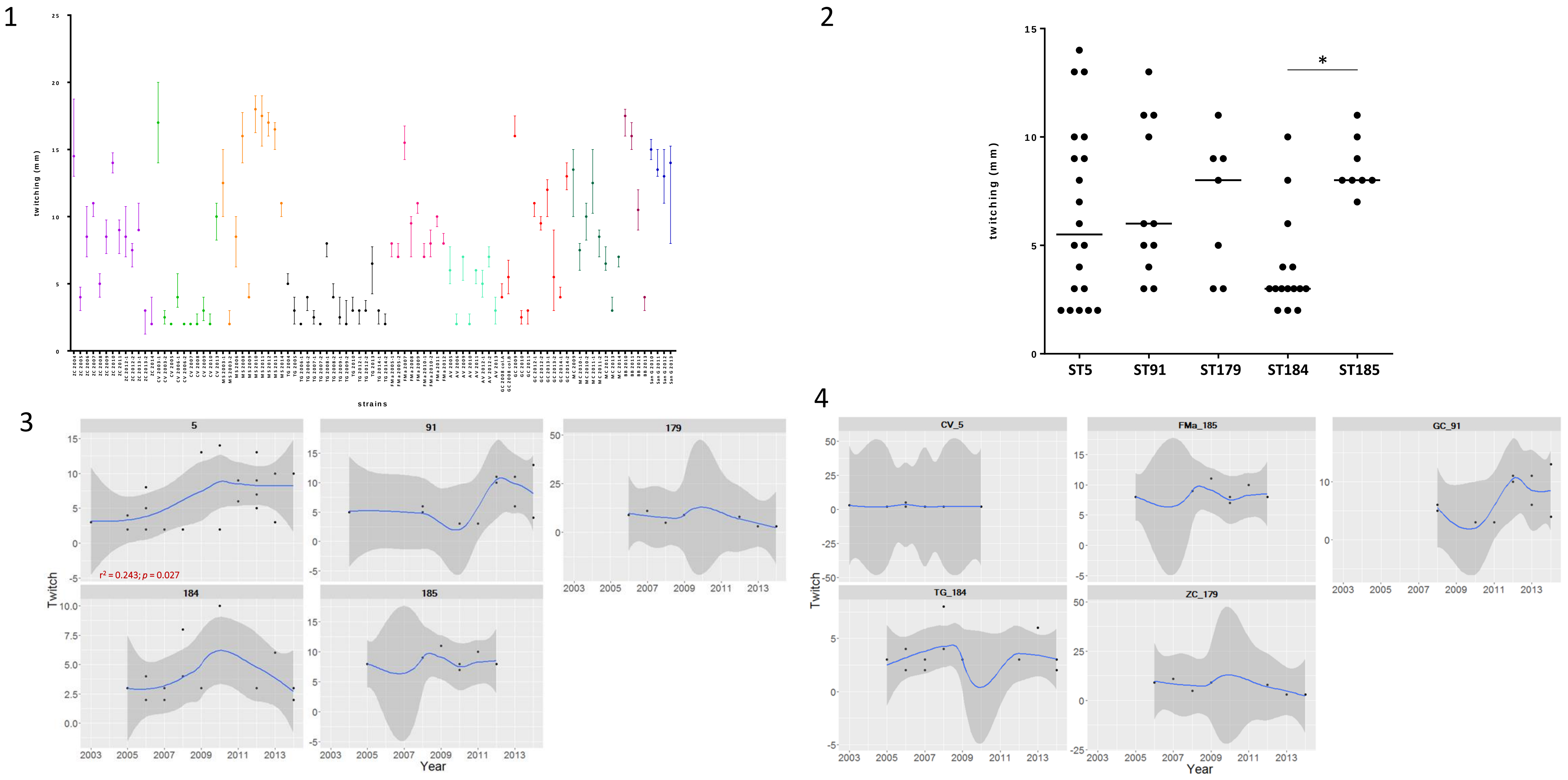

Supplementary Figure 6e. Twitching motility of *S. maltophilia* strains collected over 12-year period from 10 CF patients. A) Overall motility. Results are median + interquartile range. B) Motility stratified on selected STs. Horizontal lines are median values. * p<0.05, Kruskall-Wallis + Dunn's multiple comparison post-test. C) Temporal trend of twitching motility according to selected STs. D) Temporal trend of twitching motility in each «ST-patient» combination. The statistical significance of temporal trends was assessed by linear regression.

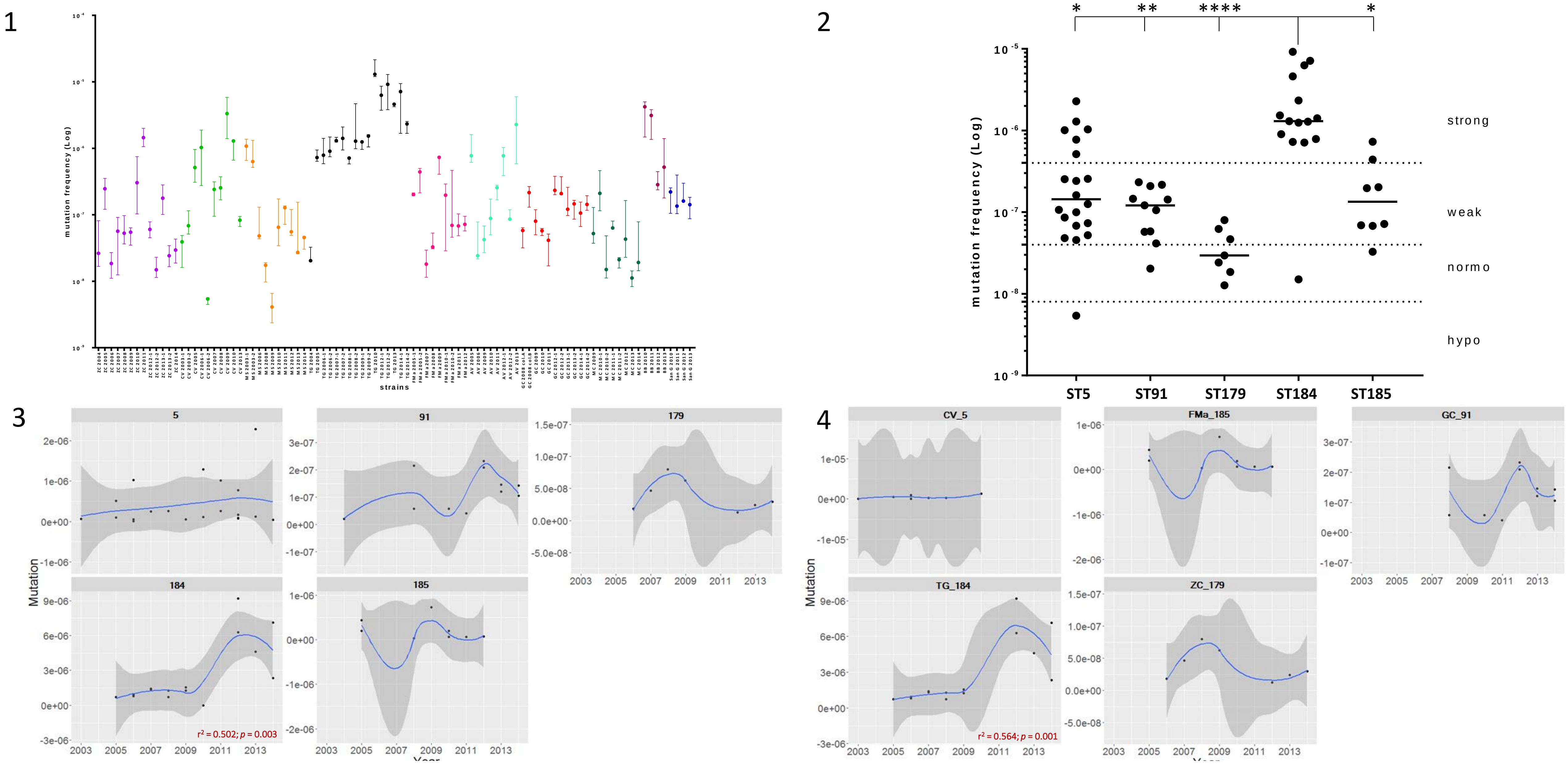

Supplementary Figure 6f. Mutation frequency of *S. maltophilia* strains collected over 12-year period from 10 CF patients. A) Overall mutation frequency. Results are median + interquartile range. B) Mutation frequency stratified on selected STs. Horizontal lines are median values. Strains were classified into four categories based on mutation frequency (f): hypo-mutators ($f \leq 8 \times 10^{-9}$), normo-mutators ($8 \times 10^{-9} < f < 4 \times 10^{-8}$), weak-mutators ($4 \times 10^{-8} \leq f < 4 \times 10^{-7}$), and strong-mutators ($f \geq 4 \times 10^{-7}$). * p<0.05, ** p<0.01, **** p<0.001, Kruskall-Wallis + Dunn's multiple comparison post-test. C) Temporal trend of mutation frequency according to selected STs. D) Temporal trend of mutation frequency in each «ST-patient» combination. The statistical significance of temporal trends was assessed by linear regression.

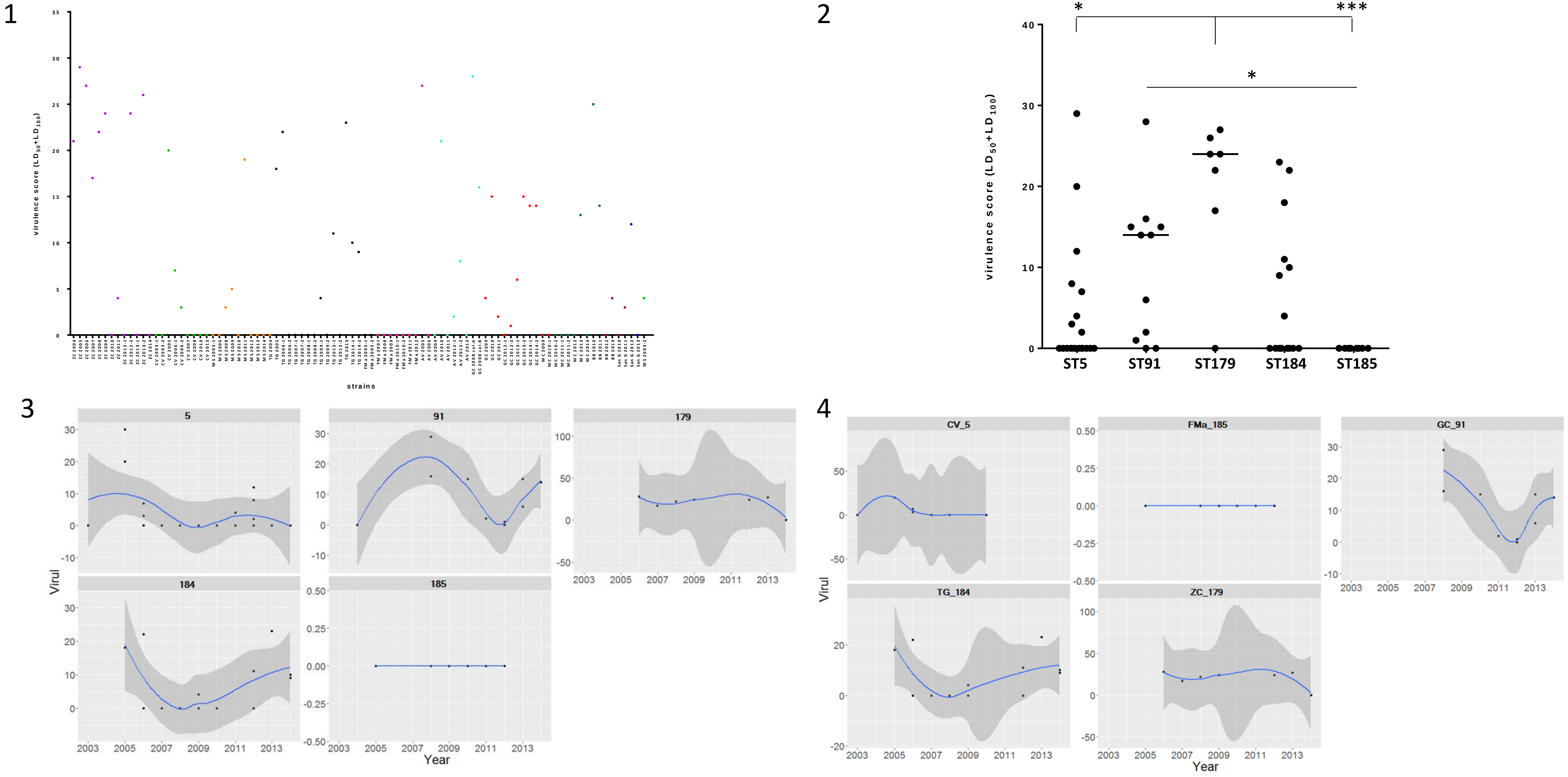

Supplementary Figure 6g. In vivo virulence, as assessed in *G. mellonella* infection model, of *S. maltophilia* strains collected over 12-year period from 10 CF patients. A) Overall virulence score. Results are median values. B) Virulence scores stratified on selected STs. Horizontal lines are median values. * $p<0.05$, *** $p<0.001$, Kruskall-Wallis + Dunn's multiple comparison post-test. C) Temporal trend of virulence according to selected STs. D) Temporal trend of virulence in each «ST-patient» combination. No statistically significant temporal trends was found by linear regression.
