# Supplementary material for: Evolution of Stenotrophomonas maltophilia in Cystic Fibrosis Lung over Chronic Infection: A Genomic and Phenotypic Population Study
Source: Front Microbiol. 2017 Aug 28;8:1590. doi: 10.3389/fmicb.2017.01590 (PMC5581383; doi:10.3389/fmicb.2017.01590)
Supplement: Supplementary file 19 [file Image8.PDF]

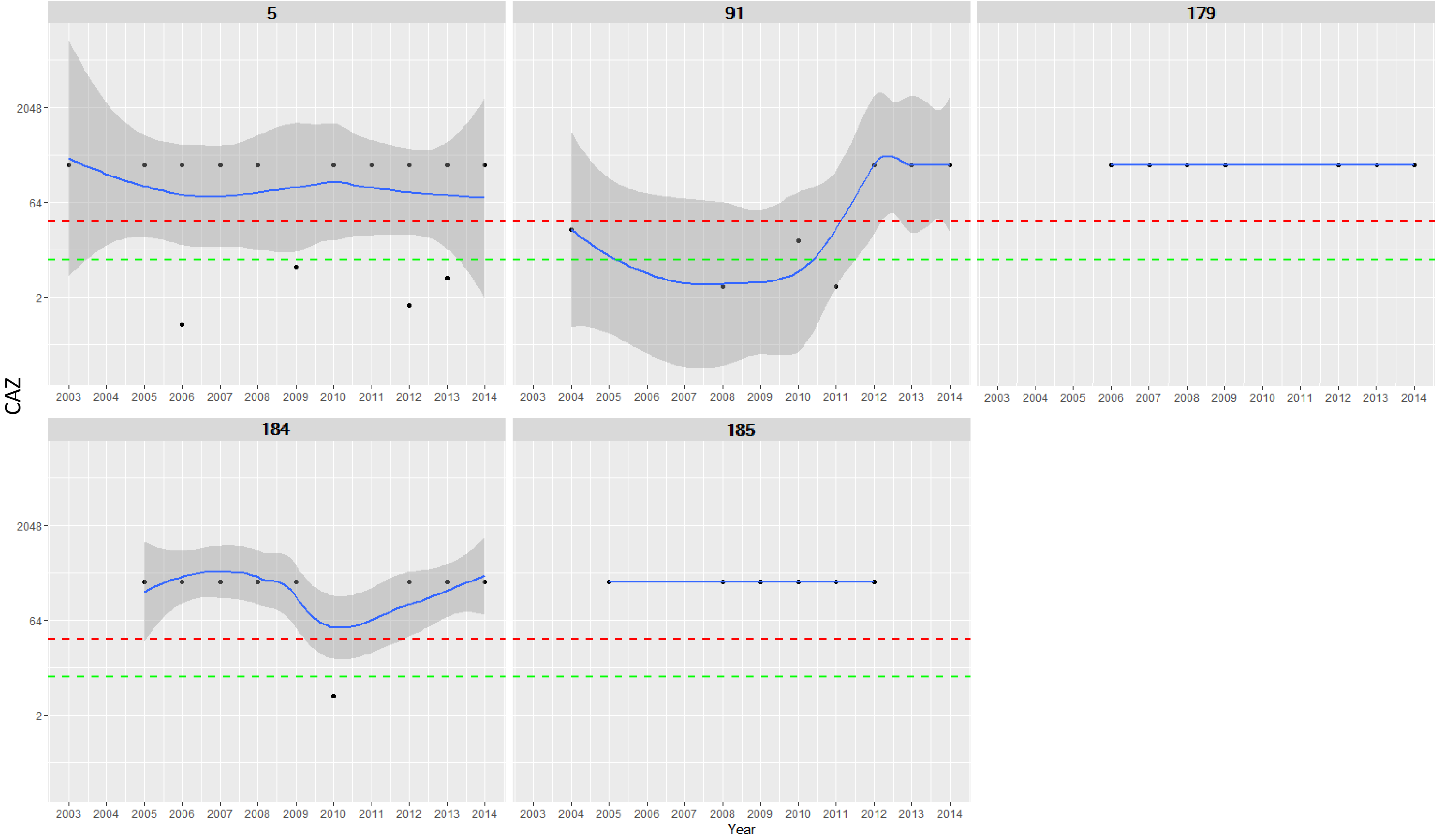

Supplementary Figure 8a. Temporal trend of Ceftazidime MIC values in selected STs. Dotted lines indicate breakpoint-MIC for resistance (red) and susceptibility (green), according to CLSI-guidelines.

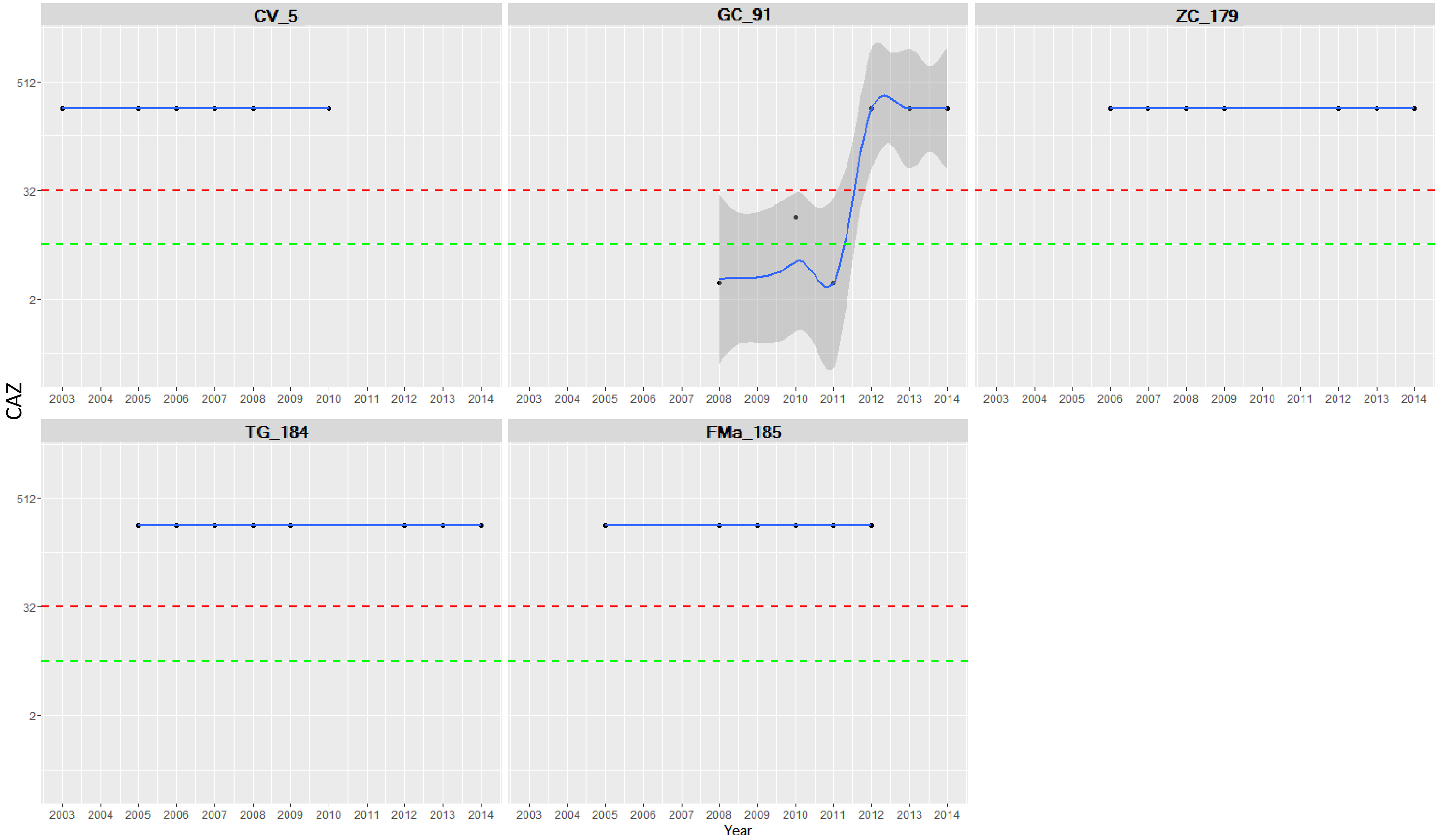

Supplementary Figure 8b. Temporal trend of Ceftazidime MIC values in selected «ST-patient» combinations. Dotted lines indicate breakpoint-MIC for resistance (red) and susceptibility (green), according to CLSI-guidelines.
